# Reversing Synchronized Brain Circuits Using Targeted Auditory-Somatosensory Stimulation to Treat Phantom Percepts: A Randomized Clinical Trial

**DOI:** 10.1001/jamanetworkopen.2023.15914

**Published:** 2023-06-02

**Authors:** Gerilyn R. Jones, David T. Martel, Travis L. Riffle, Josh Errickson, Jacqueline R. Souter, Gregory J. Basura, Emily Stucken, Kara C. Schvartz-Leyzac, Susan E. Shore

**Affiliations:** 1 Kresge Hearing Research Institute, Department of Otolaryngology, University of Michigan, Ann Arbor; 2 Department of Biomedical Engineering, University of Michigan, Ann Arbor; 3 Department of Molecular and Integrative Physiology, University of Michigan, Ann Arbor; 4 Department of Otolaryngology–Head and Neck Surgery, Medical University of South Carolina, Charleston; 5 Consulting for Statistics, Computing and Analytics Research, University of Michigan, Ann Arbor

## Abstract

**Importance:**

Animal models have shown altered dorsal cochlear nucleus circuitry in animals that develop tinnitus; however, precise treatment using bisensory (auditory and somatosensory) stimuli can reverse altered neural patterns and lessen tinnitus.

**Objective:**

To confirm and extend the findings of a pilot study, which suggested an increased efficacy of bisensory stimulation, to a clinical trial with a greater duration and greater number of participants.

**Design, Setting, and Participants:**

This double-blind, crossover, single-center randomized clinical trial was conducted from March 2019, with a 3-month follow-up per participant ending in July 2022. Eligible adults were recruited from the University of Michigan Health System in Ann Arbor, Michigan. Eligibility criteria included bothersome tinnitus (Tinnitus Functional Index [TFI] score, ≥17 points), somatic tinnitus, normal to moderate hearing loss, and no other tinnitus treatments in the 6 months prior to the trial. Included participants were randomized to either treatment group 1, which received active (bisensory) treatment, or group 2, which received the control (auditory-only) treatment. Results were analyzed using intent-to-treat (ITT) and per protocol (PP) populations.

**Intervention:**

Precisely timed bisensory (combined auditory and somatosensory) treatment was delivered through a portable, custom, take-home device that was provided to each participant for daily, at-home treatments. Group 1 participants received 30 minutes per day of the bisensory treatment for 6 weeks, followed by a 6-week washout phase, and then 30 minutes per day of the auditory-only treatment followed by a second 6-week washout phase. Group 2 participants received the auditory-only treatment first, followed by a washout phase, and then the bisensory treatment followed by a second washout phase.

**Main Outcomes and Measures:**

Primary end points were changes in TFI score and tinnitus loudness level from baseline through week 6 and week 12.

**Results:**

Of 337 screened individuals, 99 (mean [SD] age, 47 [12.7] years; 59 males [60%]; 85 with non-Hispanic White [86%] race and ethnicity) were enrolled into the study and randomized to treatment group 1 (n = 49) or group 2 (n = 50). The active but not the control treatment resulted in clinically significant decreases in TFI scores at week 6 of phase 1 (ITT population: –12.0 [95% CI, –16.9 to –7.9] points; *P* < .001; PP population: –13.2 [95% CI, –16.0 to –10.5] points; *P* < .001). Decreases in tinnitus loudness level were greater than 6 dB sensation level (SL; >half as loud) at week 6 for the bisensory treatment group, with little effect for the auditory-only treatment control group at week 6 of phase 1 (ITT population: –5.8 [95% CI, –9.5 to –2.2] dB; *P* = .08; PP population: –7.2 [95% CI, –11.4 to –3.1] dB; *P* = .03), and up to 11 dB SL at week 12 of phase 2 (ITT population: –10.9 [95% CI, –15.2 to –6.5] dB; *P* = .001; PP population: –14.1 [95% CI, –18.4 to –9.8] dB; *P* < .001). Decreased tinnitus loudness level and TFI scores extended into the washout phase, indicating a prolonged treatment effect.

**Conclusions and Relevance:**

This trial found that precisely timed bisensory treatment using stimuli and timing developed in a validated animal model was effective for adults with somatic tinnitus. Prolonged reduction in tinnitus symptoms can result from using an extended treatment duration.

**Trial Registration:**

ClinicalTrials.gov Identifier: NCT03621735

## Key Points

**Question:**

Does precisely timed bisensory (auditory and somatosensory) stimulation reduce tinnitus in humans with somatic tinnitus and does extended treatment lead to longer-lasting reductions in tinnitus symptoms?

**Findings:**

In this randomized clinical trial involving 99 participants with somatic tinnitus, statistically significant reductions in tinnitus loudness level as well as statistically significant and clinically meaningful reductions in Tinnitus Functional Index and Tinnitus Handicap Inventory scores were observed after bisensory treatment but not after auditory-only treatment. Bisensory treatment effect outlasted the treatment phase through the washout phase.

**Meaning:**

Findings from this trial suggest that 6 weeks of precisely timed bisensory treatment provides a lasting decrement in tinnitus for adults.

## Teaser

This randomized clinical trial examines the effects of unimodal and bimodal sound stimulation on tinnitus symptoms in adults.

## Introduction

Tinnitus, the perception of sound in the absence of external stimuli, occurs in approximately 15% of the US adult population, with 10% of these individuals reporting it as debilitating.^1^ Characteristic of most individuals with tinnitus, up to 80% can manipulate the volume, pitch, or tonal quality of their tinnitus by performing head (including face) or neck movements,^2,3^ termed *somatic tinnitus*. This group of individuals was chosen for this study as somatic tinnitus involves the somatosensory system, which has been shown to play an important role in the development of tinnitus.^4^ While there have been substantial advances in the understanding of neural mechanisms underlying tinnitus, the development of treatments has proven challenging, and there are currently no US Food and Drug Administration–approved treatments.

Animal-model studies have demonstrated that after cochlear damage, the neural circuitry of the dorsal cochlear nucleus (DCN) is altered, especially in animals that develop tinnitus.^5,6,7,8^ The DCN is the first central station of the auditory pathway that integrates auditory signals with sensory information originating in somatosensory ganglia and brainstem nuclei.^5,9,10,11,12^ Somatosensory projections synapse on cochlear nucleus granule cells whose axons form plastic synapses on fusiform-cell dendrites, in contrast to nonplastic synapses from the ear.^13,14^ Thus, repeated signals from the somatosensory system induce long-term plasticity in DCN neurons, and combined auditory and somatosensory signals can lead to either long-term depression (LTD) or long-term potentiation (LTP) of the fusiform-cell circuit, depending on the order and interval between the somatosensory and auditory signals (eFigure 1 in Supplement 1).^6,15,16^ After noise-induced tinnitus, guinea pigs demonstrated increased fusiform-cell spontaneous firing rates, bursting, and synchrony, which are important physiological correlates of tinnitus.^7^ Thus, it was hypothesized that properly timed auditory and somatosensory (bisensory) stimulation could be used to treat tinnitus by inducing LTD with precisely ordered and timed bisensory stimulation to reduce fusiform-cell synchrony and bursting, thereby reducing tinnitus.

To test this hypothesis, a previous study^8^ used noise overexposure to induce tinnitus in guinea pigs and determined an optimal bisensory interval for inducing LTD in the fusiform-cell circuit.^7^ An interval of −5 milliseconds (in which electrical stimulation followed auditory stimulation) resulted in the most consistent evidence of LTD. The optimal bisensory stimulation was applied to guinea pigs for 30 minutes per day for a 25-day treatment, resulting in a reduction in tinnitus in all animals. Recordings from fusiform cells confirmed that the bisensory stimulation significantly reduced spontaneous firing rates, bursting, and synchrony between fusiform cells, which correlated with the behavioral evidence of tinnitus. Since auditory stimuli alone did not generate long-term plasticity in the fusiform-cell circuit,^6,15,16^ auditory-only stimuli were used as a control treatment along with electrical-only or sedative-only control conditions. None of the controls significantly affected behavioral or physiological evidence of tinnitus in these animals.^7,8^

Based on preclinical animal studies,^6,7,8,15,16^ a translational study using bisensory stimuli with the same parameters was conducted to investigate the efficacy of bisensory stimulation for the treatment of humans with somatic tinnitus.^8^ Biphasic square-wave electrical stimulation was used to provide somatosensory stimulation, while each participant’s tinnitus was measured and recreated as a sound stimulus. In a double-blind, crossover randomized clinical trial, participants used a take-home device for 30 minutes per day for two 4-week phases interspersed with 4-week washout phases.^8^ One treatment phase delivered the active (bisensory) stimulation, while the other phase delivered the control (auditory-only) stimulation. During the active treatment phases, but not the control treatment phases, participants showed significant cumulative reductions in tinnitus loudness level and tinnitus distress,^8^ as measured by the Tinnitus Functional Index (TFI).^17^

The current study aimed to confirm and extend the findings of the pilot study^8^ to a clinical trial with a greater duration and greater number of participants. The previous study, which observed cumulative reductions in tinnitus over the 4-week treatment phases, suggested that increasing treatment duration would enhance treatment efficacy; thus, the current study design and bisensory treatment parameters were the same as those of the previous study except that the treatment phases were extended to 6 weeks. We hypothesized that participants receiving bimodal stimulation would show greater improvement in TFI score (range, 18-84, with the highest score indicating tinnitus is severly degrading quality of life), Tinnitus Handicap Inventory (THI) score (range, 8-84, with the highest score indicating tinnitus is severly degrading quality of life), and tinnitus loudness level than participants receiving auditory-only stimulation.

## Methods

### Study Design

**Figure 1.**
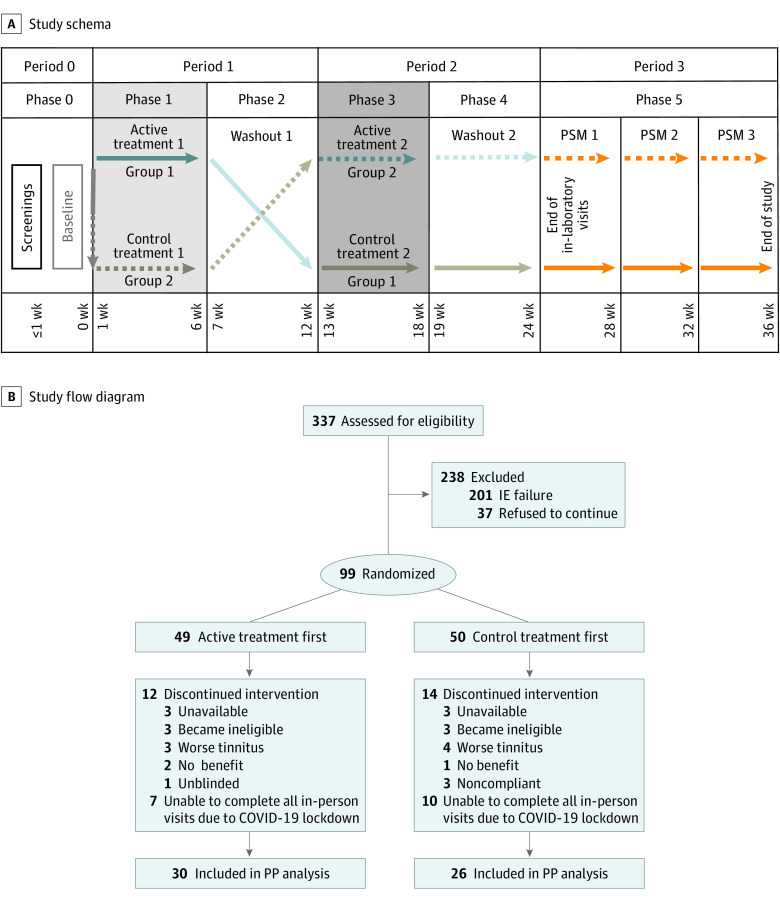
Study Schema and CONSORT Diagram IE indicates inclusion/exclusion; PP, per-protocol; PSM, poststudy monitoring.

Figure 1 shows the approved study design for the current randomized clinical trial and the study flow diagram. The University of Michigan Institutional Review Board approved the study protocol (Supplement 2). Participants provided their written consent for the trial to nonconflicted staff before assessment began. We followed the Consolidated Standards of Reporting Trials (CONSORT) reporting guideline.

Participants were randomized to treatment group 1 (received active treatment first then control treatment) or group 2 (received control treatment first then active treatment) in a 1:1 allocation for a balanced study design. In periods 1 and 2, each group received 6 weeks of active and control treatments (phases 1 and 3) followed by 6 weeks of washout phases (phases 2 and 4) in which no treatment was given but weekly primary end point measurements were taken. Period 3 encompassed extended follow-up of TFI score at weeks 28, 32, and 36 (phase 5) (Figure 1).

Data collection began in March 2019, with follow-up finishing in July 2022. Recruitment ended after 100 participants were enrolled (1 participant withdrew after enrolling but before receiving treatment). Due to the COVID-19 pandemic, study enrollment and in-clinic evaluations were paused from March through August 2020, resulting in incomplete data during this time. However, all enrolled participants were able to complete survey-based end points online.

### Eligibility Criteria and Recruitment

Adult participants (aged ≥18 years) with bothersome somatic tinnitus were recruited from the University of Michigan Health System in Ann Arbor, Michigan, or were private individuals who had directly contacted the principal investigator (S.E.S.). Eligibility criteria included bothersome tinnitus (TFI score, ≥17 points), somatic tinnitus, normal to moderate hearing loss, and no other tinnitus treatments in the 6 months prior to the trial. A total of 26 participants withdrew after being enrolled. Demographic data are provided in eTable 1 in Supplement 1, and inclusion or exclusion criteria and counts are shown in eTable 2 in Supplement 1. Race and ethnicity data were obtained from self-reported answers and included the following categories: American Indian, Asian, Black, White, other, unknown, or combinations (eTable 1 in Supplement 1).

Participants were medically assessed by ear, nose, and throat physicians prior to their enrollment and referred for further medical evaluation as needed. Initial participant assessment included otoscopy, detailed case history, extended high-frequency pure-tone audiometry (through 16 kHz), completion of the somatosensory modulation checklist (eTable 3 in Supplement 1), and TinnTester^18^ evaluation.

### Intervention

A portable, take-home treatment device was developed and manufactured by in2being LLC (eFigure 2 in Supplement 1). The device was programmed using a customized software package (Protocol Generator, Mathworks MATLAB; University of Michigan) to interface with other devices and to implement protocol treatment requirements. Somatosensory stimulation (3 biphasic square-wave pulses; 150 microseconds per phase; 1 millisecond between onsets) was provided by 2 transcutaneous active electrodes positioned on the skin overlying the trigeminal ganglion or the C1 and C2 cervical spinal cord with the ground electrode adjacent. Electrode placement was determined by somatic-maneuver evaluations.^19^ The current level was titrated to the participant’s sensation threshold. Open-circuit and short-circuit checks for all treatments were performed, with subthreshold electrical stimulation delivered every 10 seconds during the treatments. Auditory stimulation (10-millisecond duration; 1 millisecond rise-fall time) consisted of the participant’s tinnitus spectrum (TinnTester software suite^18^) set to a 40-dB sensation level (SL).

After training, participants used the device for 30 minutes per day for the duration of a treatment phase. Device use was logged and analyzed for compliance at every in-laboratory visit. Tinnitus was monitored throughout the study, and participants were withdrawn if both of their TFI scores for the same 2 consecutive weeks increased 12 points over the baseline level and if their mean tinnitus loudness level increased 16 dB (3 SDs from that in the pilot study,^8^ corresponding to an 85% increase in tinnitus loudness level).

### Control Treatment and Blinding

The treatment device was programmed to deliver either active or control stimulation based on the date of enrollment in the study and randomization number to maintain double-blinding (eFigure 2 in Supplement 1). To facilitate blinding during the control treatment (auditory only), participants were instructed to place the electrodes in the same position during both treatment phases and for both phases of the study. These 2 factors effectively blinded participants and study teams to the treatment phase (control vs active). In the pilot^8^ and the present studies, all participants reported quickly habituating to the electrical stimulus, which was close to threshold, making the stimulus difficult to detect. Participants stated that they could not tell whether they were receiving the control or active treatment.

### Statistical Analysis 

Within-participant changes from baseline in TFI score and tinnitus loudness level from the pilot study^8^ were extrapolated linearly from 4 to 6 weeks to estimate effect sizes. We hypothesized that participants receiving active treatments would show greater reductions in outcome measure slope than control participants. The mean difference in slopes determined that 37 participants would achieve a power of 0.8 for tinnitus loudness level and TFI score. A linear mixed-effects regression (LMER) model was developed, including random and fixed effects. Treatment order (received active treatment first vs received control treatment first), treatment condition (active, active washout, control, or control washout), baseline tinnitus loudness level, baseline TFI score, and interaction between treatment and treatment order were treated as fixed, whereas participant identification was included as a random effect. Because there was a carryover effect, only period 1 was used for analysis.

Age at enrollment, sex, and COVID-19 lockdown time (before, during, or after resumption of in-laboratory activities) were controlled for as covariates. Continuous variables were summarized by the means and SDs or 95% CIs. Treatment phase was used as the within-participant factor, and group was used as the between-participant factor to compare differences in the means between treatment groups. In addition to the coefficients reported by the LMER model, between-group changes within a treatment phase were estimated via linear combinations of coefficients from the LMER model.

χ^2^ Tests of proportions were used to assess minimal clinically important difference in TFI score, and Pearson linear correlations were used to relate change in TFI score to change in tinnitus loudness over the study weeks. There were 2 analysis populations: intent to treat (ITT) and per protocol (PP). The ITT population consisted of all enrolled participants (n = 99). In contrast, the PP population consisted of all participants who (1) did not withdraw for any reason, (2) had completed all in-person end point measurements before COVID-19-related laboratory shutdowns, or (3) had their baseline visit after the resumption of in-laboratory testing. Of the PP population, 30 participants received active stimulation during period 1, whereas 26 received control stimulation.

Two-sided *P* < .05 indicated statistical significance. Data analysis was performed with Mathworks MATLAB version 2022 (University of Michigan), and R, version 4.3 (R Foundation for Statistical Computing).

## Results

Of 337 individuals screened for eligibility, 99 (mean [SD] age, 47 [12.7] years; 59 males [60%], 40 females [40%]; 85 with non-Hispanic White [86%] and 14 with other [14%] race and ethnicity) were enrolled and randomized to treatment group 1 (n = 49) or group 2 (n = 50) (Figure 1; eTable 1 in Supplement 1). Of the 217 screened participants who could modulate with any maneuver (101 screen-failed somatic test; 19 could not modulate), more than 164 participants (75%) demonstrated a modulation score of 3 or higher for at least 1 jaw, head, or neck motion. Participants in both treatment groups were similar for all assessed demographic measures or end points at baseline (eFigure 3 in Supplement 1).

**Figure 2.**
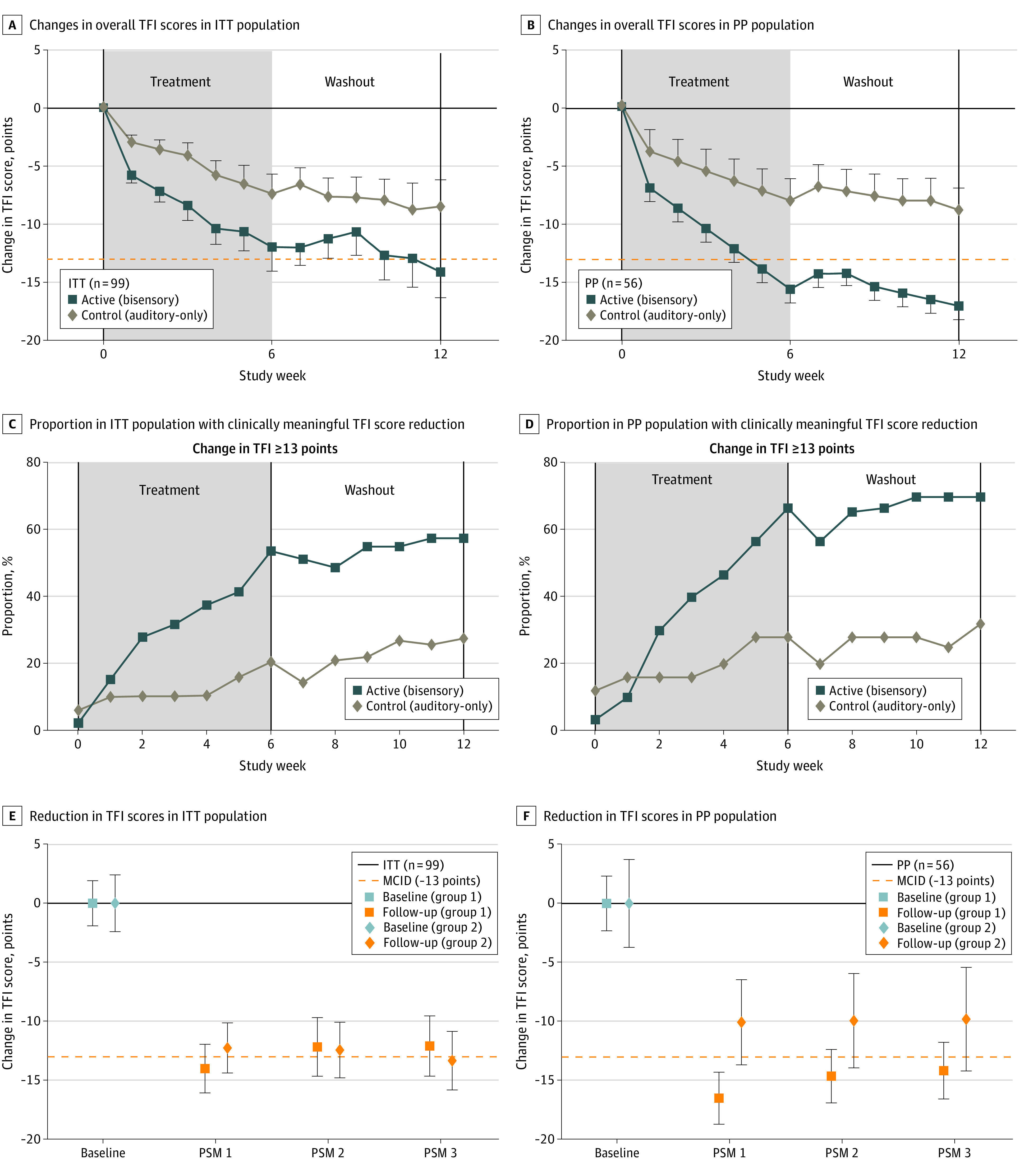
Tinnitus Functional Index (TFI) Scores in the Intent-to-Treat (ITT) and Per Protocol (PP) Populations The dashed orange lines in panels A and B indicate the minimal clinically important difference (MCID). Error bars represent the SEM. PSM indicates poststudy monitoring.

**Figure 3.**
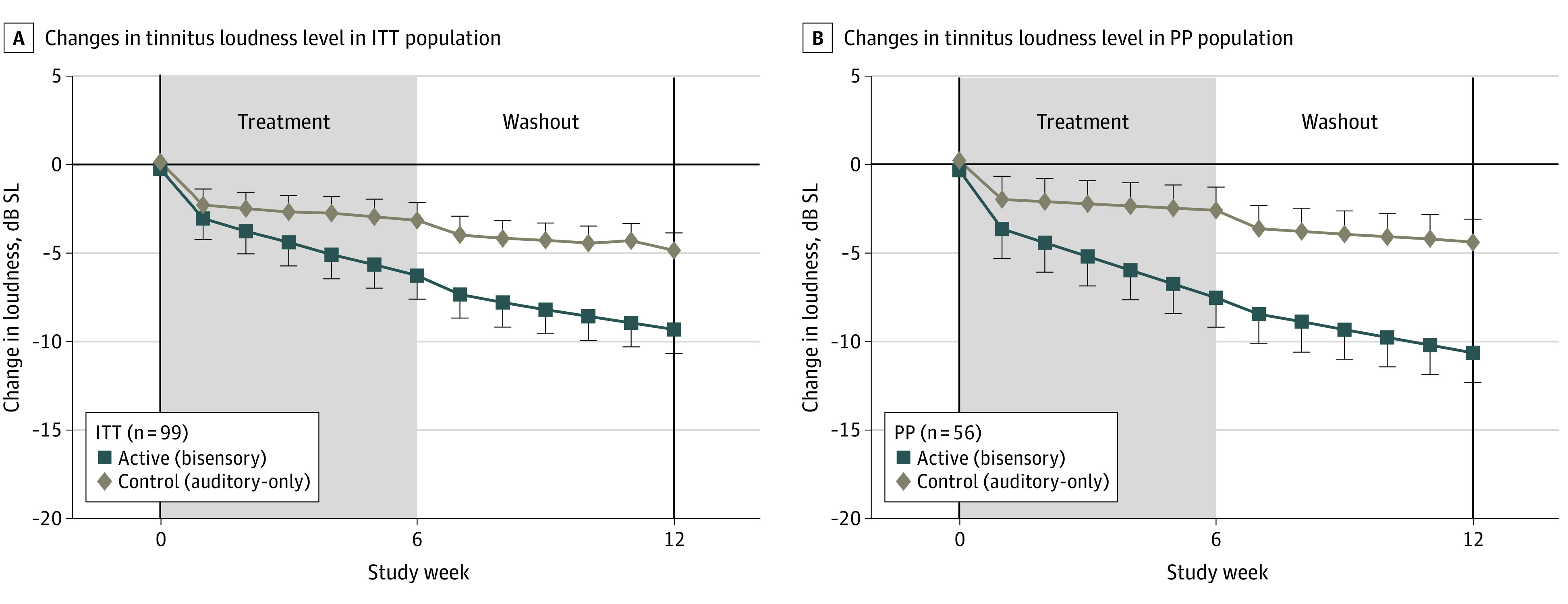
Tinnitus Loudness in the Intent-to-Treat (ITT) and Per Protocol (PP) Populations Error bars represent the SEM. dB SL indicates decibel sensation level. The solid horizontal line represents mean baseline value/normalized reference at week 0.

**Figure 4.**
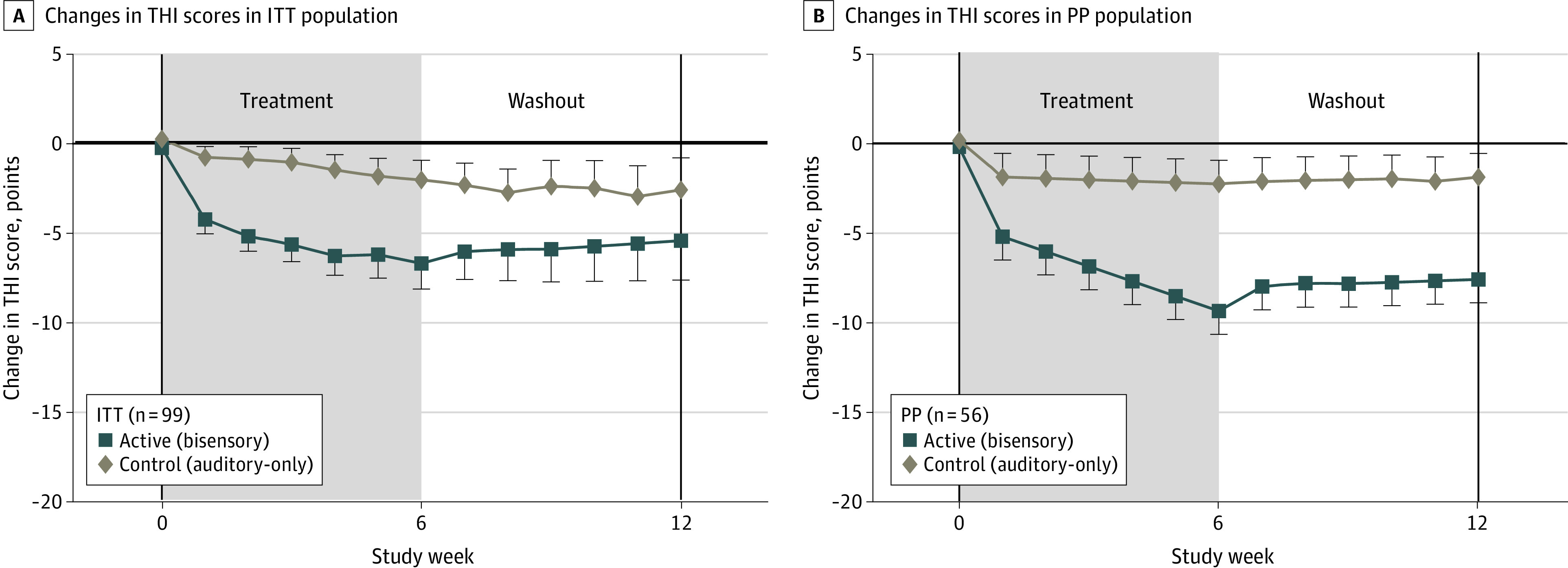
Tinnitus Handicap Inventory (THI) Scores in the Intent-to-Treat (ITT) and Per Protocol (PP) Populations Error bars represent the SEM. The solid horizontal line represents the mean baseline value/normalized reference at week 0.

Six weeks of active treatment (phase 1) resulted in a cumulative decrease in both overall TFI scores and tinnitus loudness levels. Unexpectedly, during the period 1 washout phase, there was a continued decline in TFI scores (ITT population: from –12.0 to –14.1 points; PP population: from –13.2 to –17.0 points) and tinnitus loudness levels (ITT population: from –5.8 to –9.2 dB SL; PP population: from –7.2 to –10.9 dB SL) (eFigures 4 and 5 in Supplement 1), producing a substantial carryover effect due to lack of recovery. Given the large carryover effect due to the longevity of the response, only period 1 was further analyzed per the standard procedure^20^ (Figures 2, 3, and 4).

Figure 2 shows changes in overall TFI scores (primary end point 1) for participants who received the active (bisensory) treatment vs the control (auditory-only) treatment. The ITT and PP population data are presented in Figure 2A and B, respectively. The active but not the control treatment resulted in clinically significant decreases in TFI scores at week 6 of phase 1(ITT population: –12.0 [95% CI, –16.9 to –7.9] points; *P* < .001; PP population: –13.2 [95% CI, –16.0 to –10.5 points; *P* < .001). While both the auditory-only and the bisensory treatments resulted in decreases in TFI scores, the bisensory treatment effect was significantly greater than that produced by the auditory-only treatment at week 6 of phase 1 (ITT population: –4.3 points [*P* < .001]; PP population: –6.8 points [*P* < .001]). Furthermore, the bisensory treatment resulted in TFI score reductions from baseline to 6 or 12 weeks, a difference that was greater than the minimal clinically important difference in TFI score of 13 points^17^ and that continued to decline during week 12 of the washout phase 2 for both the ITT (4.9 points; *P* = .002) and PP (–9.0 points; *P* < .001) populations.

Further analysis revealed that the proportion of participants who demonstrated a clinically meaningful reduction in TFI score (13 points) was significantly greater at week 6 of phase 1 of active treatment (PP population: 65% vs ITT population: 53%; PP population *P* = .003) compared with control treatment (PP population: 25% vs ITT population: 20%; ITT population *P* = .004) (Figure 2C and D). Group differences in proportions remained stable at week 12 of the washout phase 2 (ITT population: odds ratio, 2.12 [95% CI, 1.14-7.15]; *P* = .02; PP population: odds ratio, 1.97 [95% CI, 1.20-11.27]; *P* = .01). While the carryover effect hindered the analysis of period 2 results, we found that both groups (received active first then control treatment; received control first then active treatment) demonstrated long-term reductions in TFI scores. At 4, 8, and 12 weeks after weekly participation in the study, both groups showed a mean 13-point reduction in TFI scores (Figure 2E and F).

During the bisensory treatment, participants showed a similar cumulative decrease in tinnitus loudness level (primary end point 2) as shown by TFI scores, which continued decreasing during the washout phase (Figure 3). As observed for the TFI scores, there was some tinnitus loudness level decrease during the auditory-only treatment, but the decrease was minimal and significantly less than that during the bisensory treatment. Decreases in tinnitus loudness level were greater than 6 dB sensation level (SL; >half as loud) at week 6 for the bisensory treatment group, with little effect for the auditory-only treatment control group at week 6 of phase 1 (ITT population: –5.8 [95% CI, –9.5 to –2.2] dB; *P* = .08; PP population: –7.2 [95% CI, –11.4 to –3.1] dB; *P*=.03), and up to 11 dB SL at week 12 of phase 2 (ITT population: –10.9 [95% CI, –15.2 to –6.5] dB; *P* = .001; PP population: –14.1 [95% CI, –18.4 to –9.8] dB; *P* < .001). The tinnitus loudness level reduction for the active treatment was greater than 6 dB SL at week 6 of phase 1, which was more than half as loud as that for the control treatment (ITT population: –2.5 dB SL [*P* = .08]; PP population: –5.1 dB SL [*P* = .03]) and was as much as 11 dB SL greater at week 12 of phase 2 (ITT population: –4.5 dB SL [*P* = .001]; PP population: –7 dB SL [*P* < .001]).

As also demonstrated in the pilot trial,^8^ per-week changes in TFI scores and tinnitus loudness level correlated in the positive direction only during the active treatments for both the ITT group (Pearson linear correlation *r* = 0.16; *P* < .001) and the PP group (*r* = 0.17; *P* = .002). The per-week changes were not correlated during the control treatments for the ITT and PP populations (*r* = 3.6 × 10^−2^ [*P* = .42]; *r* = 4.5 × 10^−2^ [*P* = .39]) (eFigure 6 in Supplement 1).

As for the primary end points, there were significantly greater decreases in the THI score after the bisensory treatment but not the auditory-only treatment at weeks 6 and 12 (ITT population: mean difference, –4.0 [*P* = .049] and –4.4 [*P* = .051]; PP population: mean difference, –6.5 [*P* < .01]) and –6.0 [*P* = .018]) (Figure 4). Furthermore, the decreases were maintained during the washout phase and attained clinical significance of more than 5 points.

## Discussion

In this double-blinded randomized clinical trial of precisely timed bisensory (auditory-somatosensory) stimulation designed to induce LTD, a tinnitus-generating circuit, we hypothesized that tinnitus would be reduced in humans with somatic tinnitus. The bisensory treatment was compared with an auditory-only treatment, a condition that was not hypothesized to induce LTD into the circuit.^6,8,15^ Bisensory stimulation resulted in significant, cumulative, and lasting decreases in the primary end points of TFI score and tinnitus loudness level as well as the secondary end point of THI score. The clinically significant decreases in TFI score (>13 points) and THI score (>5 points) continued beyond the 6-week treatment duration and did not return to baseline during the 6-week washout but rather continued to decrease during the washout phase. Decreases in tinnitus loudness level showed the same pattern over time and correlated significantly with the TFI score decrements. The responder analysis showed that more than 65% of the PP population and more than 55% of the ITT population who received bisensory treatments had TFI score reductions that were clinically significant (≥13 points decrease from baseline) and remained constant during the active washout phase. Follow-up TFI questionnaires indicated an effect lasting up to 36 weeks. In all cases, the bisensory treatment showed a significantly greater effect compared with the auditory-only treatment, which showed slight decreases that were not clinically significant.

These results support the results of a previous pilot study with 20 participants,^8^ in which 4 weeks of treatment with the same stimuli used in the present study showed a continuous decrease in tinnitus loudness levels and TFI scores. After 4 weeks of treatment, the significant decreases in tinnitus loudness levels and TFI scores returned to baseline levels during the washout phases.^8^ In contrast, in the present study, the additional 2 weeks of treatment (6 weeks instead of 4 weeks) resulted in long-lasting effects that did not revert to baseline during the washout phase, inducing a significant carryover effect, which necessitated analysis of only period 1. Nonetheless, despite fewer data points, in both the ITT and PP populations significant differences were found between bisensory and unimodal treatments. Auditory-only stimulation was not hypothesized to reduce tinnitus based on cochlear nucleus circuitry (eFigure 1 in Supplement 1). That it did reduce tinnitus, even though only slightly and not clinically significantly, could be explained by 2 possible factors. First, the granule cell domain that projects to DCN fusiform-cell dendrites receives some projections from descending auditory pathways^21^ and thus might induce some plasticity in the circuit. However, as for unimodal somatosensory stimulation, a unimodal auditory long-term effect on DCN neurons is more likely to be through LTP than LTD.^6^ Consistent with this point were findings that somatosensory stimulation alone can worsen tinnitus in guinea pigs through LTP induction.^8^ However, in the same study,^8^ unimodal auditory stimulation did not alter tinnitus in guinea pigs, but humans showed a nonclinically significant decrease in tinnitus, as in the present study.

Given that LTD in the DCN circuit is not apparent with auditory-only stimulation, the second possibility is that the unimodal effects in this study and the pilot study^8^ were placebo effects.^17^ The correlation between TFI scores and tinnitus loudness decreases during the active treatment but not the control treatment and supports the possibility of placebo contributions to the unimodal sound treatment effects. We included only participants who demonstrated a somatosensory component to their tinnitus in that they could modulate their tinnitus with a somatic maneuver. Studies on somatic tinnitus found that somatic tinnitus occurred in 60% to 80% of individuals with tinnitus.^3,22,23^ Among the maneuvers that resulted in a change in tinnitus (either increase or decrease in tinnitus loudness level), jaw or neck movements were predominant. Notably, 75% of screened participants demonstrated a modulation score of 3 or higher for at least 1 jaw, head, or neck motion. Furthermore, there was a significant association between TFI score improvements and the number of somatic maneuvers producing a change in tinnitus during the active treatment phase but not the control treatment or washout phases (eFigure 7 in Supplement 2). The association between somatic tinnitus strength and bisensory treatment outcome was not surprising, as the treatment was based on auditory-somatosensory interactions in the brain and participants showing somatosensory involvement in their tinnitus were the participants of choice for this trial. Nonetheless, this finding does not necessarily mean that participants who cannot modulate their tinnitus might not be able to benefit from this bisensory treatment given that animal studies have shown that the upregulation in somatosensory inputs to the cochlear nucleus after cochlear damage occurs in all animals, not just those that develop tinnitus.^5,24^

### Comparisons to Other Treatments

Other studies have used either unimodal or bimodal sound stimulation paradigms to treat tinnitus, with mixed outcomes. In a study by Hall et al,^25^ acoustic coordinated reset neuromodulation, a unimodal auditory stimulation paradigm that was hypothesized to desynchronize pathological brain activity,^26,27^ led to no significant improvement for participants who used it, which was consistent with animal-model studies that have reported auditory-only treatment to be ineffective in reducing tinnitus.^8^ Transcutaneous electrical stimulation of the tongue combined with auditory stimuli resulted in statistically but not clinically significant reduction in the TFI scores 9 to 12 weeks after receiving treatment.^28,29^ Changing the stimuli halfway through the treatment resulted in an additional mean reduction to reach a clinically significant decrease at the 12-week point.^28^ Spencer et al^29^ used transcutaneous electrical stimulation of the head, neck, or the temporomandibular joint combined with tone-burst auditory stimuli that were pitch-matched to the participants’ tinnitus frequency. The treatment led to a statistically but not clinically significant reduction in TFI score of 6.9 points 9 to 12 weeks after treatment. Of 29 participants, only 6 could modulate their tinnitus (eg, somatic tinnitus), and the study was insufficiently powered to compare the effect of bisensory stimulation on patients with vs without somatic tinnitus.^29^

None of these studies used a control or sham group or nontreatment group; thus, all participants were aware that they were receiving an active treatment, increasing the risk for placebo effects.^30^ In contrast, the current trial had a control or sham group that received auditory-only stimulation. In addition, the current study used a psychoacoustic tinnitus loudness measurement as a primary outcome measure in addition to the TFI and THI surveys. While the TFI and THI questionnaires are both validated and widely used in tinnitus research and are important measures of tinnitus distress, the addition of a robust psychoacoustic tinnitus loudness measurement further validated the perceived reductions in tinnitus distress surveys.^31,32,33,34^

The majority of withdrawals were due to the burden of coming on site for weekly visits (eTable 4 in Supplement 1). Most adverse events (98 of 101) were not severe and were not associated with any treatment arm (eTable 5 in Supplement 1). Participants were informed that the electrical stimulation would be faint or barely perceptible during treatment. All participants were reminded of the double-blinding of the treatment phase (active vs control); therefore, we did not know if they would feel stimulation at any given time during their treatment sessions.

### Limitations

This study had some limitations. It is likely that participants were excited at the prospect of a treatment for their symptoms and were searching for any possible sensations; thus, a placebo effect is certainly possible. However, we believed that electrical stimulation that was titrated for each participant was the most optimal parameter for this type of study or treatment to minimize the effects. Additionally, per anecdotal reports, the novelty of the study diminished relatively quickly, and the daily procedure of cleaning, applying, and sitting quietly for 30 minutes of treatment, removing, and cleaning became monotonous for many participants. It is possible that the placebo effect accounted for some of the observed benefit in addition to the unimodal and bimodal treatment effects. However, since we saw a significant difference in tinnitus reduction between the active and control treatment groups vs the sham treatment group in the initial phase of the trial, it is likely that the active treatment was the main driver of therapeutic benefit.

## Conclusions

This double-blinded, sham-controlled, randomized clinical trial found that precisely timed bisensory treatment, using stimuli and timing that were developed in a validated animal model to produce LTD, effectively reduced tinnitus in adults with bothersome somatic tinnitus. Prolonged reductions in tinnitus distress and tinnitus loudness were achieved by using an extended treatment duration.

## Notes

Supplement 1. **eFigure 1.** Simplified DCN Circuit Demonstrating Multimodal Integration **eFigure 2.** Bisensory Treatment Device and Stimuli **eFigure 3.** Treatment Group Characteristics Were Not Different at Baseline **eFigure 4.** Overall TFI Scores Decrease Cumulatively Over 6 Weeks of Bi-Sensory Treatment and Continue Into Washout Period **eFigure 5.** Loudness Decreased Cumulatively Over 6 Weeks of Bi-Sensory Treatment and Continued to Decrease in the Washout Period But Was Minimal During Sound-Alone Control Treatment **eFigure 6.** Changes in Loudness and TFI Scores Were Significantly Correlated During the Active Treatment (Pearson’s Linear Correlation; r[ITT]=0.136, *P*=3e-3; r[PP]=0.165, *P*=2e-3) But Not During the Control Treatment **eFigure 7.** Reduction in TFI Significantly Correlates With Number of Somatic Maneuvers Eliciting Any Change in Tinnitus During Active Treatment (Pearson’s Linear Correlation; r(ITT)=0.292, *P*=1.09e-2; r(PP)=0.33, *P*=1.02e-2), But Not Control or Either Washout Times **eTable 1.** Demographics Table **eTable 2.** Inclusion/Exclusion Criteria and Counts **eTable 3.** Somatic Tinnitus Modulation Checklist **eTable 4.** Rationale for Subject Withdrawal from Study **eTable 5.** Rate of Adverse Events (AEs) Were Not Significantly Different Between Active vs Sham

Supplement 2.
Trial Protocol and Statistical Analysis

Supplement 3.
Data Sharing Statement
