# Supplementary material for: Reversing Synchronized Brain Circuits Using Targeted Auditory-Somatosensory Stimulation to Treat Phantom Percepts: A Randomized Clinical Trial
Source: JAMA Netw Open. 2023 Jun 2;6(6):e2315914. doi: 10.1001/jamanetworkopen.2023.15914 (PMC10238951; doi:10.1001/jamanetworkopen.2023.15914)
Supplement: Supplement 1. — eFigure 1. Simplified DCN Circuit Demonstrating Multimodal Integration eFigure 2. Bisensory Treatment Device and Stimuli eFigure 3. Treatment Group Characteristics Were Not Different at Baseline eFigure 4. Overall TFI Scores Decrease Cumulatively Over 6 Weeks of Bi-Sensory Treatment and Continue Into Washout Period eFigure 5. Loudness Decreased Cumulatively Over 6 Weeks of Bi-Sensory Treatment and Continued to Decrease in the Washout Period But Was Minimal During Sound-Alone Control Treatment eFigure 6. Changes in Loudness and TFI Scores Were Significantly Correlated During the Active Treatment (Pearson’s Linear Correlation; r[ITT]=0.136, P=3e-3; r[PP]=0.165, P=2e-3) But Not During the Control Treatment eFigure 7. Reduction in TFI Significantly Correlates With Number of Somatic Maneuvers Eliciting Any Change in Tinnitus During Active Treatment (Pearson’s Linear Correlation; r(ITT)=0.292, P=1.09e-2; r(PP)=0.33, P=1.02e-2), But Not Control or Either Washout Times eTable 1. Demographics Table eTable 2. Inclusion/Exclusion Criteria and Counts eTable 3. Somatic Tinnitus Modulation Checklist eTable 4. Rationale for Subject Withdrawal from Study eTable 5. Rate of Adverse Events (AEs) Were Not Significantly Different Between Active vs Sham [file jamanetwopen-e2315914-s001.pdf]

Supplemental Online Content

Jones GR, Martel DT, Riffle TL, et al. Reversing synchronized brain circuits using targeted auditory-somatosensory stimulation to treat phantom percepts: a randomized clinical trial. *JAMA Netw Open*. 2023;6(6):e2315914. doi:10.1001/jamanetworkopen.2023.15914

eFigure 1. Simplified DCN Circuit Demonstrating Multimodal Integration

eFigure 2. Bisensory Treatment Device and Stimuli

eFigure 3. Treatment Group Characteristics Were Not Different at Baseline

eFigure 4. Overall TFI Scores Decrease Cumulatively Over 6 Weeks of Bi-Sensory Treatment and Continue Into Washout Period

eFigure 5. Loudness Decreased Cumulatively Over 6 Weeks of Bi-Sensory Treatment and Continued to Decrease in the Washout Period But Was Minimal During Sound-Alone Control Treatment

eFigure 6. Changes in Loudness and TFI Scores Were Significantly Correlated During the Active Treatment (Pearson's Linear Correlation; r[ITT]=0.136, *P*=3e-3; r[PP]=0.165, *P*=2e-3) But Not During the Control Treatment

eFigure 7. Reduction in TFI Significantly Correlates With Number of Somatic Maneuvers Eliciting Any Change in Tinnitus During Active Treatment (Pearson's Linear Correlation; r(ITT)=0.292, *P*=1.09e-2; r(PP)=0.33, *P*=1.02e-2), But Not Control or Either Washout Times

eTable 1. Demographics Table

eTable 2. Inclusion/Exclusion Criteria and Counts

eTable 3. Somatic Tinnitus Modulation Checklist

eTable 4. Rationale for Subject Withdrawal from Study

eTable 5. Rate of Adverse Events (AEs) Were Not Significantly Different Between Active vs Sham

This supplemental material has been provided by the authors to give readers additional information about their work.

© 2023 Jones GR et al. *JAMA Network Open.*

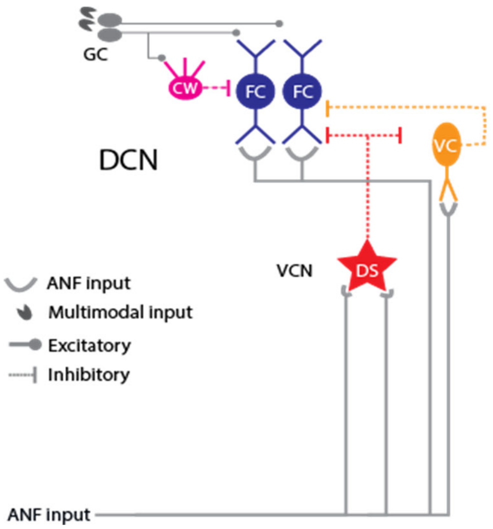

eFigure 1. Simplified DCN circuit demonstrating multimodal integration.

Multimodal input from somatosensory and other systems project to cochlear granule cells (GC). Granule cell axons, in turn, project to fusiform cell (FC) and cartwheel cell (CWC) apical dendrites, forming plastic synapses. Auditory nerve fibers (ANF) project to FC basal dendrites forming non-plastic synapses. FCs receive inhibitory input from D-Stellate (DS), vertical cells (VS) and CWCs.

Blinding and Randomization

Participants were randomly assigned to Group 1 (N=49; active first, then control) or Group 2 (N=50; control first, then active), in a 1:1 proportion for a balanced study design. The random number list was precomputed prior to the start of the study with a fixed, secret seed value (*randsample;* Mathworks, MATLAB statistics toolbox; Mersenne Twister algorithm). The seed value and subject assignment were then encrypted and only decrypted *in silico*. To verify each device's programmed state without breaking the blinding, each subject's enrollment number was associated with a unique 4-digit identifier derived from the initial programmed state and the subject's index in the randomization list. Successful programming was indicated by checking the equivalence of the 4-digit ID computed independently by at least two computers.

Intervention Implementation

The treatment device is shown in e**Figure 2a**. To generate a personalized auditory stimulus audiograms were converted from dB HL to dB SPL, zero-padded at frequency components outside of 200 Hz to 14 kHz, resampled, interpolated and smoothed at evenly spaced frequencies from 0 Hz to 24 kHz in 100 Hz steps. A subject's tinnitus likeness spectrum was assessed by having subjects titrate their tinnitus similarly (0-100 visual analogue Likert-scale) to a series of 11 iso-intensity sounds. Each sound was compared 3 times, with the resulting spectrum scaled to the SPL intensity used for the likeness test. The treatment spectrum (e**Figure 2B**) consisted of the tinnitus spectrum scaled to 40 dB SL above the audiogram spectrum and limited to an output no greater

© 2023 Jones GR et al. *JAMA Network Open.*

than 90 dB SPL at any frequency. The auditory spectrum was then converted into a time-domain signal using the Inverse Fast Fourier Transform (e**Figure 2C**). Sound stimuli were 10 ms in duration, 1 ms $\cos^2$ rise/fall time (MathWorks MATLAB Signal Processing Toolbox). The auditory stimulus was combined with the somatosensory stimulus using the interstimulus timing interval previously shown to suppress tinnitus in animals and humans (US PTO #3A9242067; Marks et al., 2018)

© 2023 Jones GR et al. *JAMA Network Open.*

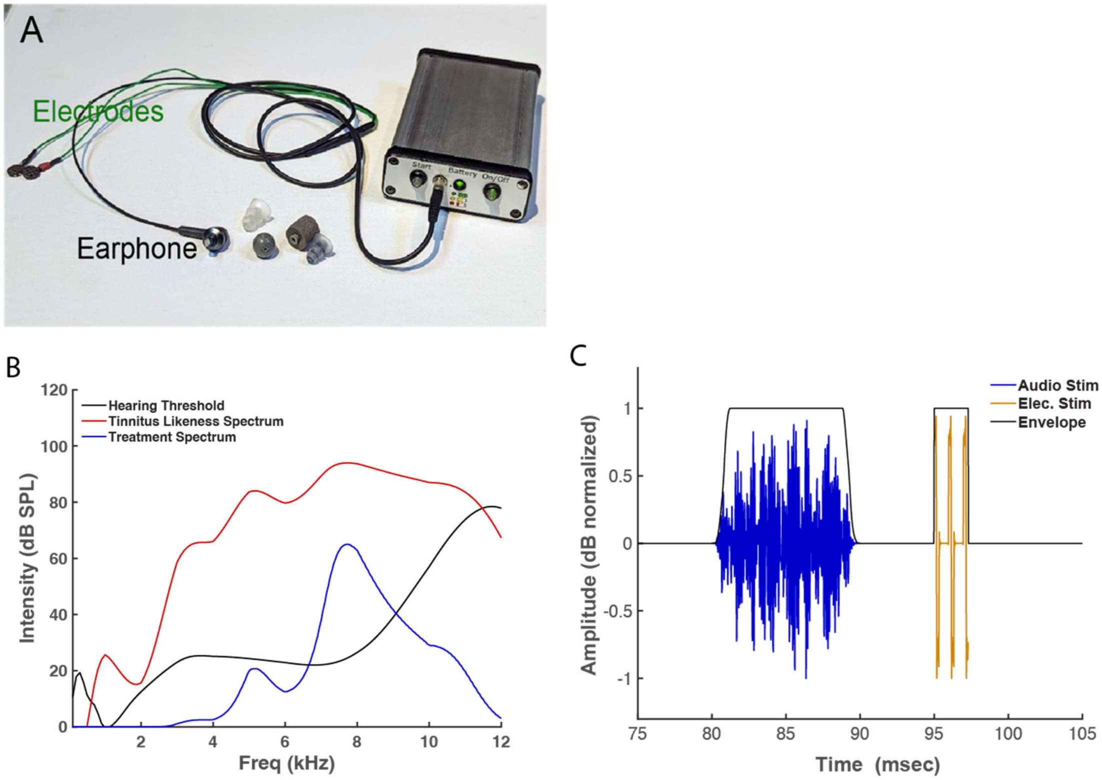

eFigure 2. Bisensory treatment device and stimuli.

A. The take-home device *is displayed with electrical leads (green) and audio lead (black).* ***B. Personalized treatment spectrum example*** *(blue) is calculated as a subject's tinnitus likeness (red) set at 40 dB above the subject's hearing threshold (black) in dB SPL.* ***C. Treatment stimulus****: Sample stimulus waveform is shown for one bisensory auditory-somatosensory stimulus.*

© 2023 Jones GR et al. *JAMA Network Open.*

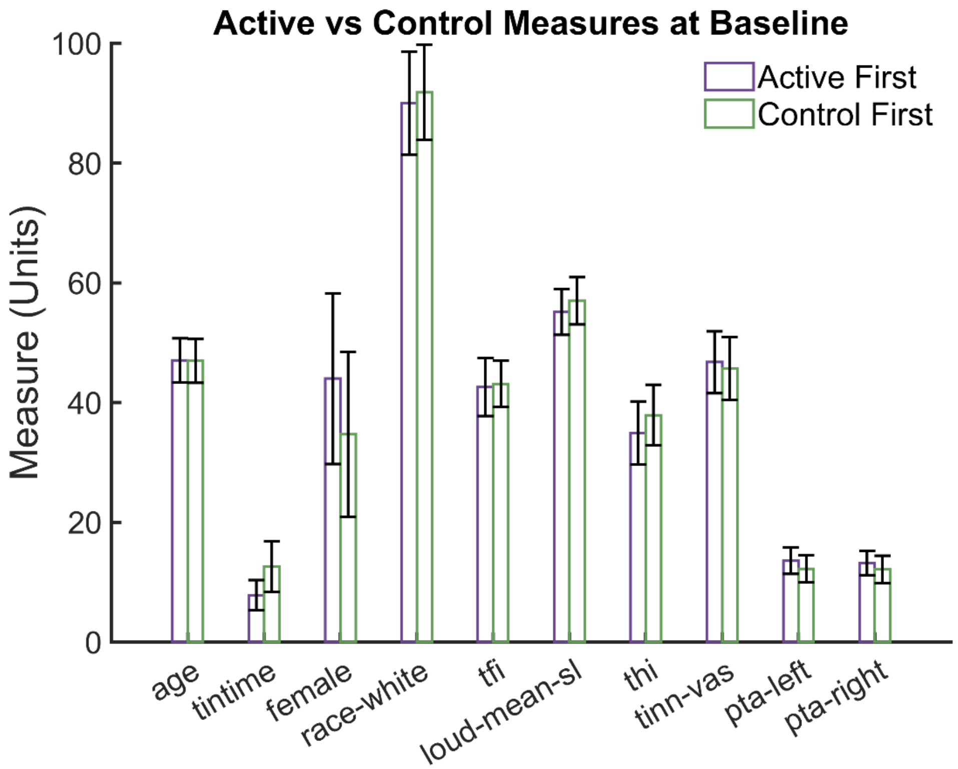

eFigure 3. Treatment group characteristics were not different at baseline.

Subjects (N=99) receiving bi-sensory-first (purple) vs control-first (green) were not significantly different for: age (years), tinnitus duration (years), female-identifying (%), white-race (%), TFI (points), TinnTester mean loudness (dB SL), THI (points), TinnTester VAS loudness (points), or left ear & right ear PTA threshold (dB SPL) (data shown: mean +/- 95% CI; p>0.05 between groups for all measures).

© 2023 Jones GR et al. *JAMA Network Open.*

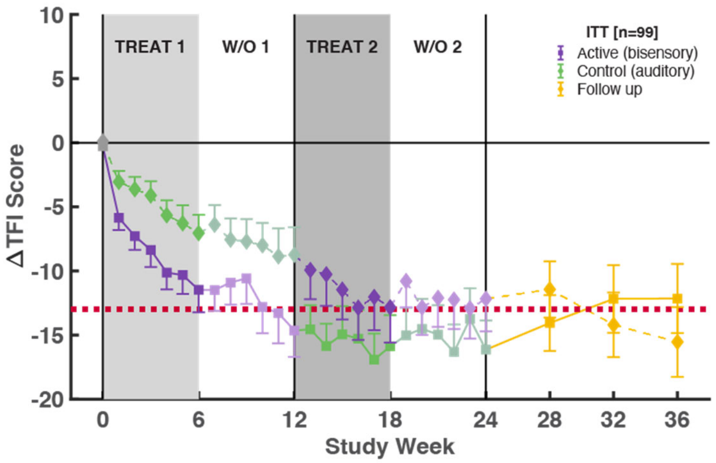

eFigure 4. Overall TFI scores decrease cumulatively over 6 weeks of bi-sensory treatment and continue into washout period.

Reduction in TFI during bi-sensory treatment and washout periods are significantly greater than decreases during the control, sound alone treatment and washout periods. Change in TFI shown for ITT group.

© 2023 Jones GR et al. *JAMA Network Open.*

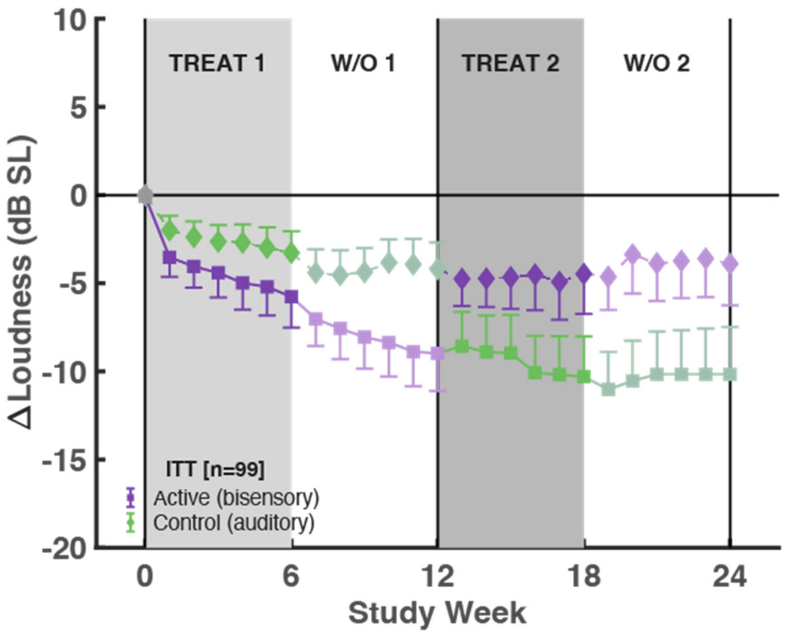

eFigure 5. Loudness decreased cumulatively over 6 weeks of bi-sensory treatment and continued to decrease in the washout period but was minimal during sound-alone control treatment.

Results are shown for ITT group.

© 2023 Jones GR et al. *JAMA Network Open.*

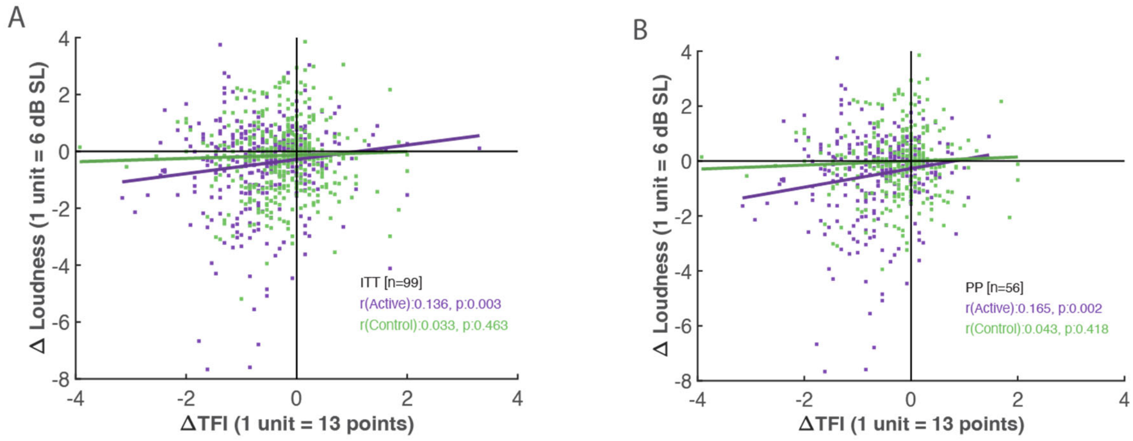

eFigure 6. Changes in Loudness and TFI scores were significantly correlated during the active treatment (Pearson's linear correlation; r[ITT]=0.136,p=3e-3; r[PP]=0.165,p=2e-3) but not during the control treatment**.**

A) *and* ***B)*** *show ITT and PP, respectively. The units displayed on this figure represent a clinically meaningful change in TFI Score (13 points) and a decrease in perceived loudness by half (6 dB).*

© 2023 Jones GR et al. *JAMA Network Open.*

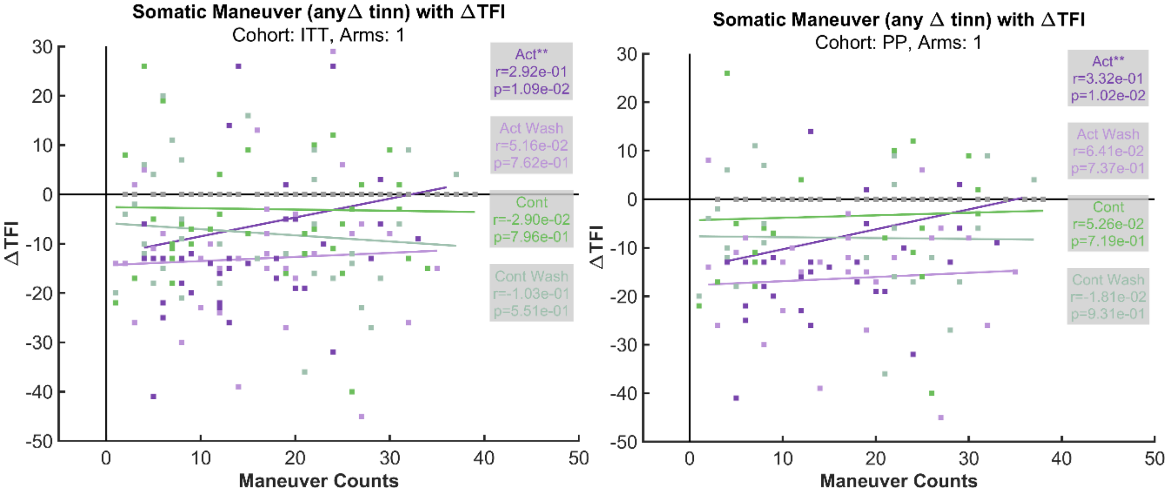

eFigure 7. Reduction in TFI significantly correlates with number of somatic maneuvers eliciting any change in tinnitus during active treatment (Pearson's linear correlation; r(ITT)=0.292, p=1.09e-2; r(PP)=0.33, p=1.02e-2), but not control or either washout times.

A) *ITT and* ***B)*** *PP cohorts, respectively.*

© 2023 Jones GR et al. *JAMA Network Open.*

eTable 1. Demographics Table.

Subjects in the present study had a similar demographic profile to the population of US tinnitus sufferers ((NCHS), 2011-2020).

Characteristic	Measure/Statistic	Study Population	US Tinnitus Population
**Hearing Loss (dB HL)**	**Pure Tone Average (Std. Dev.)**	9.8 ± 7.8 dB	13.1 ±8.6 dB
**Hearing Loss High Frequency (dB HL)**	**Pure Tone Average, >= 4kHz (Std. Dev.)**	25.5 ± 14.9 dB	28.0 ±14.6 dB
**Age**	**Mean (Std. Dev.)**	47 (12.7) years	54.7 (19.3) years
	**>=40 years**	63%	76%
**Sex**	**% Male**	60%	50.8%
**Race/Ethnicity**	**% White, non-Hispanic**	86%	46%

© 2023 Jones GR et al. *JAMA Network Open.*

eTable 2. Inclusion/exclusion criteria and counts.

Inclusion	Failures	Description
1	0	Must be 18 years of age or older
2	11	A score of >17 points on the Tinnitus Functional Index (TFI), as measured at the screening visit
3	5	Must report constant, subjective, preferably unilateral tinnitus without any active external or middle ear pathology
4	127	No greater than a moderate hearing loss at the tinnitus frequencies (≤55 dB HL) and no greater than a moderate hearing loss (≤50 dB HL) from 125 – 6,000 Hz
5	19	Must be able to modulate their tinnitus with a somatic maneuver (Simmons et al., 2008)
6	0	Preferably onset of tinnitus less than one year ago, but present for at least 6 months. Tinnitus should be bothersome (≥ 17 points, THI)
7	0	Absence of retrocochlear pathology
8	7	No participation in a tinnitus treatment regimen within the past six months or participation in the University of Michigan pilot clinical trial
9	2	Resides within 100 miles of the study site
10	33	Withdrew Consent / Lost to follow-up
**Exclusion**	**Failures**	**Description**
1	0	Anyone not meeting the above inclusion criteria, and in addition:
2	0	Diagnosis of Meniere's disease
3	1	Diagnosis of Semicircular Canal Dehiscence
4	0	Unilateral or bilateral cochlear implant recipients
5	0	Diagnosis of acoustic neuroma
6	7	Reports their tinnitus is pulsating
7	0	Evidence of retrocochlear disease
8	6	Patients with any indwelling electronic stimulation devices
9	3	Current or previous use of any acoustic hearing aid or over the counter personal sound amplification product (PSAP) in the last 6 months
10	13	Current or former use of any of the following medications within the past 6 months: high dose aspirin (>325mg/day), high dose NSAIDs (>800mg/day), narcotics (any opioids), lithium, clonazepam, oxazepam, cholinergic medications (anti-dementia medications), anti-depressant medications (serotonin specific reuptake inhibitors; SSRIs, tri-cyclic anti-depressants; TCAs); anti-seizure/convulsant medications (Depakote), anti-psychotic medications (Haldol, Seroquel), Ototoxic medications, other chemotherapy agents, benzodiazepines, and central nervous system stimulants
11	0	Current diagnoses of any of the following: obsessive compulsive disorder (OCD), schizophrenia, bipolar disorder, extreme generalized anxiety disorder, drug/alcohol dependence
12	0	Pregnant or nursing.

© 2023 Jones GR et al. *JAMA Network Open.*

eTable 3. Somatic Tinnitus Modulation Checklist.

Tinnitus Modulation Maneuver Checklist

		Significantly Better		Mildy Better		No Change		Mildly Worse		Significantly Worse	
CN III, IV, & VI	Left Gaze	☐ 1	☐ 2	☐ 3	☐ 4	☐ 5	☐ 6	☐ 7	☐ 8	☐ 9	☐ 10
	Right Gaze	☐ 1	☐ 2	☐ 3	☐ 4	☐ 5	☐ 6	☐ 7	☐ 8	☐ 9	☐ 10
	Up Gaze	☐ 1	☐ 2	☐ 3	☐ 4	☐ 5	☐ 6	☐ 7	☐ 8	☐ 9	☐ 10
	Down Gaze	☐ 1	☐ 2	☐ 3	☐ 4	☐ 5	☐ 6	☐ 7	☐ 8	☐ 9	☐ 10
	Up-right Gaze	☐ 1	☐ 2	☐ 3	☐ 4	☐ 5	☐ 6	☐ 7	☐ 8	☐ 9	☐ 10
	Up-left Gaze	☐ 1	☐ 2	☐ 3	☐ 4	☐ 5	☐ 6	☐ 7	☐ 8	☐ 9	☐ 10
	Down-right Gaze	☐ 1	☐ 2	☐ 3	☐ 4	☐ 5	☐ 6	☐ 7	☐ 8	☐ 9	☐ 10
	Down-left Gaze	☐ 1	☐ 2	☐ 3	☐ 4	☐ 5	☐ 6	☐ 7	☐ 8	☐ 9	☐ 10
CN V	Jaw Clench Bilateral	☐ 1	☐ 2	☐ 3	☐ 4	☐ 5	☐ 6	☐ 7	☐ 8	☐ 9	☐ 10
	Jaw Clench Left	☐ 1	☐ 2	☐ 3	☐ 4	☐ 5	☐ 6	☐ 7	☐ 8	☐ 9	☐ 10
	Jaw Clench Right	☐ 1	☐ 2	☐ 3	☐ 4	☐ 5	☐ 6	☐ 7	☐ 8	☐ 9	☐ 10
CN VII	Eyebrow Raise	☐ 1	☐ 2	☐ 3	☐ 4	☐ 5	☐ 6	☐ 7	☐ 8	☐ 9	☐ 10
	Tight Eyelid Closure	☐ 1	☐ 2	☐ 3	☐ 4	☐ 5	☐ 6	☐ 7	☐ 8	☐ 9	☐ 10
	Wide Smile	☐ 1	☐ 2	☐ 3	☐ 4	☐ 5	☐ 6	☐ 7	☐ 8	☐ 9	☐ 10
	Lip Purse	☐ 1	☐ 2	☐ 3	☐ 4	☐ 5	☐ 6	☐ 7	☐ 8	☐ 9	☐ 10
	Cheek Blow	☐ 1	☐ 2	☐ 3	☐ 4	☐ 5	☐ 6	☐ 7	☐ 8	☐ 9	☐ 10
CN XI	Neck Flexion (Passive)	☐ 1	☐ 2	☐ 3	☐ 4	☐ 5	☐ 6	☐ 7	☐ 8	☐ 9	☐ 10
	Neck Flexion (Active)	☐ 1	☐ 2	☐ 3	☐ 4	☐ 5	☐ 6	☐ 7	☐ 8	☐ 9	☐ 10
	Neck Flexion (Active w/ Resistance)	☐ 1	☐ 2	☐ 3	☐ 4	☐ 5	☐ 6	☐ 7	☐ 8	☐ 9	☐ 10
	Neck Extension (Passive)	☐ 1	☐ 2	☐ 3	☐ 4	☐ 5	☐ 6	☐ 7	☐ 8	☐ 9	☐ 10
	Neck Extension (Active)	☐ 1	☐ 2	☐ 3	☐ 4	☐ 5	☐ 6	☐ 7	☐ 8	☐ 9	☐ 10
	Neck Extension (Active w/ Resistance)	☐ 1	☐ 2	☐ 3	☐ 4	☐ 5	☐ 6	☐ 7	☐ 8	☐ 9	☐ 10
	Left Lat. Flexion (Passive)	☐ 1	☐ 2	☐ 3	☐ 4	☐ 5	☐ 6	☐ 7	☐ 8	☐ 9	☐ 10
	Left Lat. Flexion (Active)	☐ 1	☐ 2	☐ 3	☐ 4	☐ 5	☐ 6	☐ 7	☐ 8	☐ 9	☐ 10
	Left Lat. Flexion (Active w/ Resistance)	☐ 1	☐ 2	☐ 3	☐ 4	☐ 5	☐ 6	☐ 7	☐ 8	☐ 9	☐ 10
	Right Lat. Flexion (Passive)	☐ 1	☐ 2	☐ 3	☐ 4	☐ 5	☐ 6	☐ 7	☐ 8	☐ 9	☐ 10
	Right Lat. Flexion (Active)	☐ 1	☐ 2	☐ 3	☐ 4	☐ 5	☐ 6	☐ 7	☐ 8	☐ 9	☐ 10
	Right Lat. Flexion (Active w/Resistance)	☐ 1	☐ 2	☐ 3	☐ 4	☐ 5	☐ 6	☐ 7	☐ 8	☐ 9	☐ 10
	Left Rotation (Passive)	☐ 1	☐ 2	☐ 3	☐ 4	☐ 5	☐ 6	☐ 7	☐ 8	☐ 9	☐ 10
	Left Rotation (Active)	☐ 1	☐ 2	☐ 3	☐ 4	☐ 5	☐ 6	☐ 7	☐ 8	☐ 9	☐ 10
	Left Rotation (Active w/ Resistance)	☐ 1	☐ 2	☐ 3	☐ 4	☐ 5	☐ 6	☐ 7	☐ 8	☐ 9	☐ 10
	Right Rotation (Passive)	☐ 1	☐ 2	☐ 3	☐ 4	☐ 5	☐ 6	☐ 7	☐ 8	☐ 9	☐ 10
	Right Rotation (Active)	☐ 1	☐ 2	☐ 3	☐ 4	☐ 5	☐ 6	☐ 7	☐ 8	☐ 9	☐ 10
	Right Rotation (Active w/Resistance)	☐ 1	☐ 2	☐ 3	☐ 4	☐ 5	☐ 6	☐ 7	☐ 8	☐ 9	☐ 10
	Shoulder Shrug (Active)	☐ 1	☐ 2	☐ 3	☐ 4	☐ 5	☐ 6	☐ 7	☐ 8	☐ 9	☐ 10
	Shoulder Shrug (Active w/Resistance)	☐ 1	☐ 2	☐ 3	☐ 4	☐ 5	☐ 6	☐ 7	☐ 8	☐ 9	☐ 10
CN XII	Tongue Protrusion (Midline)	☐ 1	☐ 2	☐ 3	☐ 4	☐ 5	☐ 6	☐ 7	☐ 8	☐ 9	☐ 10
	Tongue Protrusion (Left)	☐ 1	☐ 2	☐ 3	☐ 4	☐ 5	☐ 6	☐ 7	☐ 8	☐ 9	☐ 10
	Tongue Protrusion (Right)	☐ 1	☐ 2	☐ 3	☐ 4	☐ 5	☐ 6	☐ 7	☐ 8	☐ 9	☐ 10

© 2023 Jones GR et al. *JAMA Network Open.*

eTable 4. Rationale for subject withdrawal from study.

Withdraw Reason	Counts
**By Self**	**16**
Device issues	1
Did not like sound of device	1
Increase in tinnitus	4
No benefit	1
General unrelated to study participation	9
**By Team**	**10**
Did not follow study team instruction	1
Increase in tinnitus	2
Lost to follow-up	1
Medication disqualifier	1
Neurological Twitch Comorbidity	1
Pulsatile Tinnitus	2
Unblinded	1
Major dental surgery	1
**Grand Total**	**26**

© 2023 Jones GR et al. *JAMA Network Open.*

eTable 5. Rate of Adverse Events (AEs) were not significantly different between Active vs Sham.

AE (#)	Non-severe	Severe	Total/Phase	Related	Unrelated	Total/Phase
Post-screen	2	0	2	0	2	2
Active	32	0	32	9	23	32
Active W/O	16	0	16	2	14	16
Control	24	2	26	6	20	26
Control W/O	21	1	22	1	21	22
PSM	3	0	3	0	3	3
**Total/Type**	**98**	**3**	**101**	**18**	**83**	**101**

Adverse Events (AEs) (*mild, moderate, and severe*) and device-relatedness (*0: unrelated; 1: probably unrelated; 2: potentially related; 3: probably related, and 4: related)* were scored by the clinical study members at the time of the event while blinded to active vs control status. Conflicted study members were prohibited from discussing AE severity or relatedness.

No serious AEs occurred during this clinical trial. Of 101 total AEs reported by 60 subjects, 3 AEs were classified as severe, all of which occurred during the Control or Control-Washout time periods. 18 AEs from 15 subjects were considered at least potentially associated with the treatment. The number of treatment-related AEs was not significantly different between Active/Active-washout (n=11) and Control/Control-Washout (n=7) periods (p=0.182, chi^2 test of proportions). AEs related to the subject's tinnitus were closely monitored. Subjects were removed from the study if their tinnitus loudness and TFI increased significantly for two consecutive weeks. Contextual factors, such as medication changes or external sound overexposures, were also considered when determining whether an AE was device related.

© 2023 Jones GR et al. *JAMA Network Open.*
