# Supplementary material for: Reversing Synchronized Brain Circuits Using Targeted Auditory-Somatosensory Stimulation to Treat Phantom Percepts: A Randomized Clinical Trial
Source: JAMA Netw Open. 2023 Jun 2;6(6):e2315914. doi: 10.1001/jamanetworkopen.2023.15914 (PMC10238951; doi:10.1001/jamanetworkopen.2023.15914)
Supplement: Supplement 2. — Trial Protocol and Statistical Analysis [file jamanetwopen-e2315914-s002.pdf]

Title: REVERSING SYNCHRONIZED BRAIN CIRCUITS WITH TARGETED AUDITORY-SOMATOSENSORY STIMULATION TO TREAT PHANTOM PERCEPTS – STAGE 2

Brief title: Targeted auditory-somatosensory tinnitus treatment

Protocol Number: HUM00143675

National Clinical Trial (NCT) Identified Number: NCT03621735

Principal Investigator: Susan Shore, PhD

Sponsor: National Institutes of Health (NIMH)

Funded by: NIMH RF1MH114244

Version Number: v.1.12

16 February 2022

Summary of Changes from Previous Version:

**Affected Section**	**Summary of Revisions Made**	**Rationale**
**1.2**	Updated both schemas in Section 1.2 of the protocol	- The schema on page 6 shows the End of Study visit occurring prior to the washout, but this visit happens after the washout, as shown in Section 1.3.<br><br>- The schema on page 7, in the Visit 15-28 Time Point section, states the final washout is 4 weeks, but it is actually 6 weeks, as shown in Section 1.3 and confirmed by the study team.
**1.1**	Added an exclusion criterion	- Added "pulsating" because this type of tinnitus is not what the

		device has been tested on with the animals.
**8.1**	Deleted "within 30 days."	- Give subjects more flexibility for when is best to begin the study with their schedule or if they need to wait on an MRI.
**6.1.2**	Added "Completion of Arm 1 and Arm 2 do not necessarily have to be consecutive, and we will allow for 12 weeks in between in order to provide more flexibility for subjects."	- Give subjects flexibility to remain in study, if they have to miss a chunk of time due to a family emergency, job, etc.
**8.1**	Added section under Specific Procedures: Otologic examination and medical case history regarding mental health identification	-noting that if mental health issues are identified during screening process, subjects will be referred back to their PCP for further evaluation and/or follow-up.
**7.3**	Added sentence defining missed visits and protocol deviation	-Clarifying that one missed visit per 6-week time period does not qualify a protocol deviation.
**5.5**	Added section detailing remote consenting	-allows subjects to consent remotely to reduce unnecessary in-person visits
**5.1**	Expanding hearing criteria	-slightly expanding inclusion/exclusion hearing criteria to include more subjects in the study
	Expanding consenting number from 300 to 400	-allows more subjects to be consented to reach goal for active subjects

	Expanding randomized subjects from 50 to up to 100	-some subjects that were enrolled during pandemic shutdown were not able to participate completely and have incomplete data, allows us to reach goal of 50 subjects with complete data
**7.1, 8.2**	Clarifying exclusion criteria	Clarifying exclusion criteria with TFI and TT
**8.2**	Adding online locations for consenting and medical screening, and 2nd floor of Med Sci I for medical screening, and Dr. Stucken for referrals as needed	Allows to meet online and prevent excessive in person appointments, allow Dr. Stucken to refer as needed
**8.2**	Updating adverse event reporting to match IRBMED	Updating adverse event reporting timeline from 7 days to 14 days to match IRBMED

Table of Contents

STATEMENT OF COMPLIANCE 1
1 PROTOCOL SUMMARY 1
1.1 Synopsis 1
1.2 Schema 5
1.3 Schedule of Activities (SoA) 8
2 INTRODUCTION 8
2.1 Study Rationale 8
2.2 Background 8
2.3 Risk/Benefit Assessment 10
2.3.1 Known Potential Risks 10
2.3.2 Known Potential Benefits 11
2.3.3 Assessment of Potential Risks and Benefits 11
3 OBJECTIVES AND ENDPOINTS 11
4 STUDY DESIGN 14
4.1 Overall Design 14
4.2 Scientific Rationale for Study Design 15
4.3 Justification for Dose 15
4.4 End of Study Definition 15
5 STUDY POPULATION 15
5.1 Inclusion Criteria 15
5.2 Exclusion Criteria 16
5.3 Lifestyle Considerations 17
5.4 Screen Failures 17
5.5 Strategies for Recruitment and Retention 17
6 STUDY INTERVENTION 18
6.1 Study Intervention(s) Administration 18
6.1.1 Study Intervention Description 18
6.1.2 Dosing and Administration 22
6.2 Preparation/Handling/Storage/Accountability 22
6.2.1 Acquisition and accountability 22
6.2.2 Formulation, Appearance, Packaging, and Labeling 22
6.2.3 Product Storage and Stability 23
6.2.4 Preparation 23
6.3 Measures to Minimize Bias: Randomization and Blinding 24
6.4 Study Intervention Compliance 24
6.5 Concomitant Therapy 24
6.5.1 Rescue Medicine 24
7 STUDY INTERVENTION DISCONTINUATION AND PARTICIPANT DISCONTINUATION/WITHDRAWAL 25

7.1 Discontinuation of Study Intervention 25
7.2 Participant Discontinuation/Withdrawal from the Study 25
7.3 Lost to Follow-Up........ 26
8 STUDY ASSESSMENTS AND PROCEDURES........ 26
8.1 Efficacy Assessments 26
8.2 Safety and Other Assessments........ 28
8.3 Adverse Events and Serious Adverse Events 30
8.3.1 Definition of Adverse Events (AE)........ 30
8.3.2 Definition of Serious Adverse Events (SAE)........ 31
8.3.3 Classification of an Adverse Event 31
8.3.4 Time Period and Frequency for Event Assessment and Follow-Up 33
8.3.5 Adverse Event Reporting........ 33
8.3.6 Serious Adverse Event Reporting 33
8.3.7 Reporting Events to Participants 34
8.3.8 Events of Special Interest 34
8.3.9 Reporting of Pregnancy........ 34
8.4 Unanticipated Problems........ 34
8.4.1 Definition of Unanticipated Problems (UP)........ 34
8.4.2 Unanticipated Problem Reporting 34
8.4.3 Reporting Unanticipated Problems to Participants........ 35
9 STATISTICAL CONSIDERATIONS........ 35
9.1 Statistical Hypotheses........ 35
9.2 Sample Size Determination 36
9.3 Populations for Analyses 36
9.4 Statistical Analyses........ 37
9.4.1 General Approach........ **Error! Bookmark not defined.**
9.4.2 Analysis of the Primary Efficacy Endpoint(s) 37
9.4.3 Analysis of the Secondary Endpoint(s)........ 37
9.4.4 Safety Analyses 38
9.4.5 Baseline Descriptive Statistics 38
9.4.6 Planned Interim Analyses 38
9.4.7 Sub-Group Analyses 38
9.4.8 Tabulation of Individual participant Data 38
9.4.9 Exploratory Analyses 38
10 SUPPORTING DOCUMENTATION AND OPERATIONAL CONSIDERATIONS........ 38
10.1 Regulatory, Ethical, and Study Oversight Considerations 38
10.1.1 Informed Consent Process........ 38
10.1.2 Study Discontinuation and Closure 39
10.1.3 Confidentiality and Privacy 39

10.1.4 Future Use of Stored Specimens and Data 40
10.1.5 Key Roles and Study Governance 40
10.1.6 Safety Oversight 41
10.1.7 Clinical Monitoring 41
10.1.8 Quality Assurance and Quality Control 41
10.1.9 Data Handling and Record Keeping 41
10.1.10 Protocol Deviations 42
10.1.11 Publication and Data Sharing Policy 42
10.1.12 Conflict of Interest Policy 43
10.2 Additional Considerations 43
10.3 Abbreviations 44
10.4 Protocol Amendment History 45
11 REFERENCES 47

STATEMENT OF COMPLIANCE

The Principal Investigator will assure that no deviation from, or changes to the protocol will take place without prior agreement from the funding agency and documented approval from the Institutional Review Board (IRB), except where necessary to eliminate an immediate hazard(s) to the trial participants.

The protocol, informed consent form(s), recruitment materials, and all participant materials will be submitted to the IRB for review and approval. Approval of both the protocol and the consent form must be obtained before any participant is enrolled. Any amendment to the protocol will require review and approval by the IRB before the changes are implemented to the study. All changes to the consent form will be IRB approved; a determination will be made regarding whether a new consent needs to be obtained from participants who provided consent, using a previously approved consent form.

1 PROTOCOL SUMMARY

1.1 SYNOPSIS

Title:

REVERSING SYNCHRONIZED BRAIN CIRCUITS WITH TARGETED AUDITORY-SOMATOSENSORY STIMULATION TO TREAT PHANTOM PERCEPTS – STAGE 2

Study Description: This study will assess the effectiveness of a bimodal auditory-somatosensory stimulation protocol in reducing the tinnitus loudness and life-impact in adults with tinnitus.

Objectives:

Primary Objective: to reduce tinnitus loudness and life impact.
Secondary Objectives: to alter the tinnitus spectrum minimum masking level

Endpoints:

Primary Endpoints:

1) Tinnitus Loudness: (TinnTester Interactive Software)

2) Tinnitus Functional Index (TFI) questionnaire

3) Tinnitus Handicap Inventory (THI)

Secondary Endpoints:

1)Tinnitus bandwidth/spectrum (TinnTester Interactive Software)

2) Tinnitus Hearing Survey (THS)

3) Minimum Masking Levels (MML)

Study Population:

Inclusion and Exclusion Criteria

Inclusion: 400 male or female, adult (18 years of age and older) subjects will be consented with a goal of including up to 100 subjects in the study**.**

1. Must be 18 years of age or older.
2. A score of >17 points on the Tinnitus Functional Index, as measured at the screening visit.
3. Must report constant, subjective, preferably unilateral tinnitus without any active external or middle ear pathology.
4. No greater than a moderate hearing loss at the tinnitus frequencies (≤55 dB HL) and no greater than a moderate hearing loss (≤50 dB HL) from 125 – 6000 Hz.
5. Must be able to modulate their tinnitus with a somatic maneuver
6. Preferably onset of tinnitus less than one year ago, but present for at least 6 months. Tinnitus should be bothersome.
7. Absence of retrocochlear pathology/ VIIIth nerve lesion

8. No participation in a tinnitus treatment regimen within the past six months or participation in the UM stage 1 clinical trial.
9. Resides within 100 miles of the study site.

Exclusion:

1. Anyone not meeting the above inclusion criteria, and in addition:
2. Diagnosis of Meniere's disease
3. Dianosis of Semicircular Canal Dehiscence
4. Unilateral or bilateral cochlear implant recipients
5. Diagnosis of acoustic neuroma
6. Reports their tinnitus is pulsating
7. Evidence of retrocochlear disease
8. Patients with any indwelling electronic stimulation devices
9. Current or previous use of any acoustic hearing aid or over the counter personal sound amplification product (PSAP) in the last 6 months
10. Current or former use of any of the following medications within the past 6 months: high dose aspirin (>325mg/day), high dose NSAIDs (ibuprofen, Motrin, Celebrex) (>800mg/day), narcotics (any opioids), lithium, clonazepam, oxazepam, cholinergic medications (anti-dementia medications), anti-depressant medications (serotonin specific reuptake inhibitors; SSRIs, tri-cyclic anti-depressants; TCAs); anti-seizure/convulsant medications (Depakote), anti-psychotic medications

(Haldol, Seroquel), Ototoxic medications, other chemotherapy agents, benzodiazepines, and central nervous system stimulants.

11. Current diagnoses of any of the following: obsessive compulsive disorder (OCD), schizophrenia, bipolar disorder, extreme generalized anxiety disorder, drug/alcohol dependence.
12. Pregnant or nursing.

Participant's eligibility will be assessed at an initial examination conducted by an otologist and audiologist consisting of otoscopy, audiometry, tinnitus modulation checklist, case history questionnaire, and the Tinnitus Functional Index (TFI) questionnaire.

**Phase:**	Phase I/II
**Description of Sites/Facilities Enrolling Participants:**	. One site – University of Michigan Kresge Hearing Research Institute
**Description of Study Intervention:**	Bimodal auditory-somatosensory (electrical) stimulation will be delivered to subjects using specially-fabricated programmable devices by In2being inc. that adhere to FDA regulations for De-novo devices with non-significant risk (as per pre-submission feedback from FDA). The devices will be programmed in the lab by our staff, and the somatosensory and auditory stimuli will be limited by hardware to safe and comfortable levels for the subjects.<br><br>The auditory stimulus, presented through a calibrated earphone, will simulate the tinnitus as estimated above at the tinnitus frequency or frequencies. The intensity of the auditory stimulus will be based on the presentation levels in the previous clinical trial that were found to be effective (originally based on preclinical data). The auditory stimulus level will be based on subjects' individual hearing and tinnitus profile, and will be restricted to their comfort range. The durations and timing of the auditory and somatosensory (electrical) stimulation will use preclinical study determined stimulus

timing and durations of stimulation (Marks et al, 2018, STM). The intensity of the electrical stimulation will be adjusted to be just below that which elicits mild muscle contractions. Somatosensory stimulation will be delivered by standard electrode pads positioned on the cheek overlying the trigeminal ganglion, the juncture of the temporomandibular joint or on the neck at overlying cervical nerves, c1 or c2, determined by the manner in which the subject can modulate the tinnitus. If the modulation alters tinnitus loudness or pitch, the position that causes reduction will be the position of choice. Subjects will be able to feel a slight tingling with the onset of the electrical stimulation, which usually fades away after a few minutes.

To mitigate potential placebo effects, subjects will receive both a sham treatment and an active treatment. The subjects will be blinded to which treatment they are receiving. Subjects will be told that they may receive different treatment protocols, some of which are expected to be ineffective. While subjects will be informed that they will receive both the active and the sham treatment, they will not be told which treatment they are receiving. As the sham treatment is using the same device as the active treatment, there will be no additional risks to the subject.

Treatment durations: Daily, 30- 60 minutes. Devices will programmed to take home for 30-60 minutes per day for 6 weeks for each arm of the study (treatment and sham).

Study Duration: 4 years
Participant Duration: 10 months

1.2 SCHEMA

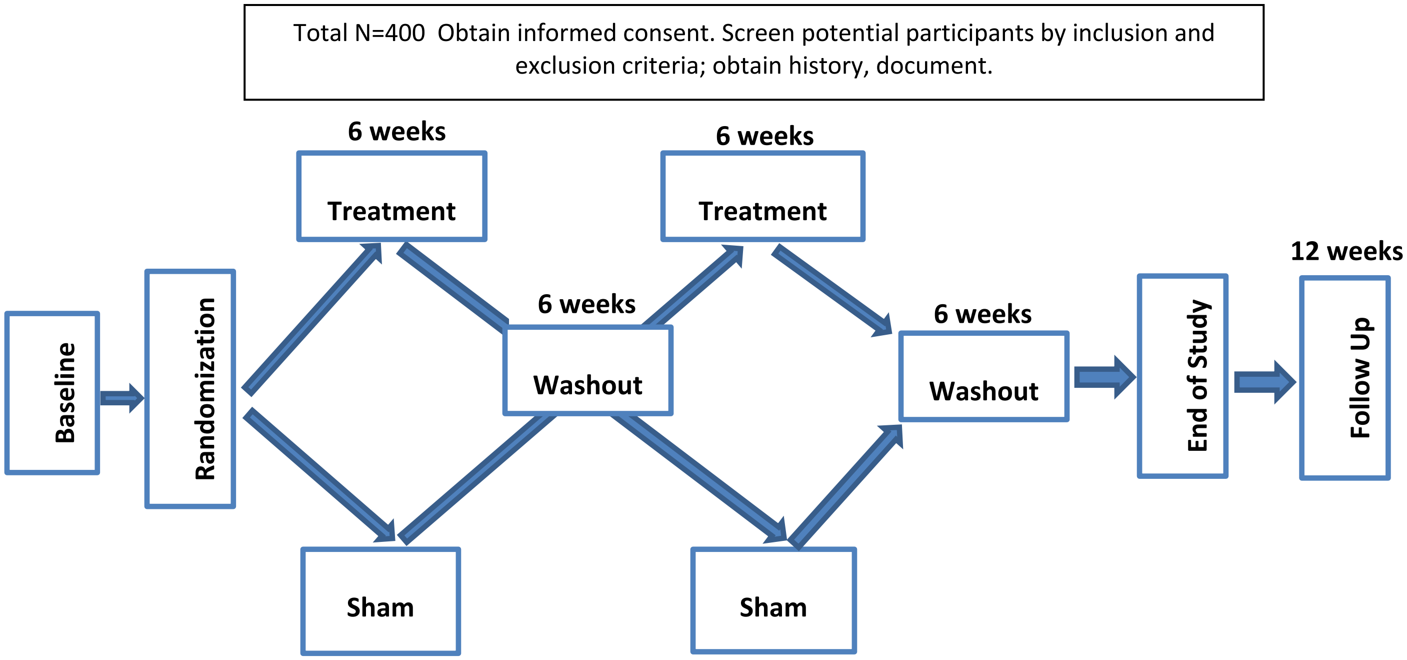
Total N=400 Obtain informed consent. Screen potential participants by inclusion and exclusion criteria; obtain history, document.
Baseline
Randomization
6 weeks
Treatment
Sham
6 weeks
Washout
6 weeks
Treatment
Sham
6 weeks
Washout
End of Study
12 weeks
Follow Up

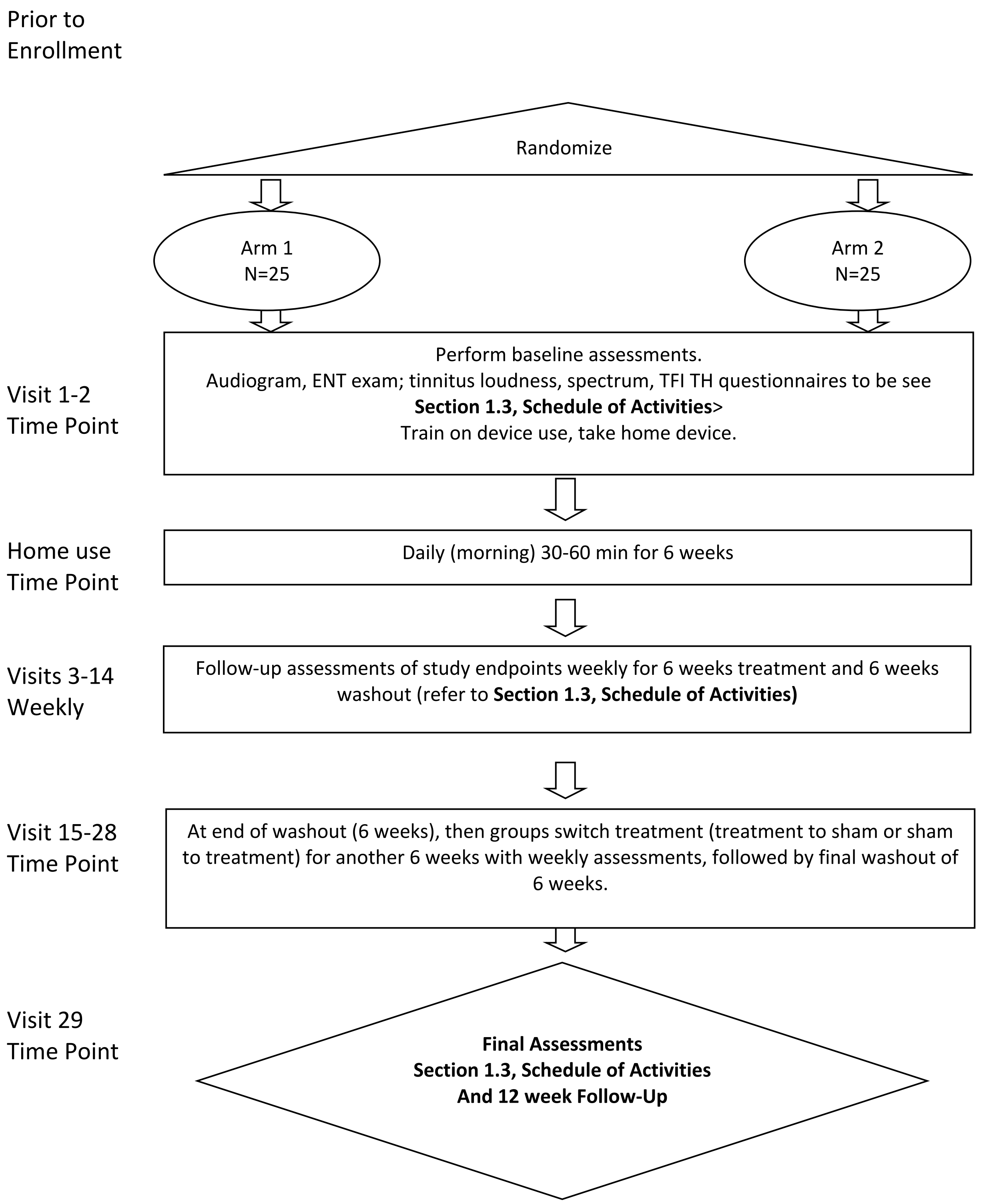
Prior to Enrollment
Randomize
Arm 1
N=25
Arm 2
N=25
Visit 1-2 Time Point
Perform baseline assessments.
Audiogram, ENT exam; tinnitus loudness, spectrum, TFI TH questionnaires to be see
Section 1.3, Schedule of Activities>
Train on device use, take home device.
Home use Time Point
Daily (morning) 30-60 min for 6 weeks
Visits 3-14 Weekly
Follow-up assessments of study endpoints weekly for 6 weeks treatment and 6 weeks washout (refer to Section 1.3, Schedule of Activities)
Visit 15-28 Time Point
At end of washout (6 weeks), then groups switch treatment (treatment to sham or sham to treatment) for another 6 weeks with weekly assessments, followed by final washout of 6 weeks.
Visit 29 Time Point
Final Assessments
Section 1.3, Schedule of Activities
And 12 week Follow-Up

1.3 SCHEDULE OF ACTIVITIES (SOA)

period		Arm 1		End Period 1	Washout	Arm 2		End Period 2	Washout	EOS	Post-study monitoring
visit	Screening	Baseline 1	End of weeks 1-5	End of week 6	End of week 7- 11	End of week 12/Baseline 2	End of weeks 13-17	End of week 18	End of week 19- 23	End of week 24	Weeks 28, 32, 36
day		Day 0	Days 7, 14, 21, 28, 35	Day 42	Days 49- 84	Day 91	Days 98, 105, 112, 119, 126	Day 133	Days 140- 168	175	252
window			'+ or - 3 days, but 5 days between weekly visits	'+ or - 3 days, but 5 days between weekly visits	'+ or - 3 days, but 5 days between weekly visits	'+ or - 3 days, but 5 days between weekly visits	'+ or - 3 days, but 5 days between weekly visits	'+ or - 3 days, but 5 days between weekly visits	'+ or - 3 days, but 5 days between weekly visits	'+ or - 3 days, but 5 days between weekly visits	'+ or - 3 days but 5 days between weekly visits
Informed Consent	x										
Medical History	x										
Tinnitus Maneuver Checklist	x			x		x		x		x	
Audiogram	x	x		x		x		x		x	
TFI	x	x	x	x	x	x	x	x	x	x	x
THI		x	x	x	x	x	x	x	x	x	
TinnTester		x	x	x	x	x	x	x	x	x	
THS		x		x		x		x		x	
MML		x		x		x		x		x	

2 INTRODUCTION

2.1 STUDY RATIONALE

Fifty million people in the United States suffer from tinnitus. Tinnitus is the number one service-connected disability for returning veterans from Iraq and Afghanistan. Tinnitus is most often the result of extreme noise exposure from either a single impulse noise or the accumulation of noise exposure; head and neck injury is the second leading complaint. In fact, lumbosacral or cervical strain account for 23% of service-connected disabilities for Iraq and Afghanistan veterans as of July 2009. In addition to factors that cause hearing loss, such as noise over-exposure, tinnitus can occur as a result of temporal-mandibular joint disorder, or somatic insults, including lumbosacral or cervical strain. Tinnitus is on the rise in young people under the age of 20 because of the increased use of high-volume personal music players.

The majority of tinnitus patients can modify their tinnitus by manipulating regions of the head and neck, such as in jaw clenching while many can attribute their tinnitus onset to a somatic insult in the head and neck region (Levine, 1999a, Levine, 1999b, Abel and Levine, 2004). Somatic manipulations of the head and neck can modulate or induce a tinnitus percept, even in people who do not have tinnitus. Some examples of this include clenching of the jaw or neck muscles. These maneuvers can modulate the tinnitus percept in up to 80% of subjects while light cutaneous pressure over the right scapula can induce a clicking tinnitus percept. Thus, while tinnitus has long been considered an exclusive disorder of the auditory system, accumulating evidence suggests that it is an expression of neural plasticity encompassing reactions of multisensory neurons to changes in the neural signaling environment due to changes in auditory input.

2.2 BACKGROUND

The connections of these somatosensory neurons to the first auditory brainstem nucleus, the cochlear nucleus (CN), suggests that tinnitus may be triggered by an alteration of firing rates in

CN neurons by abnormal activity in somatosensory neurons innervating the head and neck, especially after noise damage (Shore et al., 2008) as well as head and neck trauma. Indeed, increased spontaneous and sound-driven firing rates in the dorsal cochlear nucleus (DCN) have been shown to be physiological correlates of tinnitus (Brozoski et al., 2002, Shore et al., 2008, Dehmel et al., 2012b). Our work over the past several years has provided strong support for somatosensory neurons from the head and neck region being involved in the generation and maintenance of tinnitus (Zeng et al., 2009, Dehmel et al., 2012b, Zeng et al., 2012).

Preclinical work in our lab has shown that tinnitus is generated by neurons in a brainstem station, the dorsal cochlear nucleus. Here, principle neurons become hyperactive after tinnitus induction in an animal model. One important mechanism that regulates neuronal firing properties is spike-timing-dependent plasticity (STDP), the up- or down-regulation of synaptic strength based on the order and timing of pre- and post-synaptic activity. Spike-timing-dependent plasticity has been shown in the parallel fiber portion of the dorsal CN (DCN) circuit *in vitro* (Tzounopoulos *et al.*, 2004) and has been confirmed *in vivo* in our laboratory by demonstrating stimulus-timing dependent synaptic plasticity. **Stimulus timing dependent plasticity (StDP)** is the macroscopic correlate of STDP and has been shown to be a mechanism underlying persistent or lasting bimodal integration (Koehler and Shore, 2013a). This finding could have important, far-reaching implications for treating tinnitus, such as auditory-somatosensory training and with surface or deep brain stimulation, both of which would be more effective if operating through synaptic plasticity.

Thus, here we utilize STDP to instigate long term suppression in DCN neurons. Our studies have shown that particular sequences and intervals between somatosensory and auditory stimuli produce either enhancement or suppression (Koehler and Shore, 2013b). In our previous studies in animals we replicated this result using trans-cutaneous stimulation (Martel et al., 2014). The tonal quality of tinnitus suggests that hyperactive neurons are located in tonotopic regions similar to the tinnitus frequency. Thus, in order to treat tinnitus, hyperactive neurons in CN regions tonotopically similar to the tinnitus frequency should be suppressed. This should reduce or eliminate the phantom perception of sound. To ensure that this suppression is long-term, STDP based suppression in those neurons will be accomplished by combining frequency-specific auditory stimuli (i.e. close to the tinnitus frequency) with somatosensory stimuli with precise timing intervals. For more complex tinnitus, more complex auditory stimuli will be used to suppress firing rates across multiple regions. Somatosensory stimulation is achieved by placing electrodes on various skin sites in the head and neck region, which contain the neurons that send their axons to the CN.

The degree of hyperactivity and tinnitus can be modulated though auditory-somatosensory (bimodal) stimulation with specially-timed intervals that cause long term depression in the

tinnitus-initiating neurons, and thereby reduce tinnitus in the animal model. Remarkably the same stimulus protocol can reduce tinnitus loudness and life impact in human adults, as demonstrated in the stage 1 clinical trial which was completed last year (Marks, Martel et al. 2018). This stage 2 trial will replicate the previous trial but using more subjects, adding outcome measures, and increasing treatment and washout period durations.

2.3 RISK/BENEFIT ASSESSMENT

2.3.1 KNOWN POTENTIAL RISKS

The known or expected risks are:

Audiometric testing and tinnitus matching is performed routinely in audiology centers with no associated risks.

The device was custom manufactured for this study. It generates sound and somatosensory stimulation. There are software and hardware limiters in place to ensure the device never outputs any unsafe somatosensory or sound stimulation. We will also make individualized adjustments in the lab to ensure both the sound and somatosensory stimulation are also at comfortable levels for you. Because of the limits in place, you will be unable to make unsafe adjustments to the sound and somatosensory stimulation. This study is to test the effectiveness of the device.

All research has the potential risk of confidentiality breach. The investigators conducting the research have put the following mechanisms in place to protect confidentiality: all test results are de-identified. Information is also stored in password protected encrypted files or locked in a cabinet accessible only by the investigators.

Based on the study protocol and stimuli used, it is not anticipated that subjects would experience different or more adverse risks when tested in the treatment vs. sham arms of the study. The sham treatment will consist of stimuli for which there is evidence from animal studies to suggest that it will not cause improvement or worsening of their tinnitus and will have no other side effects.

As with any research study, there may be additional risks that are unknown or unexpected. While unlikely, it is possible that tinnitus may become louder or more noticeable during the trial.

2.3.2 KNOWN POTENTIAL BENEFITS

There may be no benefit to participants as a results of using the device. Alternatively, subjects may experience a decrease in tinnitus loudness or may be less bothered by their tinnitus after participating in the treatment arm of the study.

2.3.3 ASSESSMENT OF POTENTIAL RISKS AND BENEFITS

The study proposes to use a completely non-invasive device in order to alleviate symptoms of a condition (i.e., tinnitus) that affects millions of people worldwide. There is currently no consistent, effective treatment for tinnitus. An initial phase of this study (double-blinded crossover) demonstrated that when undergoing the active treatment, on average, participants (N=20) experienced a statistically significant reduction in their tinnitus loudness. Additionally, they reported their tinnitus to be less bothersome. Therefore, the benefits far outweigh the risks.

The funding institution has deemed this clinical trial as “greater than minimal risk” because it does include a placebo arm. Please refer to further documentation from the program officer (email) indicating this risk level following determination of NIMH.

3 OBJECTIVES AND ENDPOINTS

3.1 PRIMARY MEASURES

1) Tinnitus loudness: Measurements by the TinnTester software (McMaster University, supplied by Dr. Larry Roberts) are completed by interactive computer administered in a soundproof booth. To assess loudness in this procedure, subjects adjust the level of each of 11 sounds differing in center frequency to match the level of their tinnitus. This gives a measure of loudness in sound pressure level (SPL) for each subject (loudness matching method). The subjects then rate each sound for the similarity of its frequency to the frequency of their tinnitus. This gives the tinnitus frequency spectrum for each subject. Data are recorded automatically by the software. This software package has been used extensively by the tinnitus research community and is published (Roberts et al, 2008). The TinnTester sounds will be delivered by the devices through calibrated earphones. The devices have hardware limits on the sound pressure levels so that they are presented at safe levels (lower than 60 dB SL (above threshold).

2) Tinnitus Functional Index (TFI): The 25 item version of the TFI determines severity of tinnitus on a 100 point numeric scale (scores above 17 indicate the patient's tinnitus is a problem) based on patients' responses to 25 questions regarding the impact their tinnitus has on their emotional, social, and mental well-being. A thirteen-point reduction in score is considered clinically significant, indicating a noticeable improvement in tinnitus. This version has recently been validated for the test-retest reliability and internal consistency in New Zealand and the UK.

3) Tinnitus Handicap Inventory (THI): A 25 item questionnaire which assesses subjective impacts of tinnitus related to quality of life on a 100 point numeric scale. Scores 17 points or higher are considered bothersome tinnitus. We are including this measure as several other studies use it so it is a point of comparison.

3.2 SECONDARY MEASURES

1) Tinnitus bandwidth/spectrum: Near its start the TinnTester software asks subjects to indicate which of three sounds best resembles their tinnitus; 5 kHz pure tone ("tonal" tinnitus), a narrow band noise centered at 5 kHz (±5% of CF, BPN5 masker, "ringing" tinnitus), or a wider band of noise centered at 5 kHz (±15% of CF, BPN15 masker, "hissing" tinnitus). We will relate treatment outcomes to tinnitus bandwidth by these measurements. We will also be able to determine whether the treatment alters bandwidth.

2) Minimum Masking Level (MML): Subjects adjust the level of the 5 kHz BPN15 sound until they can no longer hear their tinnitus. MML may prove sensitive to the bimodal treatment procedure. MML should be reduced, if tinnitus assessed by loudness matching is also reduced. However, even when tinnitus loudness is reduced, there are subjects for whom tinnitus cannot be masked.

3) Tinnitus Hearing Survey: A brief nine question survey which determines self-perceived handicap due to tinnitus, hearing loss, and hyperacusis. This questionnaire differs from the two primary questionnaires in that it qualifies any perceived handicap due to hearing loss and/or sound sensitivity (hyperacusis).

OBJECTIVES	*ENDPOINTS*	*JUSTIFICATION FOR ENDPOINTS*
Primary		
1) Objective Tinnitus loudness (TinnTester)<br><br>*2) Tinnitus Functional Index*<br><br>*3) Tinnitus Handicap Index*	*1) End of 1st arm*<br>*2) End of first washout*<br>*3) End of 2nd arm*<br>*4) End of second washout*<br><br>*1) End of 1st arm*<br>*2) End of first washout*<br>*3) End of 2nd arm*<br>*4) End of second washout*<br><br>*1) End of 1st arm*<br>*2) End of first washout*<br>*3) End of 2nd arm*<br>*4) End of second washout*	*To determine how use of device affects objective tinnitus loudness before and after the treatment and sham arms of the study.*<br><br>*To determine how use of device affects the tinnitus and quality of life before and after the treatment and sham arms of the study*<br><br>*To determine how use of device affects the tinnitus and quality of life before and after the treatment and sham arms of the study*
Secondary		
1) Tinnitus bandwidth/spectrum (TinnTester)<br><br>*2) Minimum Masking Level (MML)*	*1) End of 1st arm*<br>*2) End of first washout*<br>*3) End of 2nd arm*<br>*4) End of second washout*<br><br>*1) End of 1st arm*<br>*2) End of first washout*<br>*3) End of 2nd arm*	*To determine how use of the device may or may not change the perceived frequency (spectra) of tinnitus following the treatment or sham arms.*<br><br>*To determine if the MML for a 5kHz tone*

OBJECTIVES	*ENDPOINTS*	*JUSTIFICATION FOR ENDPOINTS*
	4) End of second washout	*is reduced in the active treatment arm*
3) Tinnitus Hearing Survey	*1) End of 1st arm*<br>*2) End of first washout*<br>*3) End of 2nd arm*<br>*4) End of second washout*	*To determine if the active treatment has an effect or interaction with perceived handicap due to hearing loss and sound level tolerance*

4 STUDY DESIGN

4.1 OVERALL DESIGN

Aims/Hypotheses:

Note: Aim 1 of the grant is to be performed in ANIMAL subjects only. However, Aim 2 of the grant is to be carried out in human subjects and therefore the design of Aim 2 is somewhat dependent on the results of Aim 1. For information purposes only, both are described below:

Specific Aim 1: Determine precise parameters/dosages for modulating multisensory plasticity in fusiform cells (FCs) and cartwheel cells (CWCs) in normal and pathological (tinnitus) circuitry in guinea pigs. *Hypothesis 1: Presenting bimodal AN-SS stimuli with optimal parameters to guinea pigs with tinnitus will reverse pathological StDP in FCs and CWCs.*

Specific Aim 2: Test parameters/dosages determined in Specific Aim 1, for modulation of multisensory plasticity in human subjects with changes in tinnitus severity as the outcome measure. Hypothesis 2: AN-SS stimuli with optimal dosage/parameters (based on the results of Specific Aim 1), will effectively modulate DCN circuitry to reduce tinnitus in humans. Experiment 2a: Using a double-blind cross-over model, we will use a bimodal AN-SS paradigm with optimal stimulus parameters based on the results of Specific Aim 1 to assess changes in subjects' tinnitus perception. Modulations in tinnitus perception will be assessed using objective loudness matching and subjective measures (standard questionnaires) in response to active and sham bimodal stimuli. Neural circuitry changes will be assessed by examining changes in the severity of subjects' tinnitus. It is anticipated that chosen bimodal intervals and durations of treatment (dose) will reduce the abnormal percepts. Experiment 2b: Subjects will

be monitored during a washout period for a return to baseline to determine durability of effect. To assess dose-response of the bimodal modulation, the length of bimodal stimulation treatment will be varied and effects and duration of effect on tinnitus will be assessed as above

Phase: Phase I/II

Type of design: Double-blinded, cross-over design. There will be two treatment arms (treatment and sham). All subjects will participate in both arms of the study. This is a single-site study. Total duration of the study for each subject will be 36 weeks.

4.2 SCIENTIFIC RATIONALE FOR STUDY DESIGN

Previous studies examining efficacy of various tinnitus treatments have shown a strong placebo effect (Dobie 1999). Therefore, is important to include a placebo arm in order to truly evaluate the efficacy of the device and treatment. It is also important that both the subject and the administrator (all study team members) are also blinded to the enrollment and assignment of the subjects for the same reasons. The placebo stimulus is an auditory stimulus which is benign and shown in the first trial to have no effect (Marks et al, Science Translational Medicine, January, 2018, HUM00088432).

4.3 JUSTIFICATION FOR DOSE

The justification for dosage is based on Aim 1 of the project (preclinical experiments) as stated above.

4.4 END OF STUDY DEFINITION

A participant is considered to have completed the study if he or she has completed all phases of the study including the last visit or the last scheduled procedure shown in the Schedule of Activities (SoA), Section 1.3.

5 STUDY POPULATION

5.1 INCLUSION CRITERIA

In order to be eligible to participate in this study, an individual must meet all of the following criteria:

1. Must be 18 years of age or older.

2. A score of >17 points on the Tinnitus Functional Index, as measured at the screening visit.
3. Must report constant, subjective, preferably unilateral tinnitus without any active external or middle ear pathology.
4. No greater than a moderate hearing loss at the tinnitus frequencies (≤55 dB HL) and no greater than moderate hearing loss (≤50 dB HL) from 125 – 6000 Hz .
5. Must be able to modulate their tinnitus with a somatic maneuver
6. Preferably onset of tinnitus less than one year ago, but for at least 6 months. Tinnitus should be bothersome.
7. Absence of retrocochlear pathology/ VIIIth nerve lesion
8. No participation in a tinnitus treatment regimen within the past six months and no participation in Stage 1 trial.
9. Resides within 100 miles of study site.

5.2 EXCLUSION CRITERIA

*A*n individual who meets any of the following criteria will be excluded from participation in this study:

1. Anyone not meeting the above inclusion criteria, and in addition:
2. Diagnosis of Meniere's disease
3. Diagnosis of Semicircular Canal Dehiscence.
4. Unilateral or bilateral cochlear implant recipients
5. Diagnosis of acoustic neuroma
6. Evidence of retrocochlear disease
7. Patients with any indwelling electronic stimulation devices
8. Current of previous use of any acoustic hearing aid or over-the-counter personal sound amplification product (PSAP) in the last 6 months.
9. Current or former use of any of the following medications within the past 6 months: high dose aspirin (>325mg/day), high dose NSAIDs (ibuprofen, Motrin, Celebrex) (>800mg/day), narcotics (any opioids), lithium, clonazepam, oxazepam, cholinergic medications (anti-dementia medications), anti-depressant medications (serotonin specific reuptake

inhibitors; SSRIs, tri-cyclic anti-depressants; TCAs); anti seizure/convulsant medications (Depakote), anti-psychotic medications (Haldol, Seroquel), Ototoxic medications, other chemotherapy agents, central nervous system stimulants, and benzodiazepines.

10. Current diagnoses of any of the following: obsessive compulsive disorder (OCD), schizophrenia, bipolar disorder, extreme generalized anxiety disorder, drug/alcohol dependence.
11. Pregnant or Nursing.

5.3 LIFESTYLE CONSIDERATIONS

During this study, participants are asked to:

- Refrain from participation in any other tinnitus treatment
- Report any changes in health status
- Report any changes in prescribed or over-the-counter medications

5.4 SCREEN FAILURES

Screen failures are defined as participants who consent to participate in the clinical trial but are not subsequently randomly assigned to the study intervention or entered in the study. A minimal set of screen failure information is required to ensure transparent reporting of screen failure participants, to meet the Consolidated Standards of Reporting Trials (CONSORT) publishing requirements and to respond to queries from regulatory authorities. Minimal information includes demography, screen failure details, eligibility criteria, and any serious adverse event (SAE).

Individuals who do not meet the criteria for participation in this trial (screen failure) because of a recent participation on another tinnitus treatment regimen may be rescreened once the observed period of time as passed as stated in the protocol. **Rescreened participants should be assigned the same participant number as for the initial screening.**

5.5 STRATEGIES FOR RECRUITMENT AND RETENTION

General procedures:

Study team members will recruit patients from patient populations seen in the otology and audiology clinics at UMHS, as well as those who have contacted Dr. Shore or other study team members to report having tinnitus. Once they have contacted a study team member, they will receive an automatic reply which will be sent using the Mail Chimp email automation platform. Please see additional documentation for Mail Chimp platform security information (Attachment A) and an email script (Attachment B) that will be sent to potential participants. Within the email script, potential participants will be directed to the clinical study website which will appear on the umhealthresearch.org website. Participants will be asked to create an account

and log in, and then they will have the ability to contact the study team through the website if they are interested in participating. Please see Attachment C for content that will be posted on umhealthresearch.org. Once a potential participant has contacted the study team using the umhealthresearch.org website, he/she will be contacted by a member of the study team via phone, email, or secure messaging via the umhealthresearch.org website in order to confirm that they meet the inclusion and exclusion criteria. Please see additional documentation for phone and/or email script (Attachment D). Once such information is confirmed, they will be scheduled for an initial visit to the lab at which time consenting and candidacy evaluations will be performed.

Subjects may also sign the informed consent through the SignNow application. Subjects will meet with a staff member through HIPAA compliant Zoom videocall platform where the staff member will review the informed consent with the subject and answer any questions the subject may have prior to signing the document.

The patient will have the opportunity to ask questions at all times throughout the recruitment process. All questions or concerns will be addressed by a study team member. Through purposeful sampling, we will attempt to include a diverse set of respondents based on ethnic, socioeconomic, and educational backgrounds.

Target study sample size: We will enroll 400 subjects in order to assign up to 100 subjects to 2 treatment arms. We anticipate testing 34 subjects per year in order to meet this recruitment goal. There is one recruiting site (University of Michigan).

Subjects will be provided compensation for parking at each visit.

6 STUDY INTERVENTION

6.1 STUDY INTERVENTION(S) ADMINISTRATION

6.1.1 STUDY INTERVENTION DESCRIPTION

Describe the study intervention(s) and control product. Product information can usually be obtained from the:

- The investigational devices for providing the stimulation protocols have been designed by members of the Shore Lab and fabricated by In2being, LLC. The device provides acoustic and electrical stimulation at levels deemed safe by OSHA, ISO and ANSI.
- The device is labeled “for investigational use only”
- Final labeling for a marketed device will be obtained after FDA final review after completion of the trial. In2being, LLC will incorporate any changes to the labeling that are suggested.
- A pre-submission has been reviewed already by the FDA and they were in agreement that this device would be de novo, non-significant risk.

In addition:

- FDA and the IRB consider our device class 2 de novo, thus we do not require an IDE to run the clinical trial, stage 2.
- The study intervention is not yet commercially available. Since the stage 1 clinical trial that has been completed, we are hiring In2being to upgrade the devices so that the next generation is FDA compliant.
- *If conducting a study with a device, the following information should be included:*
    - Device size(s): Less than 3”/2”/7”
    - Device model(s): Gen 2
    - Description of each component:
        - CPU
        - SD card for data storage
        - power supply
        - rechargeable lithium ion batteries
        - electrical stimulator
        - sound stimulator
        - earphones
        - stimulating pads
    - Device settings and programming:
        - See Figure 1 for all device tunable parameters
        - Auditory SPL below 95 dB SLP
        - Current less than 3 mA (see Figure 1)
        - Misapplication of electrodes is detected by the device as shown by red light.
        - Device has only two settings (on/off), limiting subject errors.
    - Duration of stimulation (30-60 min/day).
    - Once per day
    - Current levels with known potentiometer values and Maximum SPL are shown in Fig. 2

- **Figure 1: Device Tunable Parameters.** Each indicated parameter is regulated by the device. Current level, audio intensity and frequency content are altered on an individualized basis for the treatment; all other parameters remain fixed.
-

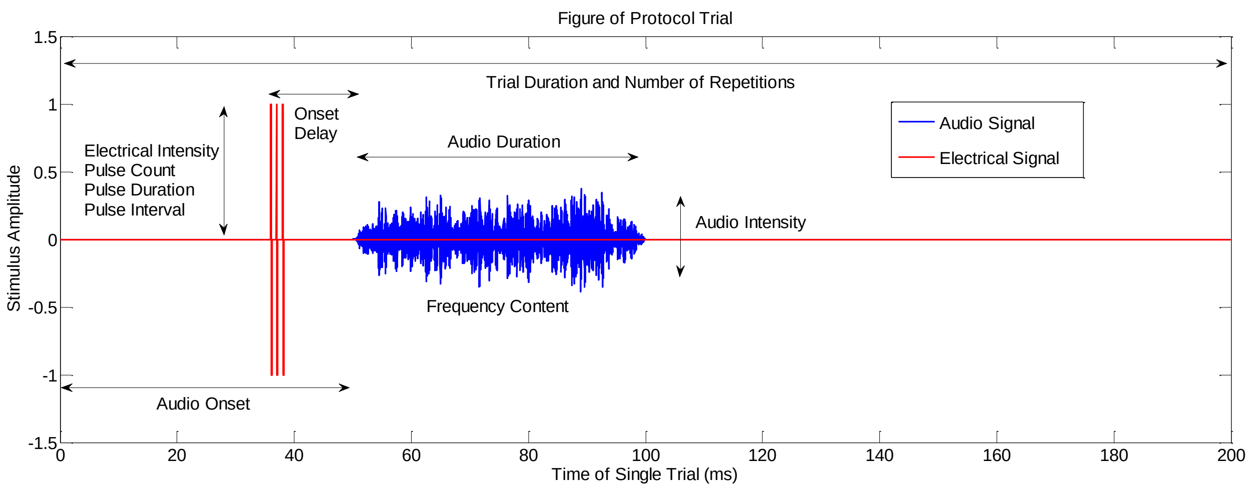
Figure of Protocol Trial
Trial Duration and Number of Repetitions
Onset
Delay
Audio Duration
Audio Signal
Electrical Signal
Electrical Intensity
Pulse Count
Pulse Duration
Pulse Interval
Audio Intensity
Frequency Content
Audio Onset
Stimulus Amplitude
Time of Single Trial (ms)
1.5
1
0.5
0
-0.5
-1
-1.5
0
20
40
60
80
100
120
140
160
180
200

-

- **Figure 2. Calibration Data.** Stimuli are calibrated prior to device use. **A)** Each device features an internal self-calibration for the electrical stimulator. This check is run every trial to ensure appropriate treatment stimulation. This feature also checks for open and short circuits, which indicate improper electrode placement. For a given potentiometer value (x-axis), the device output current (right y-axis) and regulated voltage (left y-axis) are shown. **B)** Device ear phones are matched to a device and calibrated using a standard microphone. Dashed line shows earphone output without limiter installed, solid with limiter.

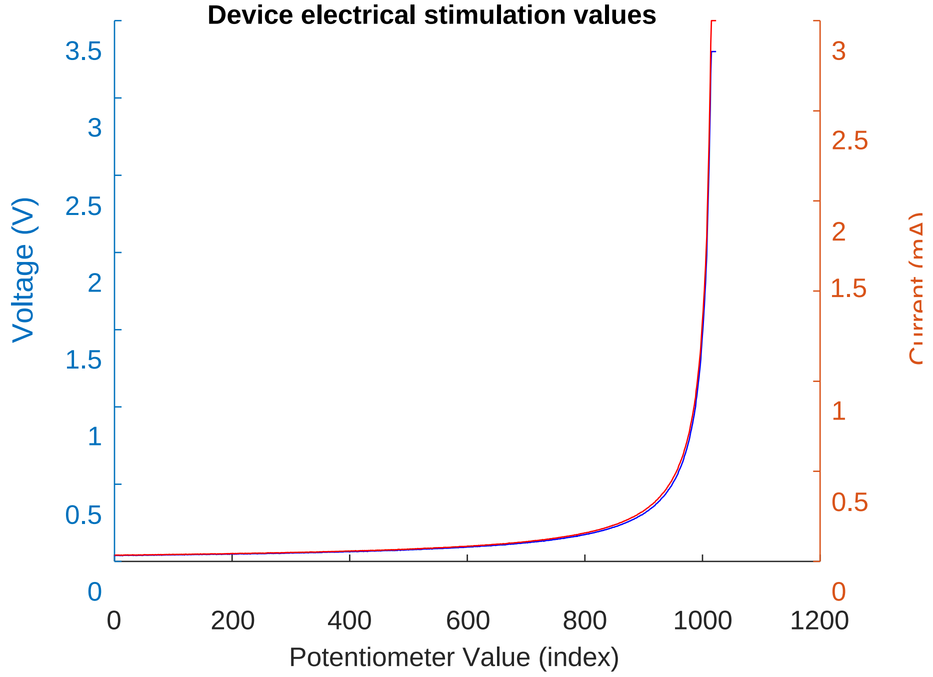

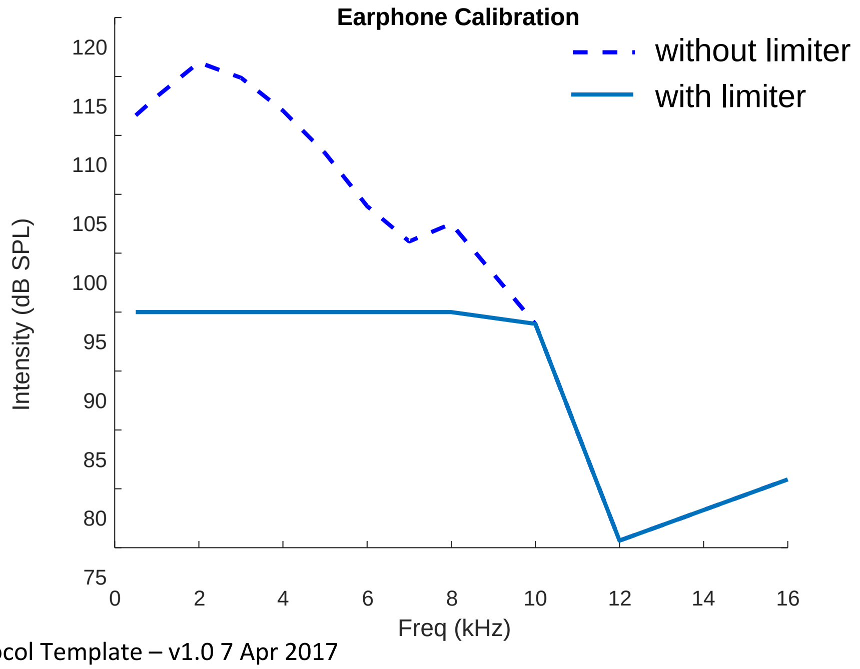

6.1.2 DOSING AND ADMINISTRATION

Each subject will have their tinnitus spectrum assessed with the Tinn Tester protocol (L. Roberts et al, 2008). This spectrum is digitized and programmed into the device as the signal to be delivered to the subject. The amplitude is limited to 95 dB SPL and is delivered together with an electrical stimulus delivered a short time after the auditory stimulus (as determined by the preclinical studies). This stimulus is presented by the device when the subject turns on the device. The device limits the time of presentation to 30-60 min per day.

This protocol is presented once a day for 30-60 minutes for 6 weeks. After that there is a washout period of 6 weeks in which no device is used. Subjects are tested once a week to assess the primary and secondary outcomes and when baseline is achieved. The second arm of the study begins for another 6 weeks. A second washout of 6 weeks occurs, end of study visit, and 12 weeks of follow up, after which the subject participation is ended. Completion of Arm 1 and Arm 2 do not necessarily have to be consecutive, and we will allow for 12 weeks in between in order to provide more flexibility for subjects. The dose will not change over the course of the study. Maximum duration of enrollment is 36 weeks.

The subjects will be trained on how to use the devices and place the earphones and electrode pads. Once they have been effectively trained, subjects will take the devices home to use for the 30-60 min/day periods. Once a week the primary and secondary outcomes will be tested along with any review or questions that the subjects need to be answered. Subjects are asked to use the device at the same time every day but If subjects miss a treatment they are instructed to use it at another time rather than miss that treatment.

6.2 PREPARATION/HANDLING/STORAGE/ACCOUNTABILITY

6.2.1 ACQUISITION AND ACCOUNTABILITY

In2being will provide a unique identifier for each device. The study team members will program the devices based on individuals' audiograms and tinnitus spectra and will show the subjects how to use the devices to implement the treatments. Devices will be returned to study team members following subject participation. Devices will be reprogrammed for subsequent subjects.

6.2.2 FORMULATION, APPEARANCE, PACKAGING, AND LABELING

See Figure 3.

Figure 3. Generation 1 Device Pictures. Generation 2 devices, to be tested in this study, will not be larger than the current designs, and will feature upgrades to ensure FDA approval.

Device dimensions: less than 2” x 5 “ x 8”

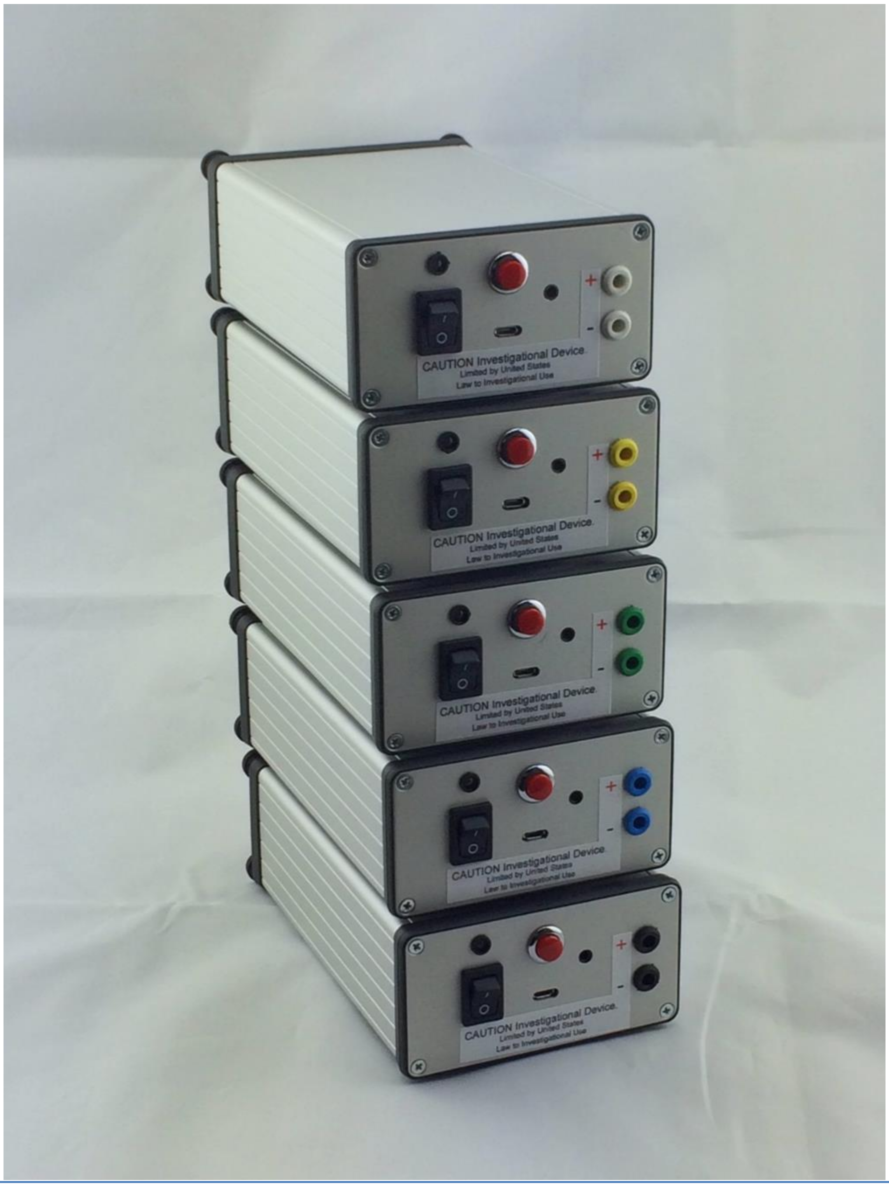

6.2.3 PRODUCT STORAGE AND STABILITY

Kept at room temperature when not in use. Subjects will be supplied with sufficient electrode pads and gel and earphone tips to ensure use of new pads and closed acoustic system with each treatment.

6.2.4 PREPARATION

Subjects are required to gently wipe area for electrode contact (neck skin or face skin) with alcohol-based wipes prior to electrode placement.

6.3 MEASURES TO MINIMIZE BIAS: RANDOMIZATION AND BLINDING

Subjects are randomly assigned to group 1 (active first, then sham) or group 2 (sham first, then active) using Matlab's *randsample* command with a predetermined, fixed, secret seed value. *Randsample* generates random numbers using the Mersenne twister algorithm. We will enroll subjects in a 1:1 ratio to create a balanced study design. To ensure blinding, group assignment is generated *in silico* and assignment programmed into each device, without the intervention of a study member. This parameter is not made accessible to study members without password authorization. Devices are encrypted to prevent study team members or subjects from accessing device data in an unauthorized manner.

As the randomization list is generated algorithmically, the code can be reproduced with knowledge of the secret seed value. Unblinding may occur at the end of the study or in the case of an SAE related to the study (worsening of tinnitus, worsening of hearing threshold). Study investigators that do not have contact with subjects may unblind. Intentional and unintentional unblinding must be reported to the ~~principle~~principal investigator.

Imperfect blinding could arise from the presence of the electrical stimulation present during active treatment. To avoid unblinding, subjects will be told that they may or may not feel electrical stimulation during both phases of the study. The previous study suggested that subjects tend to acclimate to the presence or lack of electrical stimulation during a day's treatment. Subjects will be discouraged from divulging whether they can feel the electrical stimulus.

No investigators are allowed to remain unblinded for the duration of the study. All analyses that could require unblinding (SAEs, worsening of a subject's tinnitus/hearing) will be identified by software, and the investigators notified by automated communication methods.

6.4 STUDY INTERVENTION COMPLIANCE

The devices are electronic monitoring compliant – successful daily use is registered, also detects and records open circuits or failures. Primary and secondary outcomes are measured weekly and recorded by study team members electronically on secure databases.

6.5 CONCOMITANT THERAPY

NA

6.5.1 RESCUE MEDICINE

NA

7 STUDY INTERVENTION DISCONTINUATION AND PARTICIPANT DISCONTINUATION/WITHDRAWAL

7.1 DISCONTINUATION OF STUDY INTERVENTION

This study uses a non-invasive device. We will use strictly calibrated instruments to ensure that both acoustic and electrical stimulation are being delivered at safe levels for each subject. However, specific stopping rules will be identified for each subject to ensure patient safety. For each subject, data for the TFI, THI, and TinnTester will be examined on a weekly basis (following weekly testing for each subject) for tinnitus assessments. Additionally, audiometric criteria will be assessed at least every 6 weeks (please see schedule of events) to monitor hearing status. Audiometric criteria will be evaluated during the interim if a subject reports concern for a change in hearing ability. This metric will be monitored by the ISM. If tinnitus is found to statistically, significantly increase according to aggregated mean baseline, then the subject will not continue in the study regardless of which arm (active or sham) in which the subject is enrolled. Furthermore, if the subject subjectively reports increase in tinnitus or worsening of hearing, without a significant change in any of the subjective or objective measures, and he/she wishes to discontinue participation in the study then he/she will withdraw from the study.

A subject’s participation in the study will be discontinued following **both** 1) an increase of 16 or more points in TFI, **and** 2) an average increase of 12 dB in TinnTester compared to baseline, for two consecutive weekly measures. Both measures are our primary outcome measures. Using both criteria for determining discontinuation is critical as the tinntester measures tinnitus loudness, which reflects the circuitry changes identified in our preclincal studies. TFI is also important for the human study as it gauges the subject’s subjective response to their tinnitus. However, TFI can also reflect outside life stressors unrelated to the treatment and could result in a subject's discontinuation for reasons unrelated to the treatment.

7.2 PARTICIPANT DISCONTINUATION/WITHDRAWAL FROM THE STUDY

- Pregnancy
- Significant study intervention non-compliance
- If any clinical adverse event (AE), laboratory abnormality, or other medical condition or situation occurs such that continued participation in the study would not be in the best interest of the participant
- If the participant meets an exclusion criterion (either newly developed or not previously recognized) that precludes further study participation
- Participant becomes unable to participate in the study on a weekly basis.

The reason for participant discontinuation or withdrawal from the study will be recorded on the Case Report Form (CRF). Subjects who sign the informed consent form and are randomized but do not receive the study intervention may be replaced. Subjects who sign the informed consent form, and are randomized and receive the study intervention, and subsequently withdraw, or are withdrawn or discontinued from the study, will be replaced.

7.3 LOST TO FOLLOW-UP

A participant will become ineligible to continue in the study if he/she misses more than one visit for each of the following 6-week time periods: Arm 1 treatment, Arm 1 washout, Arm 2 treatment, Arm 2 washout. It is not considered a protocol deviation if a subject misses only one visit in any 6-week time period. A participant will be considered lost to follow-up if he or she fails to return for 2 consecutive scheduled visits and is unable to be contacted by the study site staff.

The following actions must be taken if a participant fails to return to the clinic for a required study visit:

- The site will attempt to contact the participant and reschedule the missed visit within 1 week and counsel the participant on the importance of maintaining the assigned visit schedule and ascertain if the participant wishes to and/or should continue in the study.
- Before a participant is deemed lost to follow-up, the investigator or designee will make every effort to regain contact with the participant (where possible, 3 telephone calls, emails or portal messages).
- Should the participant continue to be unreachable, he or she will be considered to have withdrawn from the study with a primary reason of lost to follow-up. If participant refuses to return device, the Office of General Council and/or the UM Police department will be notified of device theft, pursuant to Mich. Comp. § 750.356(3), § 750.356a(1), “Felony Theft in Michigan”.

8 STUDY ASSESSMENTS AND PROCEDURES

8.1 EFFICACY ASSESSMENTS

Study Procedures:

General overview: All subjects will complete consenting and then a candidacy evaluation to determine if they meet study criteria. Once they meet criteria, they will be scheduled for a baseline re-assessment. Following the baseline assessment, they will be randomly assigned to a study arm (treatment or sham) and then training on how to use the device before taking it home. They will take home the device on the same day they complete the baseline assessment. Follow up assessments will take place weekly, and assessments completed at the weekly session are noted below. Additionally, some measures will only be completed at the beginning or end of the active treatment or sham conditions and following washout of active or sham treatment.

Specific procedures:

1. Audiologic case history:
    a. Will be administered to each enrolled subject at the initial appointment in the study (candidacy evaluation) following completion of the consent form. This questionnaire includes basic information regarding the patient's audiologic and medical history. This will be administered by the study team audiologist or other trained technician. This is not repeated at any other point in the study unless the patient reports a change in audiologic history.
2. Otologic examination and medical case history:
    a. Will be administered to each enrolled subject at the initial appointment in the study (candidacy evaluation) following completion of the consent form. This questionnaire includes basic information regarding the patient's otologic and medical history. This will be administered by a physician/otologist on the study team. This is not repeated at any other point in the study unless the patient reports a change in otologic or medical history.
    b. If an otologic examination warrants further medical evaluation or intervention (standard of care) then an appropriate recommendation will be made by a physician study team member. If a subject is required to seek further medical evaluation, he/she will not be able to participate in the trial until he/she has been properly evaluated and has provided test results or a letter from a physician indicating as such. These costs are not covered by the study.
    c. If mental health issues are identified during the screening process, the screening physician will refer the subject back to their primary care physician for further evaluation and/or follow-up.
3. Tinnitus modulation checklist
    a. Will be administered to each enrolled subject at the initial appointment in the study (candidacy evaluation) following completion of the consent form. This questionnaire involves a study team member asking the subject to perform a series of movements and then requiring that the subject subjectively report if a given movement caused a change in tinnitus. Only subjects who are able to modulate their tinnitus with one of the somatic maneuvers will be eligible to participate in the study. This will be assessed at the candidacy evaluation, and then according to the Schedule of Events (Section 1.3).
4. Audiometric assessment:
    a. A basic audiometric assessment will be performed at the initial appointment in the study (candidacy evaluation) following completion of the consent form and then according to the Schedule of Events (Section 1.3). The basic audiometric assessment follows standard clinic procedures in both ears. This includes a behavioral audiogram using air-conduction (250-8000 Hz) and bone conduction (250-4000 Hz) stimuli presented through calibrated headphones or bone conduction transducer, respectively. Speech detection thresholds will also be assessed to determine reliability. This data will be assessed to determine if a subject meets eligibility criteria for hearing loss. It will also be reassessed bi-weekly to determine if hearing thresholds worsen during each treatment arm as outlined in the stopping criteria.

5. TinnTester:
    a. Objective tinnitus loudness (TinnTester): This assessment is described in Section 3 of this document. It will be assessed at the baseline evaluation, and then according to the Schedule of Events (Section 1.3).
    b. Objective tinnitus bandwidth (TinnTester): This assessment is described in Section 3 of this document. It will be assessed at the baseline evaluation, and then according to the Schedule of Events (Section 1.3).
    c. Sound tolerance level (TinnTester): This assessment is described in Section 3 of this document. It will be assessed at the baseline evaluation, and then according to the Schedule of Events (Section 1.3).
6. Minimum Masking Level:
    a. This assessment is described in Section 3 of this document. It will be assessed at the baseline evaluation, and then according to the Schedule of Events (Section 1.3).
7. Tinnitus Function Index (TFI)
    a. This assessment is described in Section 3 of this document. It will be assessed at the candidacy evaluation, and then according to the Schedule of Events (Section 1.3).
8. Tinnitus Handicap Inventory (THI)
    a. This assessment is described in Section 3 of this document. It will be assessed at the baseline evaluation, and then according to the Schedule of Events (Section 1.3).
9. Tinnitus Hearing Survey (THS):
    a. This assessment is described in Section 3 of this document. It will be assessed at the baseline evaluation and then according to the Schedule of Events (Section 1.3)

8.2 SAFETY AND OTHER ASSESSMENTS

Subject participation will entail weekly visits to the lab for detailed assessments. The weekly visits to the lab will ensure that subjects are using the devices correctly and safely. The weekly visits also allow us to monitor changes in the tinnitus percept in response to device use or washout from the treatment. The regular assessments also provide us with the opportunity to detect any adverse events as soon as possible, should any occur.

Currently, there is no standard treatment for tinnitus that has been shown to be broadly effective at treating tinnitus, so participation in the study will not interfere with standard clinical intervention. Subjects will be informed during the consenting process that there are alternative methods of treatment such as sound therapy that they may find ~~beneficial,~~

~~and~~beneficial and referred to their primary care physician for referrals should they elect to pursue that form of treatment.

Regular assessments will also allow for swift intervention should a subject experience an adverse event or worsening of symptoms. We will stop the study should 10% of subjects show a persistent worsening in their symptoms. A subject's participation in the study will be discontinued following **both** 1) an increase of 16 or more points in TFI, **and** 2) an average increase of 12 dB in TinnTester compared to baseline, for two consecutive weekly measures. Both measures are our primary outcome measures. Using both criteria for determining discontinuation is critical as the ~~tinntester~~TinnTester measures tinnitus loudness, which reflects the circuitry changes identified in our ~~preclincal~~preclinical studies. TFI is also important for the human study as it gauges the subject's subjective response to their tinnitus. However, TFI can also reflect outside life stressors unrelated to the treatment and could result in a subject's discontinuation for reasons unrelated to the treatment. A subject's participation can also be discontinued even if the criteria above is not met, but the subject feels their tinnitus is worse.

This study does not require and will not use a Data Safety Monitoring Board (DSMB) because it is not a multi-site study. However, because it is a double-blinded, cross-over study we will implement and an Independent Safety Monitor (ISM) for data monitoring purposes. The ISM *will be clinical research monitor of the Michigan Institute for Clinical & Health Research (MICHR) who can provide independent monitoring of the human data. The ISM will be responsible for overseeing and monitoring the enrollment data, safety data and data integrity. The ISM will be unblinded to the intervention assignment for each subject. The ISM will be independent of the investigation. He/she will not be a collaborator, co-author, supervisor, mentor/mentee, research lab member, or member of the institutional department within the last three years. He/she will not have any professional or financial conflict of interest.*

The PI (Susan Shore, PhD) will comply with the NIMH Reportable Events policy. The intervention will continue for each subject so long as adverse events do not occur for that individual. The ISM will monitor data and review safety data. If adverse events related to the study (definitely related, probably related, or potentially related) do occur, they will be reported to the IRB within 14 days and will be reported to the PI immediately. All other adverse events will be reported annually at the time of IRB renewal. This study uses a non-invasive device. We will use strictly calibrated instruments to ensure that both acoustic and electrical stimulation are being delivered at safe levels for each subject. However, specific stopping rules will be identified for each subject to ensure patient safety. For each subject, data for the TFI, THI, TinnTester, and audiologic examinations will be examined on a weekly basis (following weekly testing for each subject) for tinnitus assessments to determine if either ~~measures~~measure indicate a statistically significant increase in tinnitus or decrease in hearing (in reference to the individual's baseline measure). This metric will be monitored by the ISM. If tinnitus is found to increase or hearing loss worsen, then the subject will not continue in the study regardless of which arm (active or sham) in which the subject is enrolled. Furthermore, if the subject subjectively reports increase in tinnitus or worsening of hearing, without a significant change in

any of the subjective or objective measures, and he/she wishes to discontinue participation in the study then he/she will withdraw from the study.

The study protocol and treatment device includes transmission of both acoustic and electric (transdermal) stimulation. For both stimulation types, stimuli are strictly controlled as to not cause harm or discomfort for the subject. For acoustic stimulation, the intensity is set at 30 dB SL (as determined by the subject's pure tone average), with each sound presented for 10 ms with 2 ms linear rise and fall times. This intensity level is expected to be within a comfortable listening range for each subject (based on clinical experience and previous research trial data). For the electric stimulation, the electrodes are positioned on the cheek overlying the trigeminal ganglion, the juncture of the temporomandibular joint or on the neck at overlying cervical nerves, C1 or C2, depending on which region modulates the tinnitus (based on the modulation maneuver checklist. Electrical stimulation levels are just below the threshold of muscular contraction (1-3 mA) as in the animal studies, limited by subject's comfort. This level of electric stimulation is typically one that initially results in a small, comfortable tinging at the stimulation site upon initial activation. However, based on our previous research trial subjects often report that electric stimulation cannot be detected within a few minutes of initial stimulation. This low level of electrical stimulation causes no harm to the subject. The sham protocol uses the same electrical and acoustic levels but the bimodal interval is wider than that used to induce STDP (>100 ms). Based on work in the animal, we expect that using wider bimodal intervals will cause no harm to the subject.
The subject will be consented by one of the study team members at the initial candidacy appointment. Consenting will take place in the Human lab on the 2nd floor of Med. Sci. I. at the University of Michigan, or online via HIPAA approved Zoom videoconferencing application. Patients will be seated in a comfortable chair in a semi-private room and be allowed to ask questions regarding the study. Tinnitus assessments will take place in a private, double-walled sound booth. Fitting of the device will take place in a semi-private laboratory room. Medical evaluations will be completed by Dr. Basura or Dr. Stucken in a private examination room at the Otology Clinic at the University of Michigan, in the Human Lab on the 2nd floor of Med. Sci. I, or online via HIPAA approved Zoom videoconferencing application. Any incidental findings will be reported to the subject and Dr. Basura or Dr. Stucken will make the appropriate medical referrals if necessary.

We will keep all subject information secure. All data will be collected and tracked electronically. Data will be stored, using a secure data network system supported by the Michigan Institute for Clinical and Health Research (MICHR). Subjects will communicate with research team members by phone or a secure web portal. Data will be coded, password protected and encrypted.

8.3 ADVERSE EVENTS AND SERIOUS ADVERSE EVENTS

8.3.1 DEFINITION OF ADVERSE EVENTS (AE)

Adverse event means any untoward medical occurrence associated with the use of an intervention in humans, ~~whether or not~~whether considered intervention-related (21 CFR 312.32 (a)).

8.3.2 DEFINITION OF SERIOUS ADVERSE EVENTS (SAE)

An adverse event (AE) or suspected adverse reaction is considered "serious" if, in the view of either the investigator or sponsor, it results in any of the following outcomes: death, a life-threatening adverse event, inpatient hospitalization or prolongation of existing hospitalization, a persistent or significant incapacity or substantial disruption of the ability to conduct normal life functions, or a congenital anomaly/birth defect. Important medical events that may not result in death, be life-threatening, or require hospitalization may be considered serious when, based upon appropriate medical judgment, they may jeopardize the participant and may require medical or surgical intervention to prevent one of the outcomes listed in this definition.

8.3.3 CLASSIFICATION OF AN ADVERSE EVENT

8.3.3.1 SEVERITY OF EVENT

- **Mild** – Events require minimal or no treatment and do not interfere with the participant's daily activities.
- **Moderate** – Events result in a low level of inconvenience or concern with the therapeutic measures. Moderate events may cause some interference with functioning.
- **Severe** – Events interrupt a participant's usual daily activity and may require systemic drug therapy or other treatment. Severe events are usually potentially life-threatening or incapacitating. Of note, the term "severe" does not necessarily equate to "serious".]

8.3.3.2 RELATIONSHIP TO STUDY INTERVENTION

All adverse events (AEs) must have their relationship to study intervention assessed by the clinician who examines and evaluates the participant based on temporal relationship and his/her clinical judgment. The degree of certainty about causality will be graded using the categories below. In a clinical trial, the study product must always be suspect.

- **Related** – The AE is known to occur with the study intervention, there is a reasonable possibility that the study intervention caused the AE, or there is a temporal relationship between the study intervention and event. Reasonable possibility means that there is evidence to suggest a causal relationship between the study intervention and the AE.

- **Not Related** – There is not a reasonable possibility that the administration of the study intervention caused the event, there is no temporal relationship between the study intervention and event onset, or an alternate etiology has been established.

OR

- **Definitely Related** – There is clear evidence to suggest a causal relationship, and other possible contributing factors can be ruled out. The clinical event, including an abnormal laboratory test result, occurs in a plausible time relationship to study intervention administration and cannot be explained by concurrent disease or other drugs or chemicals. The response to withdrawal of the study intervention (dechallenge) should be clinically plausible. The event must be pharmacologically or phenomenologically definitive, with use of a satisfactory rechallenge procedure if necessary.
- **Probably Related** – There is evidence to suggest a causal relationship, and the influence of other factors is unlikely. The clinical event, including an abnormal laboratory test result, occurs within a reasonable time after administration of the study intervention, is unlikely to be attributed to concurrent disease or other drugs or chemicals, and follows a clinically reasonable response on withdrawal (dechallenge). Rechallenge information is not required to fulfill this definition.
- **Potentially Related** – There is some evidence to suggest a causal relationship (e.g., the event occurred within a reasonable time after administration of the trial medication). However, other factors may have contributed to the event (e.g., the participant's clinical condition, other concomitant events). Although an AE may rate only as "possibly related" soon after discovery, it can be flagged as requiring more information and later be upgraded to "probably related" or "definitely related", as appropriate.
- **Unlikely to be related** – A clinical event, including an abnormal laboratory test result, whose temporal relationship to study intervention administration makes a causal relationship improbable (e.g., the event did not occur within a reasonable time after administration of the study intervention) and in which other drugs or chemicals or underlying disease provides plausible explanations (e.g., the participant's clinical condition, other concomitant treatments).
- **Not Related** – The AE is completely independent of study intervention administration, and/or evidence exists that the event is definitely related to another etiology. There must be an alternative, definitive etiology documented by the clinician.

8.3.3.3 EXPECTEDNESS

The PI and ISM will be responsible for determining whether an adverse event (AE) is expected or unexpected. An AE will be considered unexpected if the nature, severity, or frequency of the event is not consistent with the risk information previously described for the study intervention.

8.3.4 TIME PERIOD AND FREQUENCY FOR EVENT ASSESSMENT AND FOLLOW-UP

The occurrence of an adverse event (AE) or serious adverse event (SAE) may come to the attention of study personnel during study visits and interviews of a study participant presenting for medical care, or upon review by a study monitor.

All AEs including local and systemic reactions not meeting the criteria for SAEs will be captured on the appropriate case report form (CRF). Information to be collected includes event description, time of onset, clinician's assessment of severity, relationship to study product (assessed only by those with the training and authority to make a diagnosis), and time of resolution/stabilization of the event. All AEs occurring while on study must be documented appropriately regardless of relationship. All AEs will be followed to adequate resolution.

Any medical condition that is present at the time that the participant is screened will be considered as baseline and not reported as an AE. However, if the study participant's condition deteriorates at any time during the study, it will be recorded as an AE.

Changes in the severity of an AE will be documented to allow an assessment of the duration of the event at each level of severity to be performed. AEs characterized as intermittent require documentation of onset and duration of each episode.

The PI and ISM will record all reportable events with start dates occurring any time after informed consent is obtained until 7 (for non-serious AEs) or 30 days (for SAEs) after the last day of study participation. At each study visit, the investigator will inquire about the occurrence of AE/SAEs since the last visit. Events will be followed for outcome information until resolution or stabilization.

8.3.5 ADVERSE EVENT REPORTING

The PI (Susan Shore, PhD) will comply with the NIMH Reportable Events policy. The intervention will continue for each subject so long as adverse events do not occur for that individual. The ISM will monitor data and review safety data. If adverse events related to the study (definitely related, probably related, or potentially related) do occur, they will be reported to the IRB within 7 days and will be reported to the PI immediately. All other adverse events will be reported annually at the time of IRB renewal.

8.3.6 SERIOUS ADVERSE EVENT REPORTING

The study investigator shall complete an Unanticipated Adverse Device Effect Form and submit to the study sponsor and to the reviewing Institutional Review Board (IRB) as soon as possible, but in no event later than 10 working days after the investigator first learns of the effect. The study sponsor is responsible for conducting an evaluation of an unanticipated adverse device effect and shall report the results of such evaluation to the Food and Drug Administration (FDA)

and to all reviewing IRBs and participating investigators within 10 working days after the sponsor first receives notice of the effect. Thereafter, the sponsor shall submit such additional reports concerning the effect as FDA requests.

8.3.7 REPORTING EVENTS TO PARTICIPANTS

N/A

8.3.8 EVENTS OF SPECIAL INTEREST

N/A

8.3.9 REPORTING OF PREGNANCY

N/A

8.4 UNANTICIPATED PROBLEMS

8.4.1 DEFINITION OF UNANTICIPATED PROBLEMS (UP)

The Office for Human Research Protections (OHRP) considers unanticipated problems involving risks to participants or others to include, in general, any incident, experience, or outcome that meets **all** of the following criteria:

- Unexpected in terms of nature, severity, or frequency given (a) the research procedures that are described in the protocol-related documents, such as the Institutional Review Board (IRB)-approved research protocol and informed consent document; and (b) the characteristics of the participant population being studied;
- Related or possibly related to participation in the research ("possibly related" means there is a reasonable possibility that the incident, experience, or outcome may have been caused by the procedures involved in the research); and
- Suggests that the research places participants or others at a greater risk of harm (including physical, psychological, economic, or social harm) than was previously known or recognized.

This definition could include an unanticipated adverse device effect, any serious adverse effect on health or safety or any life-threatening problem or death caused by, or associated with, a device, if that effect, problem, or death was not previously identified in nature, severity, or degree of incidence in the investigational plan or application (including a supplementary plan or application), or any other unanticipated serious problem associated with a device that relates to the rights, safety, or welfare of subjects (21 CFR 812.3(s)).

8.4.2 UNANTICIPATED PROBLEM REPORTING

The investigator will report unanticipated problems (UPs) to the reviewing Institutional Review Board (IRB) and to the Data Coordinating Center (DCC)/lead principal investigator (PI). The UP report will include the following information:

- Protocol identifying information: protocol title and number, PI's name, and the IRB project number;
- A detailed description of the event, incident, experience, or outcome;
- An explanation of the basis for determining that the event, incident, experience, or outcome represents an UP;
- A description of any changes to the protocol or other corrective actions that have been taken or are proposed in response to the UP.

To satisfy the requirement for prompt reporting, UPs will be reported using the following timeline:

- UPs that are serious adverse events (SAEs) will be reported to the IRB and to the DCC/study sponsor within 10 days of the investigator becoming aware of the event.
- Any other UP will be reported to the IRB and to the DCC/study sponsor within 10 days of the investigator becoming aware of the problem.
- All UPs should be reported to appropriate institutional officials (as required by an institution's written reporting procedures), the supporting agency head (or designee), and the Office for Human Research Protections (OHRP) within 10 days of the IRB's receipt of the report of the problem from the investigator.

8.4.3 REPORTING UNANTICIPATED PROBLEMS TO PARTICIPANTS

N/A

9 STATISTICAL CONSIDERATIONS

9.1 STATISTICAL HYPOTHESES

The data will be collected throughout the course of the study by blinded test administrators. A General Linear/Logistic Mixed (GLLM) effects analytic model was developed in consultation with UM statistics department prior to our preliminary trial. GLLM is considered ideal when analyzing repeated measures as it treats time as a continuous variable and can account for missing data. This framework can readily adapt to subgroupings present in the data. GLLM analysis includes random and fixed effects. In this study, treatment order (active first vs sham first), treatment condition (active, active washout, sham, sham washout), baseline loudness, baseline TFI, and interaction of treatment and treatment order are treated as fixed while intercept is treated as a random effect. All metrics are computed relative to baseline for each subject.

MICHR has provided templates, tools, and general guidance in support of activities conducted by the study team to facilitate study and data ~~management, and~~management and will continue to do so. The University of Michigan's Department of Statistics Consulting for Statistics, Computing and Analytics Research (CSCAR) has provided individualized support and training to our team on management, collection, and analysis of data for phase 1 (which is identical to the second except in terms of subject numbers and treatment duration). Staff are available for continued support in the use of technical software, and advanced computing in research.

- Primary Efficacy Endpoint(s):

  1) Objective Tinnitus Loudness
  2) Tinnitus Functional Index
  3) Tinnitus Handicap Index

- Secondary Efficacy Endpoint(s):
  1) Tinnitus bandwidth/spectrum
  2) Minimum masking level
  3) Tinnitus Hearing Survey

9.2 SAMPLE SIZE DETERMINATION

To compute statistical power, we utilized a linear model of our prior trial data, where change from baseline in both TFI and loudness is a linear function of the time in study, with a constant term reflecting enrollment in study. Time in study was extended out to six weeks. Data sample independence was achieved by normalizing each subject by their own baseline. With these parameters, alpha = 0.05 and beta = 0.8, we simulated new data sets with samples ranging from 5 to 50. Model slopes were tested for significant differences using analysis of covariance. Each new model was simulated 50 times, and the total number of significant models counted. The first sample size where the computed power was greater than 0.8 was determined. 14 subjects are required per arm to achieve a power of 0.8 for the loudness test, and 17 subjects are required per arm for the TFI. Thus, this study design requires a total subject count of 34. We propose a minimum sample size of 50 to account for subject variability and compliance.

9.3 POPULATIONS FOR ANALYSES

All subject's data will be analyzed in one cohort. We will perform both per-protocol and intention-to-treat analyses. While intention-to-treat analysis is ideal, our protocol may require subjects to adhere to a strict schedule in order to fully realize the benefit of the treatment. Our pilot study had excellent compliance rates, but a longer and larger study provides more opportunity for non-compliant subjects. Should sensitivity analysis between the intention-to-treat and per-protocol analyses attain significance, differences between compliant and non-compliant subjects will be further explored in an effort more fully understand who best can benefit from the bimodal protocol. Missing data and losses to follow-up will be managed using

a last observation carried forward approach. Sensitivity analysis to outliers will also be performed. If results differ significantly between with and without outliers, outliers will be further investigated to determine what factors impact the likelihood of a subject responding or being resistant to the treatment protocol.

9.4 STATISTICAL ANALYSES

Data will be analyzed as means with standard deviations. All hypothesis-testing derived analyses will utilize an alpha of 0.05, beta of 0.8, and utilize two-tailed tests.

9.4.1 ANALYSIS OF THE PRIMARY EFFICACY ENDPOINT(S)

For all endpoints (Tinnitus loudness, TFI and THI), the following approach will be utilized. All measures are ordinal, repeated measures. Final analysis will occur after unblinding.

Tinnitus loudness is measured by having subjects match the loudness and likeness of their tinnitus to one of 11 sounds with different center frequencies. Absolute differences are averaged relative to baseline to determine loudness. TFI and THI are questionnaires to be completed at each weekly visit. Both are measured on an interval scale. See section 3.1 for more details

A General Linear/Logistic Mixed (GLLM) effects analytic model will be used. GLLM is considered ideal when analyzing repeated measures as it treats time as a continuous variable and can account for missing data. This framework can readily adapt to subgroupings present in the data. GLLM analysis includes random and fixed effects. In this study, treatment order (active first vs sham first), treatment condition (active, active washout, sham, sham washout), baseline loudness, baseline TFI, and interaction of treatment and treatment order are treated as fixed while intercept is treated as a random effect.

Adjustments for Type I error over the set of endpoints is unnecessary as the measures are not directly related.

9.4.2 ANALYSIS OF THE SECONDARY ENDPOINT(S)

The following analytical framework will be applied to 2ndary measures (see section 3.2 for more details). Analyses are not dependent on primary-measures. All measures are ordinal, repeated measures. Data will be analyzed using repeated-measures ANOVA, and shown as mean+/-SEM. Normality will be assessed using Kolmogorov-Smirnoff tests, and non-parametric tests used for non-normally distributed data. To assess how the secondary measures might co vary with the primary measures, we will use principal component analysis or linear fixed effects models.

Non-compliant and missing data points will be excluded from analysis.

9.4.3 SAFETY ANALYSES

All AEs will be classified recorded and reported as noted in the Sections above.

.

9.4.4 BASELINE DESCRIPTIVE STATISTICS

See statistical tests above. All analyzed metrics are computed for each subject's baseline.

9.4.5 PLANNED INTERIM ANALYSES

N/A

9.4.6 SUB-GROUP ANALYSES

N/A

9.4.7 TABULATION OF INDIVIDUAL PARTICIPANT DATA

State whether individual participant data will be listed by measure and time point.

Individual data will be listed by time point.

9.4.8 EXPLORATORY ANALYSES

To be determined.

10 SUPPORTING DOCUMENTATION AND OPERATIONAL CONSIDERATIONS

10.1 REGULATORY, ETHICAL, AND STUDY OVERSIGHT CONSIDERATIONS

10.1.1 INFORMED CONSENT PROCESS

10.1.1.1 CONSENT/ASSENT AND OTHER INFORMATIONAL DOCUMENTS PROVIDED TO PARTICIPANTS

Consent forms describing in detail the study intervention, study procedures, and risks are given to the participant and written documentation of informed consent is required prior to starting intervention/administering study intervention.

10.1.1.2 CONSENT PROCEDURES AND DOCUMENTATION

Informed consent is a process that is initiated prior to the individual's agreeing to participate in the study and continues throughout the individual's study participation. Consent forms will be Institutional Review Board (IRB)-approved and the participant will be asked to read and review

the document. The investigator will explain the research study to the participant and answer any questions that may arise. A verbal explanation will be provided in terms suited to the participant's comprehension of the purposes, procedures, and potential risks of the study and of their rights as research participants. Participants will have the opportunity to carefully review the written consent form and ask questions prior to signing. The participants should have the opportunity to discuss the study with their family or surrogates or think about it prior to agreeing to participate. The participant will sign the informed consent document prior to any procedures being done specifically for the study. Participants must be informed that participation is voluntary and that they may withdraw from the study at any time, without prejudice. A copy of the informed consent document will be given to the participants for their records. The informed consent process will be conducted and documented in the source document (including the date), and the form signed, before the participant undergoes any study-specific procedures. The rights and welfare of the participants will be protected by emphasizing to them that the quality of their medical care will not be adversely affected if they decline to participate in this study.

10.1.2 STUDY DISCONTINUATION AND CLOSURE

This study may be temporarily suspended or prematurely terminated if there is sufficient reasonable cause. Written notification, documenting the reason for study suspension or termination, will be provided by the suspending or terminating party to investigator, and funding agency. If the study is prematurely terminated or suspended, the Principal Investigator (PI) will promptly inform study participants, the Institutional Review Board (IRB), and sponsor and will provide the reason(s) for the termination or suspension. Study participants will be contacted, as applicable, and be informed of changes to study visit schedule.

Circumstances that may warrant termination or suspension include, but are not limited to:

- Determination of unexpected, significant, or unacceptable risk to participants
- Demonstration of efficacy that would warrant stopping
- Insufficient compliance to protocol requirements
- Data that are not sufficiently complete and/or evaluable
- Determination that the primary endpoint has been met
- Determination of futility

Study may resume once concerns about safety, protocol compliance, and data quality are addressed, and satisfy the sponsor, IRB and/or Food and Drug Administration (FDA).

10.1.3 CONFIDENTIALITY AND PRIVACY

Participant confidentiality and privacy is strictly held in trust by the participating investigators, their staff, and the sponsor(s) and their interventions. This confidentiality is extended to cover testing of biological samples and genetic tests in addition to the clinical information relating to participants. Therefore, the study protocol, documentation, data, and all other information

generated will be held in strict confidence. No information concerning the study or the data will be released to any unauthorized third party without prior written approval of the sponsor.

All research activities will be conducted in as private a setting as possible.

The ISM other authorized representatives of the sponsor, representatives of the Institutional Review Board (IRB), regulatory agencies or pharmaceutical company supplying study product may inspect all documents and records required to be maintained by the investigator, including but not limited to, medical records (office, clinic, or hospital) and pharmacy records for the participants in this study. The clinical study site will permit access to such records.

The study participant's contact information will be securely stored at each clinical site for internal use during the study. At the end of the study, all records will continue to be kept in a secure location for as long a period as dictated by the reviewing IRB, Institutional policies, or sponsor requirements.

Study participant research data, which is for purposes of statistical analysis and scientific reporting, will be transmitted to and stored with MICHR's REDCap Service (https://www.michr.umich.edu/redcap-access). This will not include the participant's contact or identifying information. Rather, individual participants and their research data will be identified by a unique study identification number. The study data entry and study management systems used by clinical sites and by MICHR's REDCap Service research staff will be secured and password protected. At the end of the study, all study databases will be de-identified and archived at the NIMH database.

10.1.4 FUTURE USE OF STORED SPECIMENS AND DATA

Data collected for this study will be analyzed and stored with MICHR's REDCap Service. After the study is completed, the de-identified, archived data will be transmitted to and stored at the NIMH data repository for use by other researchers including those outside of the study. Permission to transmit data to the NIMH data repository will be included in the informed consent.

10.1.5 KEY ROLES AND STUDY GOVERNANCE

Provide the name and contact information of the Principal Investigator and the Independent Safety Monitor.

**Principal Investigator**	**Independent Safety Monitor**
Susan Shore, PhD	*MICHR*
University of Michigan	*University of Michigan*
1150 W. Medical Center Drive, Ann Arbor MI 48109	*1600 Huron Parkway Bldg 400 Ann Arbor, MI 48105*

734-647-2116	*734-998-7474*
sushore@umich.edu	*UM-MICHR@umich.edu*

10.1.6 SAFETY OVERSIGHT

Safety oversight will be under the direction of the PI and the ISM.
The ISM is independent from the study conduct and free of conflict of interest, or measures should be in place to minimize perceived conflict of interest. The ISM and PI will meet at least semiannually to assess safety and efficacy data on each arm of the study. The ISM will operate under the rules of an approved charter that will be written and reviewed at the organizational meeting of the ISM. At this time, each data element that the ISM needs to assess will be clearly defined. The ISM will provide its input to NIMH.

10.1.7 CLINICAL MONITORING

N/A

10.1.8 QUALITY ASSURANCE AND QUALITY CONTROL

N/A

10.1.9 DATA HANDLING AND RECORD KEEPING

10.1.9.1 DATA COLLECTION AND MANAGEMENT RESPONSIBILITIES

Data collection is the responsibility of the clinical trial staff at the site under the supervision of the site investigator. The investigator is responsible for ensuring the accuracy, completeness, legibility, and timeliness of the data reported.

All source documents should be completed in a neat, legible manner to ensure accurate interpretation of data. All data will be captured by custom developed software from the Shore Lab, a 21 CFR Par 11-compliant system, and automatically saved on the previously identified encrypted servers. Data include audiograms, TFI, THI, use/compliance monitoring, etc. All data will be centrally collected, stored on an ongoing basis.

Hardcopies of the study visit worksheets will be provided for use as source document worksheets for recording data for each participant enrolled in the study. Data recorded in the electronic case report form (eCRF) derived from source documents should be consistent with the data recorded on the source documents. Source code, documentation, user manuals and monitoring analyses will be made available on completion of system. Data collected by devices will be encrypted locally on each device, and accessible only to study investigators with appropriate passwords.

Clinical data (including adverse events (AEs), concomitant medications, and expected adverse reactions data) and clinical laboratory data will be entered into MICHR REDCap, a 21 CFR Part 11-compliant data capture system provided by the MICHR REDCap Service. The data system includes password protection and internal quality checks, such as automatic range checks, to identify data that appear inconsistent, incomplete, or inaccurate. Clinical data will be entered directly from the source documents.

All data will be captured by custom developed software and automatically saved on the previously identified encrypted servers. Data include audiograms, TFI, THI, use/compliance monitoring, etc. All data will be centrally collected, stored on an ongoing basis.

Source code, documentation, user manuals and monitoring analyses will be made available on completion.

Data collected by devices will be encrypted locally on each device, and accessible only to study investigators with appropriate passwords.

10.1.9.2 STUDY RECORDS RETENTION

All records and information collected as part of participation in this study will be kept for 7 years from the date the study is completed.

10.1.10 PROTOCOL DEVIATIONS

The investigator is responsible for complying with the protocol and all appropriate regulations and guidelines governing global clinical research. Additionally, he/she is responsible for ensuring that all participating staff members are adequately trained and competent to perform his/her assigned tasks.

Modifications to the study protocol will not be implemented by either the sponsor or the investigator without agreement by both parties. However, the investigator may implement a deviation form, or a change of, the protocol to eliminate an immediate hazard(s) to the trial subjects without prior IRB/sponsor approval/favorable opinion. As soon as possible, the implemented deviation or change, the reasons for it and if appropriate the proposed protocol amendment should be submitted to the IRB/sponsor.

Deviations from the protocol must be recorded and reported (per local IRBMED guidelines) by the investigator. The circumstances, action taken and impact of major deviations on the trial must be communicated by the Principal Investigator to the designated ISM.

10.1.11 PUBLICATION AND DATA SHARING POLICY

This study will be conducted in accordance with the following publication and data sharing policies and regulations:

National Institutes of Health (NIH) Public Access Policy, which ensures that the public has access to the published results of NIH funded research. It requires scientists to submit final peer-reviewed journal manuscripts that arise from NIH funds to the digital archive PubMed Central upon acceptance for publication.

This study will comply with the NIH Data Sharing Policy and Policy on the Dissemination of NIH-Funded Clinical Trial Information and the Clinical Trials Registration and Results Information Submission rule. As such, this trial will be registered at ClinicalTrials.gov, and results information from this trial will be submitted to ClinicalTrials.gov. In addition, every attempt will be made to publish results in peer-reviewed journals. In addition, in accordance with the funding source, data will be shared on the NIMH Data Archive.

10.1.12 CONFLICT OF INTEREST POLICY

The independence of this study from any actual or perceived influence, such as by the pharmaceutical industry, is critical. Therefore, any actual conflict of interest of persons who have a role in the design, conduct, analysis, publication, or any aspect of this trial will be disclosed and managed. Furthermore, persons who have a perceived conflict of interest will be required to have such conflicts managed in a way that is appropriate to their participation in the design and conduct of this trial. The study leadership in conjunction with the <specify NIH Institute or Center (IC)> has established policies and procedures for all study group members to disclose all conflicts of interest and will establish a mechanism for the management of all reported dualities of interest.

Susan Shore and David Martel are inventors on U.S. patent no. 3A9242067 “Personalized auditory-somatosensory stimulation to treat tinnitus.” We have disclosed this conflict to the UMMS Conflict of Interest Board (MEDCOI) and will seek their review of decisions pertaining to the actual or perceived conflicts.

10.2 ADDITIONAL CONSIDERATIONS

N/A

10.3 ABBREVIATIONS

AE	Adverse Event
ANOVA	Analysis of Variance
ANSI	American National Standards Institute
CN	Cochlear Nucleus
CONSORT	Consolidated Standards of Reporting Trials
CPU	Central Processing Unit
CRF	Case Report Form
CSCAR	Consulting for Statistics, Computing and Analytics Research
DCC	Data Coordinating Center
DCN	Dorsal Cochlear Nucleus
DSMB	Data Safety Monitoring Board
eCRF	Electronic Case Report Forms
FDA	Food and Drug Administration
GLMM	Generalized Linear Mixed Modeling
IHS	Intelligent Hearing Systems
IRB	Institutional Review Board
ISM	Independent Safety Monitor
ISO	International Organization for Standardization
MICHR	Michigan Institute for Clinical & Health Research
MML	Minimum Masking Level
NIH	National Institutes of Health
NIMH	NIH National Institute of Mental Health
OSHA	Occupational Safety and Health Administration
PI	Principal Investigator
SAE	Serious Adverse Event
SOA	Schedule of Activities
STDP	Spike Timing Dependent Plasticity
StDP	Stimulus Timing Dependent Plasticity
TFI	Tinnitus Functional Index
THI	Tinnitus Hearing Survey
THS	Tinnitus Hearing Survey
UP	Unanticipated Problem

10.4 PROTOCOL AMENDMENT HISTORY

The table below is intended to capture changes of IRB-approved versions of the protocol, including a description of the change and rationale. A Summary of Changes table for the current amendment is located in the Protocol Title Page.

Version	Date	Description of Change	Brief Rationale
1.2	11/6/2018	Amending inclusion criteria to include a subject "must have no greater than a mild hearing loss 125-6000 Hz".	This is so we are able to have similar hearing profiles from Stage 1 of the clinical trial.
1.3	12/3/2018	Remove TinnTester from the screening appointment.	The TinnTester is not needed to determine eligibility.
1.3	12/3/2018	Complete the tinnitus modulation checklist throughout the study, specifically on Day 42, 91, 133, and 175.	To evaluate the effect of the device on the somatosensory system.
1.3	12/3/2018	Remove Auditory Brainstem Response (ABR) from study.	Not completing this measure because it is not a primary objective for study aim.
1.3	12/3/2018	Remove Residual Inhibition (RI) from study.	Not completing this measure because it is not a primary objective for study aim.
1.4	1/29/2019	On page 40, deleted "Medical" and inserted "Independent Study".	The monitor is not a medical monitor.
1.5	3/29/2019	On page 4, added "central nervous system stimulants" and "benzodiazepines" to the medication exclusion criteria.	These drugs may be causing, contributing to, and influencing one's tinnitus.
1.5	3/29/2019	On page 2, 7, and 18, changed "100" people consented to "200" people consented.	We need to expand this number in order to reach 50 people enrolled in the study.
1.6	1/27/2020	On page 10, added sentence stating that though unlikely, it is possible that tinnitus may become louder or more noticeably during the trial.	This was added to reflect occurrences in the trial and changes on the consent form.
1.6	1/27/2020	On pages 2, 7, and 18, updated the total number of participants from N=200 to N=300	To allow more screenings in order to reach goal of 50 subjects actively enrolled.
1.11	4/14/21	On pages 2, 7, and 18, updated the total number of participants from N=300 to N=400	To allow more screenings in order to reach goal of 50 subjects with complete data

1.11	4/14/21	On pages 2, 18, changed 50 randomized subjects to up to 100 randomized subjects	To allow more subjects to reach goal of 50 subjects with complete data
7.1, 8.2	6/22/2021	Pg 24, 29, Clarifying exclusion criteria	Clarifying exclusion criteria with TFI and TT
8.2	6/22/2021	Pg 29, added online location for consenting and medical screenings, added 2nd floor of Med Sci I for medical screenings, added Dr. Stucken for medical referrals as needed	To prevent excessive in person appointments and allow Dr. Stucken to refer as needed
8.2	2/16/2022	Pg 29, changed adverse event reporting time from 7 days to 14 days to match IRBMED	Changing adverse event reporting time to match IRBMED

11 REFERENCES

Dobie, R. A. (1999). "A review of randomized clinical trials in tinnitus." Laryngoscope **109**(8): 1202-1211.

OBJECTIVES: Review reports of randomized clinical trials (RCTs) in tinnitus to identify well-established treatments, promising developments, and opportunities for improvement in this area of clinical research. STUDY DESIGN: Literature review of RCTs (1964-1998) identified by MEDLINE and OLD MEDLINE searches and personal files. METHODS: Studies were compared with the RCT criteria of Guyatt et al. for quality of design, performance, and analysis; "positive" results were critically examined for potential clinical relevance. RESULTS: Sixty-nine RCTs evaluated tocainide and related drugs, carbamazepine, benzodiazepines, tricyclic antidepressants, 16 miscellaneous drugs, psychotherapy, electrical/magnetic stimulation, acupuncture, masking, biofeedback, hypnosis, and miscellaneous other nondrug treatments. No treatment can yet be considered well established in terms of providing replicable long-term reduction of tinnitus impact, in excess of placebo effects. CONCLUSIONS: Nonspecific support and counseling are probably helpful, as are tricyclic antidepressants in severe cases. Benzodiazepines, newer antidepressants, and electrical stimulation deserve further study. Future tinnitus therapeutic research should emphasize adequate sample size, open trials before RCTs, careful choice of outcome measures, and long-term follow-up.

Marks, K. L., et al. (2018). "Auditory-somatosensory bimodal stimulation desynchronizes brain circuitry to reduce tinnitus in guinea pigs and humans." Sci Transl Med **10**(422).

The dorsal cochlear nucleus is the first site of multisensory convergence in mammalian auditory pathways. Principal output neurons, the fusiform cells, integrate auditory nerve inputs from the cochlea with somatosensory inputs from the head and neck. In previous work, we developed a guinea pig model of tinnitus induced by noise exposure and showed that the fusiform cells in these animals exhibited increased spontaneous activity and cross-unit synchrony, which are physiological correlates of tinnitus. We delivered repeated bimodal auditory-somatosensory stimulation to the dorsal cochlear nucleus of guinea pigs with tinnitus, choosing a stimulus interval known to induce long-term depression (LTD). Twenty minutes per day of LTD-inducing bimodal (but not unimodal) stimulation reduced physiological and behavioral evidence of tinnitus in the guinea pigs after 25 days. Next, we applied the same bimodal treatment to 20 human subjects with tinnitus using a double-blinded, sham-controlled, crossover study. Twenty-eight days of LTD-inducing bimodal stimulation reduced tinnitus loudness and intrusiveness. Unimodal auditory stimulation did not deliver either benefit. Bimodal auditory-somatosensory stimulation that induces LTD in the dorsal cochlear nucleus may hold promise for suppressing chronic tinnitus, which reduces quality of life for millions of tinnitus sufferers worldwide.

TRIAL FULL TITLE	Reversing Synchronized Brain Circuits with Targeted Auditory-Somatosensory Stimulation to Treat Phantom percepts – stage 2
SAP VERSION	1.0
SAP VERSION DATE	January 20, 2023
TRIAL STATISTICIAN	Joshua Errickson, PhD
Protocol Version (SAP associated with)	1.12
TRIAL PRINCIPAL INVESTIGATOR	Susan Shore, PhD
SAP AUTHOR(s)	Josh Errickson, PhD; Susan E. Shore, PhD<br>David Martel, PhD

1 SAP Signatures

I give my approval for the attached SAP entitled *Reversing Synchronized Brain Circuits with Targeted Auditory-Somatosensory Stimulation to Treat Phantom percepts – stage 2,* dated January 26, 2023

Statistician (Author)
Name: Josh Errickson, PhD

Signature: Josh Errickson

Date: January 27, 2023

Principal Investigator
Name: Susan E. Shore, PhD

Signature: Susan E. Shore

Date: January 27, 2023

2 Table of Contents

1 SAP Signatures 1

2 Table of Contents 2

3 Abbreviations and Definitions 4

4 Introduction 5

4.1 Preface 5

4.2 Scope of the analyses 5

5 Study Objectives and Endpoints 5

5.1 Study Objectives 5

5.1.1 Primary Objective: 5

5.1.2 Secondary Objectives: 5

5.2 Study Endpoints 6

5.2.1 Primary Endpoints: 6

6 Study Methods 6

6.1 General Study Design and Plan 6

6.2 Inclusion-Exclusion Criteria and General Study Population 6

7.2 Randomization and Blinding 8

7.3 Study Assessments 8

8 Sample Size 12

9 General Analysis Considerations 12

9.2 Timing of Analyses 12

9.3 Analysis Populations 12

9.3.3 Safety/Intent-to-treat (ITT) Cohort 12

9.3.4 Modified Intent-to-treat Cohort (mITT) 12

9.3.5 Per-Protocol Cohort (PP) 13

9.3.6 Cohort for Primary and Secondary Endpoints 13

9.4 Covariates and Subgroups 13

9.5 Missing Data 13

9.6 Multiple Testing 14

10 Summary of Study Data 14

10.1 Subject Disposition 14

10.2 Derived variables 14

10.3 Protocol Deviations 14

10.4 Demographic and Baseline Variables 15

10.5 Concurrent Illnesses and Medical Conditions 15

10.6 Treatment Compliance 15

11 Efficacy Analyses 15

11.1 Primary Efficacy Analysis 15
11.2 Secondary Efficacy Analyses 15
11.2.1 Secondary Analyses of Primary Efficacy Endpoint 15
11.2.2 Analyses of Secondary Endpoints 15
11.3 Exploratory Efficacy Analyses 16
12 Safety Analyses 16
12.1 Extent of Exposure 16
12.2 Adverse Events 16
12.3 Deaths, Serious Adverse Events, and other Significant Adverse Events 16
12.4 Pregnancies (As applicable) 16
12.5 Prior and Concurrent Medications 16
12.6 Other Safety Measures 17
13 Reporting Conventions 17
14 Quality Assurance of Statistical Programming 17
15 Summary of Changes to the Protocol and/or SAP 17
16 References 19

3 Abbreviations and Definitions

AE	Adverse Event
ANOVA	Analysis of Variance
ANSI	American National Standards Institute
CN	Cochlear Nucleus
CONSORT	Consolidated Standards of Reporting Trials
CPU	Central Processing Unit
CRF	Case Report Form
CSCAR	Consulting for Statistics, Computing and Analytics Research
DCC	Data Coordinating Center
DCN	Dorsal Cochlear Nucleus
DSMB	Data Safety Monitoring Board
eCRF	Electronic Case Report Forms
FDA	Food and Drug Administration
GLMM	Generalized Linear Mixed Modeling
IHS	Intelligent Hearing Systems
IMP	Investigational Medical Product
IRB	Institutional Review Board
ISM	Independent Safety Monitor
ISO	International Organization for Standardization
MICHR	Michigan Institute for Clinical & Health Research
MML	Minimum Masking Level
NIH	National Institutes of Health
NIMH	NIH National Institute of Mental Health
OSHA	Occupational Safety and Health Administration
PI	Principal Investigator
SAE	Serious Adverse Event
SAP	Statistical Analysis Plan
SOA	Schedule of Activities
STDP	Spike Timing Dependent Plasticity
StDP	Stimulus Timing Dependent Plasticity
TFI	Tinnitus Functional Index
THI	Tinnitus Handicap Inventory
THS	Tinnitus Hearing Survey
UP	Unanticipated Problem

4 Introduction

4.1 Preface

Preclinical work in our lab has shown that tinnitus, the phantom perception of sound, is generated by neurons in a brainstem station, the dorsal cochlear nucleus. Here, primary neurons become hyperactive after tinnitus induction in an animal model. Interestingly, the degree of hyperactivity, and thus tinnitus, can be modulated though auditory-somatosensory (bimodal) stimulation. We've shown that specially-timed intervals can suppress the tinnitus-initiating neurons, and thereby reduce tinnitus in the animal model. Remarkably, the same stimulus protocol can reduce tinnitus loudness and life impact in human adults, as demonstrated in our previous stage 1 clinical trial (1).

The present stage 2 trial is a prospective, single-center, randomized, double-blind, sham-controlled, two-period crossover study to evaluate the safety and effectiveness of a bimodal auditory-somatosensory stimulation protocol in reducing the tinnitus loudness and life-impact in adults with tinnitus.

4.2 Scope of the analyses

These analyses will be used to assess the efficacy and safety of bi-sensory auditory-somatosensory stimulation compared to auditory alone stimulation and will be included in the clinical study report.

Test parameters/dosages for modulation of multisensory plasticity in human subjects with changes in tinnitus severity as the outcome measure.

Hypothesis: Stimuli identified in pre-clinical studies, with optimal dosage/parameters, will effectively modulate DCN circuitry to reduce tinnitus in humans.

Experiment 1a: Using a double-blind cross-over model, we will use a bi-sensory auditory-somatosensory paradigm with optimal stimulus parameters based on pre-clinical studies to assess changes in subjects' tinnitus perception. Modulations in tinnitus perception will be assessed using objective loudness matching and subjective measures (standard questionnaires) in response to active and sham bimodal stimuli. Neural circuitry changes will be assessed by examining changes in the severity of subjects' tinnitus. It is anticipated that chosen bimodal intervals and durations of treatment (dose) will reduce the abnormal percepts.

Experiment 1b: Subjects will be monitored during a washout period for a return to baseline to determine durability of effect. To assess dose-response of the bimodal modulation, the length of bimodal stimulation treatment will be varied and effects and duration of effect on tinnitus will be assessed as above.

5 Study Objectives and Endpoints

5.1 Study Objectives

5.1.1 Primary Objective:

To reduce tinnitus loudness and life impact.

5.1.2 Secondary Objectives:

To alter the tinnitus spectrum and minimum masking level.

5.2 Study Endpoints

5.2.1 Primary Endpoints:

1. Change in Tinnitus Loudness Spectrum (TinnTester Interactive Software)
2. Tinnitus Functional Index (TFI) questionnaire
3. Tinnitus Handicap Inventory (THI)

5.2.2 Secondary Endpoints:

1. Tinnitus bandwidth (TinnTester Interactive Software)
2. Tinnitus Hearing Survey (THS)
3. Minimum Masking Levels (MML)

6 Study Methods

6.1 General Study Design and Plan

This is a prospective, single-center, randomized, double-blind, sham-controlled, two-period crossover study to evaluate the safety and effectiveness of a bimodal auditory-somatosensory stimulation protocol in reducing the tinnitus loudness and life-impact in adults with tinnitus. The analysis seeks to evaluate the superiority of bimodal stimulation (Active treatment) compared to unimodal auditory stimulation (Control treatment).

Participants are assigned to Sequence Group 1 or 2 (Sequence Group 1 = Active treatment first then Control treatment; Sequence Group 2 = Control treatment first then Active treatment) using a balanced (1 Active First:1 Control First) randomization. Subjects are randomized prior to the first treatment, but after screening. Each treatment period includes one 6-week period of treatment followed by a 6-week washout period. Subjects are followed for a final 12-weeks follow-up after they complete their washout of the second treatment period.

Safety is evaluated by analysis of reported adverse events. The primary effectiveness endpoint is a subject's mean tinnitus loudness spectrum (from TinnTester) after 6-weeks of treatment with 6 weeks of washout, followed by an additional 6 weeks of treatment (t=18 weeks), compared between the Active Treatment Period and the Control Treatment Period. Additional measures of effectiveness include the Tinnitus Functional Index (TFI), the Tinnitus Handicap Inventory (THI), MML and various other Patient Reported Outcomes and audiometric parameters.

Subject compliance is enforced by and logged through the treatment device. Logs are recorded by study investigators at every in-person visit.

6.2 Inclusion-Exclusion Criteria and General Study Population

6.2.1 Study Population

400 male or female, adult (18 years of age and older) subjects with somatic tinnitus will be consented with a goal of including up to 100 subjects in the study. 50 will receive the Active treatment first (Sequence Group 1) while the other will receive the Control treatment first (Sequence Group 2).

6.2.2 Inclusion criteria:

1. Must be 18 years of age or older.
2. A score of >17 points on the Tinnitus Functional Index, as measured at the screening visit.
3. Must report constant, subjective, preferably unilateral tinnitus without any active external or middle ear pathology.
4. No greater than a moderate hearing loss at the tinnitus frequencies (≤55 dB HL) and no greater than a moderate hearing loss (≤50 dB HL) from 125 – 6000 Hz.
5. Must be able to modulate their tinnitus with a somatic maneuver.
6. Preferably onset of tinnitus less than one year ago but present for at least 6 months. Tinnitus should be bothersome.
7. Absence of retrocochlear pathology or VIIIth nerve lesion
8. No participation in a tinnitus treatment regimen within the past six months or participation in the UM stage 1 clinical trial.
9. Resides within 100 miles of the study site.

6.2.3 Exclusion criteria:

1. Anyone not meeting the inclusion criteria, and in addition:
2. Diagnosis of Meniere's disease
3. Diagnosis of Semicircular Canal Dehiscence
4. Unilateral or bilateral cochlear implant recipients
5. Diagnosis of acoustic neuroma
6. Reports their tinnitus is pulsating.
7. Evidence of retrocochlear disease
8. Patients with any indwelling electronic stimulation devices
9. Current or previous use of any acoustic hearing aid or over the counter personal sound amplification product (PSAP) in the last 6 months
10. Current or former use of any of the following medications within the past 6 months: high dose aspirin (>325mg/day), high dose NSAIDs (ibuprofen, Motrin, Celebrex) (>800mg/day), narcotics (any opioids), lithium, clonazepam, oxazepam, cholinergic medications (anti-dementia medications), anti-depressant medications (serotonin specific reuptake inhibitors; SSRIs, tri-cyclic anti-depressants; TCAs); anti-seizure/convulsant medications (Depakote), anti-psychotic medications (Haldol, Seroquel), Ototoxic medications, other chemotherapy agents, benzodiazepines, and central nervous system stimulants.
11. Current diagnoses of any of the following: obsessive compulsive disorder (OCD), schizophrenia, bipolar disorder, extreme generalized anxiety disorder, drug/alcohol dependence.
12. Pregnant or nursing.

6.2.4 Initial Assessment:

Participant's eligibility will be assessed at an initial examination conducted by an otologist and audiologist consisting of otoscopy, audiometry, tinnitus modulation checklist, case history questionnaire, and the Tinnitus Functional Index (TFI) questionnaire.

6.2.5 Lifestyle considerations:

During this study, participants are asked to:
1. Refrain from participation in any other tinnitus treatment
2. Report any changes in health status.
3. Report any changes in prescribed or over-the-counter medications.

7.2 Randomization and Blinding

Subjects are randomly assigned to a Sequence Group using the Mathworks MATLAB *randsample* command with a predetermined, fixed, secret seed value. *Randsample* generates random numbers using the Mersenne twister algorithm. We will enroll subjects in a 1:1 allocation ratio to create a balanced study design. Integer multiples of the first 50 assignments were utilized for subject enrollments >= 51. To ensure blinding, group assignment is generated *in silico* and assignment programmed into each device, without the intervention of a study member. This parameter is not made accessible to study members without password authorization. Devices are encrypted to prevent study team members or subjects from accessing device data in an unauthorized manner.

As the randomization list is generated algorithmically, the code can be reproduced with knowledge of the secret seed value. Unblinding may occur at the end of the study or in the case of an SAE related to the study (worsening of tinnitus, worsening of hearing threshold). Study investigators that do not have contact with subjects may unblind. Intentional and unintentional unblinding must be reported to the principal investigator.

Imperfect blinding could arise from the presence of the electrical stimulation present during active treatment. To avoid unblinding, subjects will be told that they may or may not feel electrical stimulation during both phases of the study. The previous study suggested that subjects tend to acclimate to the presence or lack of electrical stimulation during a day's treatment. Subjects will be discouraged from divulging whether they can feel the electrical stimulus.

No investigators are allowed to remain unblinded for the duration of the study. All analyses that could require unblinding (SAEs, worsening of a subject's tinnitus/hearing) will be identified by software and manual investigation, with the investigators notified by automated communication methods.

7.3 Study Assessments

1. Tinnitus loudness spectrum: The TinnTester software package (McMaster University, supplied by Dr. Larry Roberts) is an interactive computer program for assessing multiple psychophysical measures of tinnitus (2). This software package has been used extensively by the tinnitus research community and is published and validated by repeated evaluations (2-4). The TinnTester sounds will be presented by calibrated circumaural headphones in a soundproof booth. TinnTester limits sound output pressure levels to safe levels (<= 95 dB SPL for all frequencies). To assess loudness, participants adjust the level of each of 11 narrow band sounds differing in center frequency to match the level of their tinnitus. This gives a measure of loudness in sound pressure level (SPL) for each participant (*loudness matching method*). Data are recorded automatically by the TinnTester software package.
2. Tinnitus Functional Index (TFI): The 25-item version of the TFI determines severity of tinnitus on a 100-point numeric scale (scores above 17 indicate that tinnitus is bothersome) based on the participant's responses to 25 questions regarding the

impact tinnitus has on their emotional, social, and mental well-being. A thirteen-point reduction in score is considered clinically significant, indicating a noticeable improvement in tinnitus. This version has recently been validated for the test-retest reliability and internal consistency in New Zealand and the UK.

3. Tinnitus Handicap Inventory (THI): This is a 25-item questionnaire which assesses subjective impacts of tinnitus related to quality of life on a 100-point numeric scale. Scores 17 points or higher are considered “bothersome tinnitus.” This measure has been included as other studies of tinnitus utilize it.
4. Tinnitus bandwidth: At the onset, the TinnTester software asks participants to indicate which of three sounds best resembles their tinnitus; 5 kHz pure tone (“tonal” tinnitus), a narrow band noise centered at 5 kHz (±5% of CF, BPN5 masker, “ringing” tinnitus), or a wider band of noise centered at 5 kHz (±15% of CF, BPN15 masker, “hissing” tinnitus). We will relate treatment outcomes to tinnitus bandwidth by these measurements. We will also be able to determine whether the treatment alters bandwidth.
5. Tinnitus Likeness spectrum: After the tinnitus loudness spectrum measure in TinnTester, the participants then rate each of the same previously-tested 11 sounds for the similarity of its frequency to the frequency of their tinnitus (0 to 100 VAS scale). Sound levels are set at equal loudness levels. This gives the tinnitus frequency spectrum for each participant (*likeness matching method*).
6. Minimum Masking Level (MML): The TinnTester software asks participants adjust the level of a 2 kHz highpass noise sound until they can no longer perceive their tinnitus and is reported in dB SPL. MML may prove sensitive to the bimodal treatment procedure. MML should be reduced if tinnitus assessed by loudness matching is also reduced. However, even when tinnitus loudness is reduced, there are participants for whom tinnitus cannot be masked. The MML is being included as it has been used in several other studies of tinnitus.
7. Tinnitus Hearing Survey: A brief nine question survey which determines self-perceived handicap due to tinnitus, hearing loss, and hyperacusis. This questionnaire differs from the TFI and THI in that it qualifies any perceived handicap due to hearing loss and/or sound sensitivity (hyperacusis).

Assessments time points are defined at every seven days post-first-visit (Baseline; day 0). Data will be assigned to a week if it is captured +/- 3 days relative to a time point.

Table 1. Trial Timetable

ENDPOINTS	***TIMEPOINTS***	***JUSTIFICATION***
Tinnitus Functional Index	*1) Baseline*<br>*2) All study follow-up visits*	*To determine how use of System affects the tinnitus and quality of life after the active and sham treatment periods of the study.*
Tinnitus Handicap Inventory	*1) Baseline*<br>*2) All study follow-up visits prior to extended washout follow-up phase*	*To determine how use of System affects the tinnitus and quality of life before and after the active and sham treatment periods of the study*

ENDPOINTS	***TIMEPOINTS***	***JUSTIFICATION***
Tinnitus loudness spectrum (TinnTester)	*1) Baseline<br>2) All study follow-up visits prior to extended washout follow-up phase*	*To determine how use of System affects tinnitus loudness after the active and sham treatment periods of the study.*
Tinnitus bandwidth (TinnTester)	*1) Baseline<br>2) All study follow-up visits prior to extended washout follow-up phase*	*To determine how use of the system may or may not change the perceived frequency of tinnitus following the active or sham treatment periods*
Tinnitus Likeness spectrum (TinnTester)	*1) Baseline<br>2) All study follow-up visits prior to extended washout follow-up phase*	*To determine how use of the system may or may not change the perceived spectra of tinnitus following the active or control treatment periods*
Minimum Masking Level (MML)	*1) Baseline<br>2) End of 1st treatment period<br>3) End of first washout<br>4) End of 2nd treatment period<br>5) End of second washout*	*To determine if the MML for a 5kHz tone is reduced by the active treatment.*
Tinnitus Hearing Survey	*1) Baseline<br>2) End of 1st treatment period<br>3) End of first washout<br>4) End of 2nd treatment period<br>5) End of second washout*	*To determine if the active treatment has an effect or interaction with perceived handicap due to hearing loss and sound level tolerance*

Figure 1. Trial Schema

**Period**		**PERIOD 1 (6 WEEKS)**			**WASHOUT 1 (6W)**		**PERIOD 2 (6 WEEKS)**		**WASHOUT 2 (6W)**		**EXT W/O FOLLOW-UP**
**Visit**	Screen	Baseline	End of each week	End of W6	End of W7-11	End of W12	End of each week	End of W18	End of W19-23	End of W24	End of W28, 32, 36
**Day**		0	7, 14, 21, 28, 35	42	49-84	91	98, 105, 112, 119, 126	133	140-168	175	252
**Range**		-	+/- 3D	+/- 3D	+/- 3D	+/- 3D	+/- 3D	+/- 3D	+/- 3D	+/- 3D	+/- 3D
Informed consent	✓										
Medical history	✓										
Tinnitus maneuver	✓										
Audiogram	✓	✓		✓		✓		✓		✓	
TFI	✓	✓	✓	✓	✓	✓	✓	✓	✓	✓	✓
THI		✓	✓	✓	✓	✓	✓	✓	✓	✓	
Tinnitus loudness, spectrum		✓	✓	✓	✓	✓	✓	✓	✓	✓	
THS		✓		✓		✓		✓		✓	
MML		✓		✓		✓		✓		✓	
Adverse events			✓	✓	✓	✓	✓	✓	✓	✓	✓

8 Sample Size

We utilized a linear model of our prior trial data, where change from baseline in both TFI and loudness is a linear function of the time in study, with a constant term reflecting enrollment in study. Time in study was extended out to six weeks. Data sample independence was achieved by normalizing each subject by their own baseline. With these parameters, alpha = 0.05 and beta = 0.8, we simulated new data sets with samples ranging from 5 to 50. Model slopes were tested for significant differences using analysis of covariance. Each new model was simulated 50 times, and the total number of significant models counted. The first sample size where the computed power was greater than 0.8 was determined. 14 subjects are required per arm to achieve a power of 0.8 for the loudness test, and 17 subjects are required per arm for the TFI. Thus, this study design requires a total subject count of 34. We propose a minimum sample size of 50 to account for subject variability and compliance.

Due to the COVID-19 pandemic, study enrollment and in-clinic evaluations were paused from March 2020 through August 2020 due to University of Michigan mandates. As a result, participants were no longer seen at the Investigational Site. Consequently, a portion of participants who already had enrolled in the study were not able to return to the site to complete Tinnitus Loudness measurements and audiometric testing. Since the 6-week Treatment Period Visit for the second period occurs 18 weeks after the participant starts treatment, this resulted in incomplete Tinnitus Loudness data for participants who enrolled in the study between mid-November 2019 through March 2020. The required sample size was raised from 50 to 100 to account for these missing data.

9 General Analysis Considerations

9.2 Timing of Analyses

Unblinding will occur when 100 subjects have completed the final Post-study Monitoring survey 3 or dropped out before then. The final study analysis will occur post-unblinding.

9.3 Analysis Populations

We will perform both per-protocol and intention-to-treat analyses. While intention-to-treat analysis is ideal, our protocol may require subjects to adhere to a strict schedule to fully realize the benefit of the treatment. Our pilot study had excellent compliance rates, but a longer and larger study provides more opportunity for non-compliant subjects. Should sensitivity analysis between the intention-to-treat and per-protocol analyses attain significance, differences between compliant and non-compliant subjects will be further explored in an effort more fully understand who best can benefit from the bimodal protocol.

The following Analysis Cohorts will be identified depending on the type and extent of analysis being performed. *Study Success will be based on the PP Cohort.*

9.3.3 Safety/Intent-to-treat (ITT) Cohort

All participants who are randomized and start the treatment with the Active or Control programming in the first treatment period are included in the Safety/ITT Cohort. Analyses for all safety related endpoints will be performed using the Safety/ITT Cohort.

9.3.4 Modified Intent-to-treat Cohort (mITT)

All participants who are randomized and start the treatment with the Active or Control

programming in the first treatment period and have evaluable data at the 6-Week Treatment Period Visit for both treatment periods are included in the mITT Cohort.

9.3.5 Per-Protocol Cohort (PP)

The Per-Protocol Cohort is defined as the group of mITT participants who complete their 6-week post second treatment period follow-up visit without any protocol violations (PV).

9.3.6 Cohort for Primary and Secondary Endpoints

Analyses of all primary and secondary effectiveness endpoints will be repeated for the ITT, mITT, and PP Cohorts.

9.4 Covariates and Subgroups

9.4.1 Analysis framework.

A linear mixed effects regression (LMER) model will be developed for each primary endpoint with random and fixed effects. Treatment order (active first vs control first), treatment condition (active, active washout, control, control washout), baseline measurement, and interaction of treatment and treatment order will be treated as fixed while subject ID is included as a random effect.

9.4.2 Model covariates:

1. Age at enrollment (continuous variable; years)
2. Sex (categorical; male or female)
3. Covid-lockdown period
    a. Treated as a categorical variable.
    b. Pre, During, or Post-resumption of in-lab activities

9.4.3 Exploratory analysis variables

1. Duration of tinnitus (continuous variable; months)
2. Race/ethnicity (categorical variable; white or non-white)
3. Pure-tone average (PTA) threshold for left and right ears (continuous variable; dB SPL)
4. Tinnitus VAS loudness (continuous variable; VAS-scale)

9.4.4 Demographic factors:

1. Age at enrollment
2. Sex
3. PTA
4. Race/ethnicity

9.5 Missing Data

A Linear Mixed Effects Regression (LMER) model will be utilized. LMER is appropriate when analyzing repeated measures as accounts for within-subject correlation. LMER analysis can account for missing data in imbalanced Sequence Groups and missing observations from subjects.

Sensitivity analyses will be performed to evaluate the robustness of the primary effectiveness results. Several different methodologies to impute missing data will be used to evaluate the robustness of the results:

1. A worst-case approach will be used to impute values for missing visits. If a participant is missing data at the visit for the endpoint, the baseline value will be imputed for the missing value.
2. Treatment differences will be evaluated as a between-participant effect by analyzing

data collected during Treatment Period 1.

Other methodologies, such as multiple imputation methods, will be explored and utilized, as appropriate.

9.6 Multiple Testing

Exact confidence intervals will be generated for estimates of proportions. Asymptotic confidence intervals will be generated for estimates of means. Except where otherwise noted, the p-values of all tests will be reported without any correction for the multiplicity of tests performed. Given that endpoint outcomes are defined *a priori* on a restricted set of models, multiple comparisons are not required.

10 Summary of Study Data

Categorical data will be summarized using frequency tables, presenting the subject counts and relative percentages. The Chi-Square test will be used to compare the percent of participants between treatment groups. McNemar's chi-square may be used to assess within-participant changes in a bivariate response variable.

Continuous variables will be summarized by the mean, standard deviation, median, minimum, and maximum. Unless otherwise stated, LMER models with Treatment as the within-participant factor and Sequence as the between-participant factor will be used to compare differences in the means between treatment groups. In addition to the coefficients reported by the LMER model, between-group changes within a treatment period were estimated via linear combinations of coefficients from the LMER model. Within-participant changes (i.e., Change-from-Baseline) within a treatment period will be analyzed parametrically using the Paired t-test if the differences are normally distributed, or non-parametrically using the Sign-Rank Test if the differences are not normally distributed.

Only deviations from the general overview will be noted in the subsequent sub-sections within section 9.

10.1 Subject Disposition

The summary statistics will be produced in accordance with section 9.

10.2 Derived variables

Derived Variables include:

- Tinnitus loudness, sensation level (SL). Per-week changes in a subject's tinnitus loudness and the same subject's most recent audiogram will be reported. The subject's audiogram will be interpolated at the set of tinnitus loudness frequencies through linear interpolation.

10.3 Protocol Deviations

Modifications to the study protocol will not be implemented by either the sponsor or the investigator without agreement by both parties. However, the investigator may implement a deviation form, or a change of, the protocol to eliminate an immediate hazard(s) to the trial subjects without prior IRB/sponsor approval/favorable opinion. As soon as possible, the implemented deviation or change, the reasons for it and if appropriate the proposed protocol amendment should be submitted to the IRB and sponsor.

Deviations from the protocol must be recorded and reported (per local IRBMED guidelines) by the investigator. The circumstances, action taken and impact of major deviations on the trial must be communicated by the Principal Investigator to the designated ISM.

The summary statistics will be produced in accordance with section 9.

10.4 Demographic and Baseline Variables

The summary statistics will be produced in accordance with section 9.

10.5 Concurrent Illnesses and Medical Conditions

The summary statistics will be produced in accordance with section 9.

10.6 Treatment Compliance

The summary statistics will be produced in accordance with section 9.

11 Efficacy Analyses

For all primary (Tinnitus loudness, TFI and THI), secondary and exploratory endpoints, the following approach will be utilized:

- LMER analysis, including random and fixed effects, and covariates, will be performed.
- Continuous endpoint measures are repeated measures and will be listed by subject. Data will be summarized by treatment group and treatment period. N, Mean, Standard Deviation, Minimum and Maximum will summarize continuous efficacy variables,
- Categorical efficacy variables will be summarized by count and percent.

Tinnitus loudness is measured by having subjects match the loudness and likeness of their tinnitus to one of 11 sounds with different center frequencies. Absolute differences are averaged relative to baseline to determine loudness. TFI and THI are questionnaires to be completed at each weekly visit. Both are measured on an interval scale.

Analyses of all primary and secondary effectiveness endpoints will be repeated for the ITT, mITT, and PP Cohorts. Study Success will be based on the PP Cohort.
Adjustments for Type I error over the set of endpoints is unnecessary as the measures are not directly related.

11.1 Primary Efficacy Analysis

The summary statistics will be produced in accordance with section 9.

11.2 Secondary Efficacy Analyses

The summary statistics will be produced in accordance with section 9.

11.2.1 Secondary Analyses of Primary Efficacy Endpoint

The summary statistics will be produced in accordance with section 9.

11.2.2 Analyses of Secondary Endpoints

The following analytical framework will be applied to secondary measures (see section 9 for more details). Analyses are not dependent on primary-measures. All measures are

continuous measures. Data will be analyzed using LMER models and shown as mean+/-SEM. Normality will be assessed using Kolmogorov-Smirnoff tests, and non-parametric tests used for non-normally distributed data. To assess how the secondary measures might co vary with the primary measures, we will use principal component analysis, correlations, or linear fixed effects models.

11.3 Exploratory Efficacy Analyses

The summary statistics will be produced in accordance with section 9.

12 Safety Analyses

Safety Analyses will be performed on the Safety/ITT Cohort.

When calculating the incidence of adverse events, each subject will be counted once, and any repetitions will be ignored; the denominator will be the total population size. All AEs will be grouped by device-relatedness and severity, with summary statistics performed per treatment arm.

A table summarizing adverse events will be produced:

- All-Cause Mortality: all anticipated and unanticipated deaths due to any cause, with number and frequency of such events in each arm of the clinical study.

- Serious Adverse Events: all anticipated and unanticipated serious adverse events, grouped by device-relatedness, with number and frequency of such events in each arm of the clinical study.

- Other (Not Including Serious) Adverse Events: anticipated and unanticipated events (not included in the serious adverse events) within any arm of the clinical study, grouped by device-relatedness, with number and frequency of such events in each arm of the clinical study.

Only deviations from the analytical and summary approaches will be noted in the subsequent subsections of section 11.

12.1 Extent of Exposure

The summary statistics will be produced in accordance with section 9.

12.2 Adverse Events

The summary statistics will be produced in accordance with section 9.

12.3 Deaths, Serious Adverse Events, and other Significant Adverse Events

The summary statistics will be produced in accordance with section 9.

12.4 Pregnancies (As applicable)

The summary statistics will be produced in accordance with section 9.

12.5 Prior and Concurrent Medications

The summary statistics will be produced in accordance with section 9.

12.6 Other Safety Measures
The summary statistics will be produced in accordance with section 9.

13 Reporting Conventions
P-values ≥0.001 will be reported to 3 decimal places; p-values less than 0.001 will be reported as “<0.001”. The mean, standard deviation, and any other statistics other than quantiles, will be reported to one decimal place greater than the original data. Quantiles, such as median, or minimum and maximum will use the same number of decimal places as the original data. Estimated parameters, not on the same scale as raw observations (e.g., regression coefficients) will be reported to 3 significant figures.

14 Quality Assurance of Statistical Programming
At the time of writing,

1. The population to be used in a table or figure will be explicitly set at the start of a block of code that computes the output, by looking up the population from the main data table.
2. Any outputs will have the:
    a. date and time of generation and last modification
    b. the name of the code file that produced the analysis.
    c. the author(s) of the code file
3. At the start of any code file there will be a set of comments that give:
    a. the author(s) of the code file
    b. the date and time of writing
    c. references to inputs and outputs
    d. reference to any parent code file(s) that runs the child code file.
4. The following software packages were used on Windows 10 workstations:
    a. the Mathworks MATLAB with the *Statistics and Machine Learning* Toolboxes (version >=R2020a),
    b. the R programming language (version >= 4.2)
    c. the Python programming language (version >=3.6)
5. Primary Data are captured and stored on the Redcap platform at the University of Michigan (https://redcapproduction.umms.med.umich.edu/)
    a. Tinnitus psychophysical measures will be captured using *TinnTester.* Local data copies are saved on the workstation and uploaded to redcap manually by study investigators.
    b. All other data measurements are directly saved onto Redcap through its automated user interface and survey capture tools.
    c. All analysis scripts and secondary data are saved on shared Dropbox Team folders owned by the University of Michigan.

15 Summary of Changes to the Protocol and/or SAP

Table of Protocol Changes:

Protocol Section	Summary of Revisions Made	Rationale
**1.2**	Updated both schemas in Section 1.2 of the protocol	The schema on page 6 shows the End of Study visit occurring prior to the washout,

		but this visit happens after the washout, as shown in Section 1.3.<br>The schema on page 7, in the Visit 15-28 Time Point section, states the final washout is 4 weeks, but it is 6 weeks, as shown in Section 1.3
**1.1**	Added an exclusion criterion	Added "pulsating" because this type of tinnitus is not what the device has been tested on with the animals.
**8.1**	Deleted "within 30 days."	Give subjects more flexibility for when is best to begin the study with their schedule or if they need to wait on an MRI.
**6.1.2**	Added "Completion of Arm 1 and … flexibility for subjects."	Give subjects flexibility to remain in study, if they must miss a chunk of time due to a family emergency, job, etc.
**8.1**	Added section under Specific Procedures: Otologic examination and medical case history regarding mental health identification	If mental health issues are identified during screening process, subjects will be referred to their PCP for further evaluation and/or follow-up.
**7.3**	Added sentence defining missed visits and protocol deviation	Clarifying that one missed visit per 6-week time does not qualify as a protocol deviation.
**5.5**	Added section detailing remote consenting	Subjects allowed to consent remotely to reduce unnecessary in-person visits
**5.1**	Expanding hearing criteria	Minor expansion of hearing threshold criteria to include more subjects in the study
	Expanding consenting number from 300 to 400	Allows more subjects to be consented to reach goal for active subjects
	Expanding randomized subjects from 50 to up to 100	Subjects that were enrolled during pandemic shutdown were not able to participate completely and have incomplete data, allows us to reach goal of 50 subjects with complete data
**7.1, 8.2**	Clarifying exclusion criteria	Quantifying exclusion criteria relative to week-over-week changes in TFI and TT
**8.2**	Adding online locations, and 2nd floor of Med Sci I for medical screening, and Dr. Stucken for referrals as needed	Allows to meet screen and consent subjects online and prevent excessive in person appointments.<br>Allow Emily Stucken to refer as needed
**8.2**	Updating adverse event reporting to match IRBMED	Updating adverse event reporting timeline from 7 days to 14 days to match IRBMED

16 References

1. Marks KL, Martel DT, Wu C, Basura GJ, Roberts LE, Schvartz-Leyzac KC, Shore SE. Auditory-somatosensory bimodal stimulation desynchronizes brain circuitry to reduce tinnitus in guinea pigs and humans. Sci Transl Med. 2018;10(422). doi: 10.1126/scitranslmed.aal3175. PubMed PMID: 29298868.
2. Roberts LE, Moffat G, Baumann M, Ward LM, Bosnyak DJ. Residual inhibition functions overlap tinnitus spectra and the region of auditory threshold shift. J Assoc Res Otolaryngol. 2008;9(4):417-35. doi: 10.1007/s10162-008-0136-9. PubMed PMID: 18712566; PMCID: 2580805.
3. Hoare DJ, Edmondson-Jones M, Gander PE, Hall DA. Agreement and reliability of tinnitus loudness matching and pitch likeness rating. PLoS One. 2014;9(12):e114553. doi: 10.1371/journal.pone.0114553. PubMed PMID: 25478690; PMCID: PMC4257689.
4. Hall DA, Pierzycki RH, Thomas H, Greenberg D, Sereda M, Hoare DJ. Systematic Evaluation of the T30 Neurostimulator Treatment for Tinnitus: A Double-Blind Randomised Placebo-Controlled Trial with Open-Label Extension. Brain Sci. 2022;12(3). doi: 10.3390/brainsci12030317. PubMed PMID: 35326273; PMCID: PMC8946033.
