# Supplementary material for: Reversing Synchronized Brain Circuits Using Targeted Auditory-Somatosensory Stimulation to Treat Phantom Percepts: A Randomized Clinical Trial
Source: JAMA Netw Open. 2023 Jun 2;6(6):e2315914. doi: 10.1001/jamanetworkopen.2023.15914 (PMC10238951; doi:10.1001/jamanetworkopen.2023.15914)
Supplement: Supplement 3. — Data Sharing Statement [file jamanetwopen-e2315914-s003.pdf]

Data Sharing Statement

Jones. Reversing Synchronized Brain Circuits Using Targeted Auditory-Somatosensory Stimulation to Treat Phantom Percepts. *JAMA Netw Open*. Published June 02, 2023. doi:10.1001/jamanetworkopen.2023.15914

Data
Data available: Yes
Data types: Deidentified participant data, Data dictionary
How to access data: sushore@umich.edu; damartel@umich.edu
When available: With publication

Supporting Documents
Document types: Statistical/analytic code, Informed consent form
How to access documents: sushore@umich.edu; damartel@umich.edu (These were uploaded with the original submission) URL will be available on publication
When available: With publication

Additional Information
Who can access the data: Those with an interest - academic or clinical researchers
Types of analyses: For future research
Mechanisms of data availability: with signed data access agreement
